# The impact of the timely birth dose vaccine on the global elimination of hepatitis B

**DOI:** 10.1038/s41467-021-26475-6

**Published:** 2021-10-28

**Authors:** Margaret J. de Villiers, Shevanthi Nayagam, Timothy B. Hallett

**Affiliations:** 1 MRC Centre for Global Infectious Disease Analysis, School of Public Health, Imperial College London, London, UK; 2 Section of Hepatology & Gastroenterology, Department of Metabolism, Digestion & Reproduction, Imperial College London, London, UK

**Keywords:** Computational models, Hepatitis B, Epidemiology

## Abstract

In 2016 the World Health Organization set the goal of eliminating hepatitis B globally by 2030. Horizontal transmission has been greatly reduced in most countries by scaling up coverage of the infant HBV vaccine series, and vertical transmission is therefore becoming increasingly dominant. Here we show that scaling up timely hepatitis B birth dose vaccination to 90% of new-borns in 110 low- and middle-income countries by 2030 could prevent 710,000 (580,000 to 890,000) deaths in the 2020 to 2030 birth cohorts compared to status quo, with the greatest benefits in Africa. Maintaining this could lead to elimination by 2030 in the Americas, but not before 2059 in Africa. Drops in coverage due to disruptions in 2020 may lead to 15,000 additional deaths, mostly in South-East Asia and the Western Pacific. Delays in planned scale-up could lead to an additional 580,000 deaths globally in the 2020 to 2030 birth cohorts.

## Abstract (web summary)

The timely hepatitis B birth dose vaccination is recommended for all new-borns by the WHO, but coverage is inconsistent. Here, the authors model the impact of scaling-up coverage in 110 low and middle income countries and assess how it may be affected by delays for example caused by the COVID-19 pandemic.

## Introduction

Nearly 40 years since the hepatitis B virus (HBV) vaccine was introduced in the 1980s, HBV infection continues to pose a significant public health threat, which disproportionately affects low and middle-income countries (LMICs). Nearly 250 million people are estimated to be living with chronic HBV infection globally and the virus causes nearly 900,000 deaths a year due to hepatocellular carcinoma (HCC) and cirrhosis^1^.

In 2016, the World Health Assembly endorsed a World Health Organization (WHO) strategy to eliminate HBV as a public health threat by 2030^2,3^, measured by the reduction of HBV surface antigen (HBsAg) prevalence in five-year-olds to 0.1% by 2030. Accordingly, targets were established that included scaling up coverage of the infant HBV vaccine series (HepB3), which reduces horizontal transmission, and the timely birth dose (timely HepB-BD: a dose of monovalent vaccine administered within 24 h of birth to all new-borns), which primarily reduces mother-to-child transmission (MTCT)^4–10^, each to 90% by 2030.

This is close to being achieved for HepB3, which is usually integrated within the Expanded Programme on Immunization schedule and is funded by Gavi, the Vaccine Alliance (GAVI), in many low-income settings (https://www.gavi.org/programmes-impact/our-impact/countries-approved-support, accessed in March 2021): globally, coverage was 85% of infants in 2019, although it was only 73% in the same year in the WHO’s Africa region (AFRO; https://cdn.who.int/media/docs/default-source/immunization/global_monitoring/slidesglobalimmunization.pdf?sfvrsn=25385c3b_7, accessed in May 2021). However, despite the targets and although the WHO has recommended since 2009 that all new-borns receive the timely HepB-BD vaccine, irrespective of the mother’s serological status, scale-up of the timely HepB-BD has been slow, coverage heterogenous, and there has not been the same funding support from GAVI. By 2019, only an estimated 43% of new-borns had received timely HepB-BD vaccine (~6% in the WHO AFRO region; https://cdn.who.int/media/docs/default-source/immunization/global_monitoring/slidesglobalimmunization.pdf?sfvrsn=25385c3b_7, accessed in May 2021) and only 110 countries were administering the universal HepB-BD vaccine to new-borns as part of their national policy (https://immunizationdata.who.int/pages/vaccine-intro-by-antigen/hepb_bd.html?ISO_3_CODE=&YEAR=, accessed in June 2021). There are concerns that the timely HepB-BD is difficult to implement and not necessary for reducing the hepatitis burden to very low levels in some settings^11,12^.

In 2019, GAVI re-evaluated extending their funding to provide catalytic support for introducing the timely HepB-BD vaccine (https://www.gavi.org/our-alliance/strategy/vaccine-investment-strategy, accessed in January 2021). However, the COVID-19 pandemic and the response to it have created additional barriers to scaling up timely HepB-BD coverage, both due to disruptions in healthcare facilities^13–16^, which have affected routine immunization and facility-based births^17,18^, and indirectly due to changes in priorities and funding.

In this work, we show that scaling up timely HepB-BD vaccination coverage to 90% by 2030 results in immediate reductions in incident chronic HBV cases and HBsAg prevalence in five-year-olds, but delayed reductions in HBV-related deaths and disability adjusted life years (DALYs). Since current timely HepB-BD coverage tends to be low in African countries, these countries will benefit most from scale-up in timely HepB-BD. Elimination of HBV cannot be achieved by 2030 in most geographical regions in the vaccination scenarios analysed in this study (which all involve HepB3 maintained at status quo levels). Disruptions in vaccination efforts in 2020 due to COVID-19 will not delay HBV elimination, but will result in an increase in HBV-related deaths in the 2020 birth cohort. Delays in the scale-up of timely HepB-BD coverage will result in both delays in the elimination of HBV and in substantial increases in HBV-related deaths in the following decades.

## Results

**Fig. 1:**
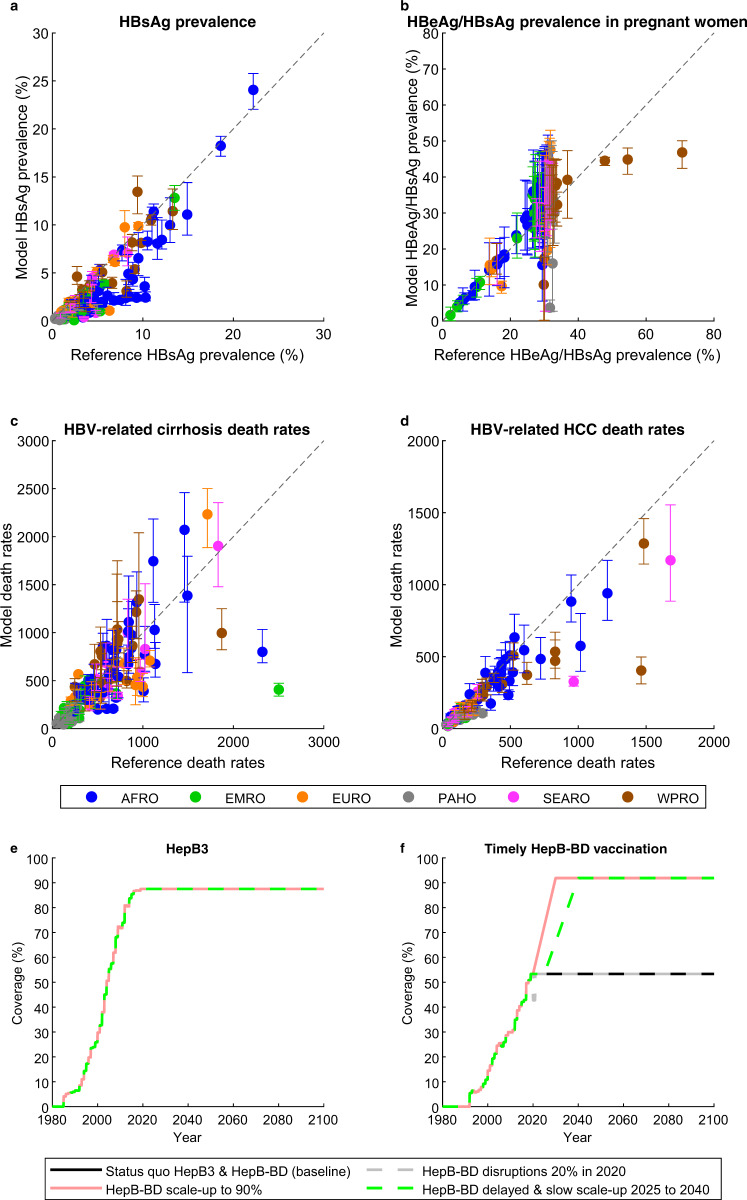
The model calibrations and the scenarios used. **a** HBsAg prevalence in all ages, **b** HBeAg prevalence in HBsAg+ women of childbearing age, and HBV-related death rates (deaths per 100,000) from **c** cirrhosis and **d** hepatocellular carcinoma in the 110 countries modelled compared to data from the literature in the year the data were collected. Data are presented as mean values with 95% credibility intervals of *n* = 200 model outcomes resulting from 200 independent draws from the posterior distribution of each country. Countries modelled in the six WHO regions AFRO, EMRO, EURO, PAHO, SEARO and WPRO are given in Supplementary Fig. 1. **e** HepB3 and **f** timely HepB-BD vaccination coverage globally in selected scenarios (see Table 1). Note that all four lines co-incide in Fig. 1e. In Fig. 1f, the Status quo HepB3 & HepB-BD (baseline) line appears dashed where it co-incides with the HepB-BD disruptions 20% in 2020 line and the HepB-BD scale-up to 90% line appears dashed where it co-incides with the HepB-BD delayed & slow scale-up 2025 to 2040 line. HBeAg: hepatitis B e antigen; HBsAg: hepatitis B surface antigen; HBV: hepatitis B virus; HCC: hepatocellular carcinoma; HepB3: infant HBV vaccine series; timely HepB-BD: timely birth dose; WHO: World Health Organization.

We use a set of models representing the HBV epidemic in each of 110 LMICs, which together represent all six WHO regions and include all current GAVI-eligible countries (Supplementary Fig. 1). Each country model was calibrated to data (Supplementary Table 1) on HBsAg and HBV e antigen (HBeAg) seroprevalence, HBV-related deaths due to cirrhosis and HCC, and national data on coverage of vaccinations and treatment (Fig. 1a–d). We apply a set of scenarios for the future coverage of the timely HepB-BD in each country (Table 1) and quantify impact with reference to the infections and deaths that would occur in a scenario in which the coverage of timely HepB-BD remains at the level recorded for each country in 2019 (the status quo HepB3 & HepB-BD scenario). These results were aggregated across country-level simulations to generate results for the six WHO regions (Supplementary Fig. 1) and global results. Figure 1e, f show the global coverage of HepB3 and timely HepB-BD vaccination (aggregated over country-level coverage values, weighted by population size in 2025) under a selection of these scenarios (Supplementary Fig. 2 gives the breakdown by WHO region for status quo, in which both HepB3 and timely HepB-BD coverage are maintained at their levels in 2019 until 2100).

**Table 1:** Vaccination scenarios.

Scenario	Timely birth dose vaccination
Status quo HepB3 & HepB-BD	Maintained at level recorded for 2019, throughout the period 2020–2100.
HepB-BD scale-up scenario: timely HepB-BD expansion to ≥90%^a^ by 2030	Linear expansion from 2019 level to the target value^b^ by 2030 and maintained at that level until 2100.
HepB-BD disruptions scenario: drop in timely HepB-BD coverage in 2020 by a) 5%, b) 10%, c) 15%, d) 20% due to disruptions associated with COVID-19	Drop of target value in the year 2020 relative to the level recorded for 2019; maintained at level recorded in 2019 in the period 2021–2100.
Delayed HepB-BD scale-up scenario: timely HepB-BD expansion delayed & ≥90% coverage reached over the period a) 2023 to 2030, b) 2023 to 2033, c) 2025 to 2040	Maintained at level recorded for 2019 until starting year of expansion; linear expansion from the level recorded for 2019 to ≥90% over the expansion period and maintained at that level until 2100.

Note: For all scenarios, timely HepB-BD coverage and HepB3 coverage reflect data from WHO/UNICEF Estimates of National Immunization Coverage (https://apps.who.int/immunization_monitoring/globalsummary/timeseries/tswucoveragehepb3.html and https://apps.who.int/immunization_monitoring/globalsummary/timeseries/tswucoveragehepb_bd.html, accessed in July 2020) through to the end of the year 2019, and HepB3 coverage is maintained at the level recorded for 2019 in each country from 2019 to 2100. HBV: hepatitis B virus; HepB3: infant HBV vaccine series; timely HepB-BD: timely birth dose; UNICEF: United Nations Children’s Fund; WHO: World Health Organization.

^a^Note that results from target values other than ≥90% i.e. ≥25%, ≥50%, ≥75%, are presented in Supplementary Tables 3–6.

^b^Scale-up of coverage is always to the greater of the target expressed or the value recorded for each country in 2019.

### The impact of scaling up the timely birth dose

**Fig. 2:**
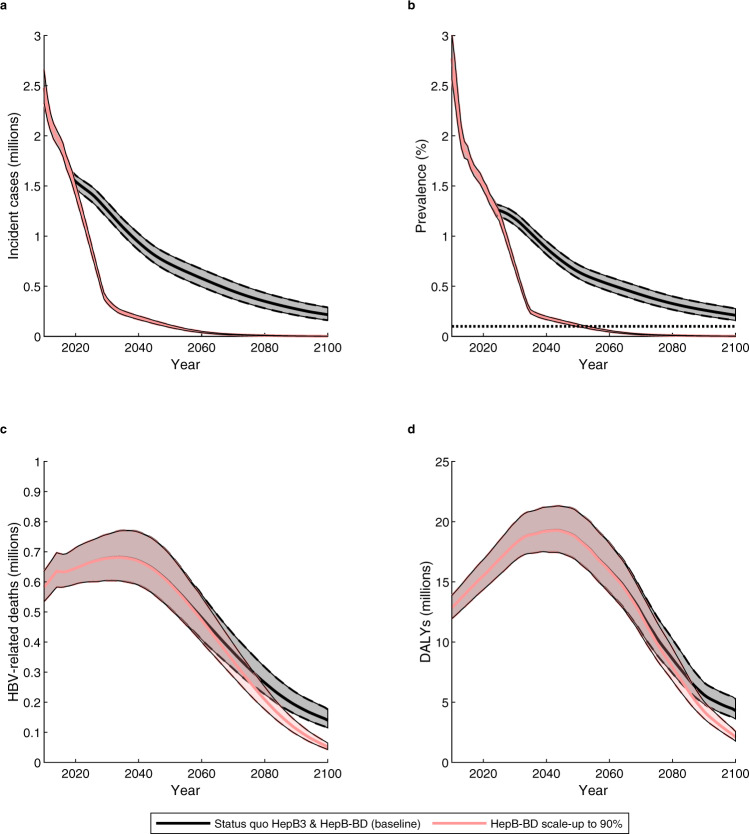
The overall impact of timely HepB-BD scale-up on disease burden globally. **a** Incidence of new chronic carriage of HBV, **b** Prevalence of chronic HBsAg among five-year-olds, **c** HBV-related deaths and **d** total DALYs incurred. Shown are the means (lines) and 95% credibility intervals (shaded areas), comparing the status quo HepB3 & HepB-BD scenario (black line; grey shading) with a scenario in which timely HepB-BD is given to ≥90% of new-borns (the HepB-BD scale-up scenario; red line and shading). Results are the sum from all modelled countries of *n* = 200 model outcomes resulting from 200 independent draws from the posterior distribution of each country. The horizontal dashed line in Fig. 2b represents the WHO elimination threshold of 0.1% HBsAg prevalence in five-year-olds. DALY: disability adjusted life years; HBsAg: hepatitis B surface antigen; HBV: hepatitis B virus; timely HepB-BD: timely birth dose; WHO: World Health Organization.

Figure 2 shows the modelled impact of scaling up timely HepB-BD vaccination to ≥ 90% by 2030 (the HepB-BD scale-up scenario) on HBV disease burden globally. The scale-up results in immediate reductions in chronic HBsAg incidence and the prevalence of HBsAg among five-year-olds relative to the status quo HepB3 & HepB-BD scenario, greatly accelerating the gradual reductions that would be expected otherwise (Fig. 2a, b). The WHO elimination target (less than 0.1% HBsAg prevalence in five-year-olds by 2030) cannot be reached globally in the countries modelled (before 2100) without the scale-up of the timely HepB-BD. However, with the modelled fast and substantial scale-up of timely HepB-BD, that target can be reached in 2052 (2050 to 2054; Fig. 2b). Scaling up timely HepB-BD vaccination to ≥ 90% by 2030 would avoid 41,000,000 (36,000,000 to 46,000,000) chronic infections relative to the status quo HepB3 & HepB-BD scenario between 2020 and 2100. This scale-up results in 710,000 (580,000 to 890,000) fewer deaths among those born between 2020 to 2030 globally (Supplementary Table 3). However, this would not be recorded as a drop in deaths (Fig. 2c) or DALYs (Fig. 2d) in calendar-time until ~2050. This is because HBV usually takes decades to progress from infection to death. The expected trend in deaths and DALYs is an increase until 2030-2040, followed by a fall due to the competing effects of population ageing and growth (and so more people in older cohorts reaching ages when they are at risk of death caused by HBV) and the effect of HepB3 (reducing infections in younger cohorts).

**Fig. 3:**
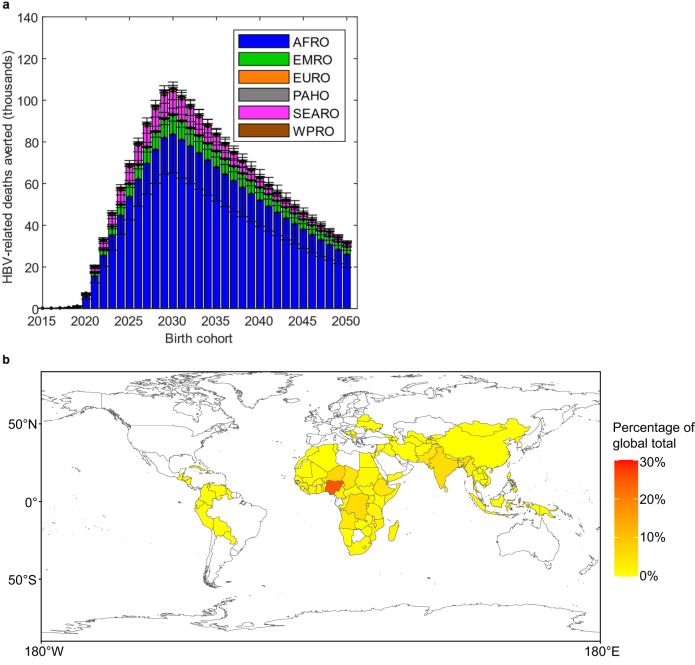
The impact of timely HepB-BD scale-up on HBV-related deaths. **a** HBV-related deaths averted in each WHO region (mean and 95% credibility intervals) in the birth cohorts for years 2015 to 2050, in the scenario in which timely HepB-BD coverage is scaled up to ≥90% by 2030 in each country (the HepB-BD scale-up scenario) compared to the status quo HepB3 & HepB-BD scenario. Results are the sums within WHO regions from all modelled countries of *n* = 200 model outcomes resulting from 200 independent draws from the posterior distribution of each country. **b** Percentage of global total HBV-related deaths averted in the 2020 to 2030 birth cohorts that occurs in each country if timely HepB-BD coverage is scaled up to ≥90% by 2030 (the HepB-BD scale-up scenario) relative to the status quo HepB3 & HepB-BD scenario. The six WHO regions AFRO, EMRO, EURO, PAHO, SEARO, and WPRO are shown in Supplementary Fig. 1. HBV hepatitis B virus, HepB3 infant HBV vaccine series, timely HepB-BD timely birth dose, WHO World Health Organization.

Figure 3 identifies the populations in which the effect of timely HepB-BD scale-up in reducing deaths is most strongly concentrated. For cohorts born before 2030, the number of deaths averted rises in line with the assumed increases in coverage of the timely HepB-BD. Later cohorts benefit somewhat less, as the risk of infection to them is lower as a result of the declining HBV prevalence among women of child-bearing age. Most (>70%) of the deaths averted in the 2020 to 2030 birth cohorts are in the WHO’s Africa region (AFRO), where HBV prevalence is very high in many countries (Fig. 1a) and current timely HepB-BD coverage is the lowest of all the WHO regions (~7%: see Supplementary Fig. 2). In fact, 25% of all deaths averted globally in the 2020 to 2030 birth cohorts would be in a single country—Nigeria—which has a large and rapidly growing population with very low timely HepB-BD coverage currently (0% in 2019). Large numbers of deaths averted would also be expected in the Middle East (WHO’s EMRO region) and South-East Asia (WHO’s SEARO region), which also have relatively low timely HepB-BD coverages (~34% and ~55%, respectively; Supplementary Fig. 2), but lower HBV prevalence than AFRO (Fig. 1a). Reflecting their large sizes, 6% and 4% of deaths averted globally in the 2020 to 2030 birth cohorts would be in India and Pakistan, respectively (Fig. 3b). Results for each region and country individually are presented for selected scenarios in Supplementary Tables 3–6.

### Achieving HBV elimination

**Fig. 4:**
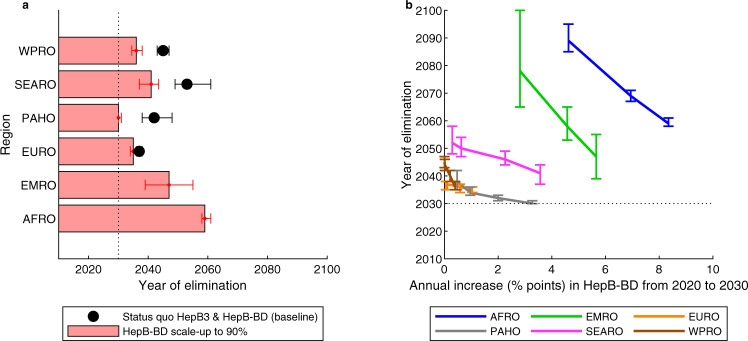
The impact of timely HepB-BD scale-up on achieving WHO elimination targets. **a** Median year by which the elimination target (0.1% HBsAg prevalence in five-year-olds) is reached in the WHO regions, with 95% credibility intervals, in the status quo HepB3 & HepB-BD scenario (black dots) and the scenario in which timely HepB-BD coverage is scaled up to ≥90% by 2030 (HepB-BD scale-up scenario; red bars). **b** Median year of elimination, with 95% credibility intervals, for different levels of annual scale-up of timely HepB-BD coverage (the HepB-BD scale-up scenarios) in the populations within each of the WHO regions. The dotted lines for the year 2030 are for reference purposes only. For each WHO region, number of HBsAg prevalent cases in five-year-olds and total number of five-year-olds were summed across countries. Number of HBsAg prevalent cases in five-year-olds was divided by total number of five-year-olds to give HBsAg prevalence in five-year-olds. The year of elimination was identified as the year in which HBsAg prevalence in five-year-olds falls below 0.1%. This was repeated *n* = 200 times using independent draws from the posterior distribution of each country. The six WHO regions AFRO, EMRO, EURO, PAHO, SEARO and WPRO are shown in Supplementary Fig. 1. HBsAg: hepatitis B surface antigen; HepB3: infant HBV vaccine series; timely HepB-BD: timely birth dose; WHO: World Health Organization.

Figure 4a shows the year by which the WHO elimination goal would be achieved for the combined populations within each region under the status quo HepB3 & HepB-BD scenario versus if the timely HepB-BD is scaled up to ≥ 90% by 2030 (the HepB-BD scale-up scenario). Without any timely HepB-BD scale-up, elimination would be reached first in Europe (WHO’s EURO region) in 2037, followed by the Americas (WHO’s PAHO region) in 2042, in both of which there is already high timely HepB-BD coverage and low prevalence of HBV, especially in the younger age groups. In contrast, in the WHO regions of EMRO and AFRO, elimination would not occur before 2100. The timely HepB-BD scale-up would bring the date of elimination earlier in all regions (with the exception of EURO, where timely HepB-BD is already at high scale) and make it possible in EMRO (in 2047) and AFRO (in 2059). With the timely HepB-BD scale-up, by 2040, the regions EURO, PAHO, and the Western Pacific (WHO’s WPRO region) would all have reached the elimination target; and by 2050, all regions except AFRO would have reached the elimination target. Results for each country are given in Supplementary Table 6.

Figure 4b shows the relationship between the annual rate of scale-up of the timely HepB-BD and the year by which elimination is reached in the populations within each of the WHO regions. Faster scale-up results in earlier elimination—especially where timely HepB-BD coverage is lowest, in the AFRO, EMRO, and SEARO regions—but no amount of accelerated scale-up would feasibly result in elimination being reached in any region by 2030 (the WHO’s target) except for in the Americas (WHO’s PAHO region). Indeed, even an infeasibly high rate of scale-up in Africa (9% points per year) would not bring the date of elimination to before 2060. This is because many countries in the region have either a very low timely HepB-BD coverage (e.g. Nigeria), a very high HBV prevalence (e.g. South Sudan (22% prevalence among all ages), Sierra Leone (19%) Liberia (15%); http://whohbsagdashboard.com/#hbv-country-profiles, accessed in January 2021, currently available at http://situatedlaboratories.net/who-hepB-dashboard/src/#global-strategies as of September 2021), or both. PAHO has the best chance of reaching the 2030 target—here moderate timely HepB-BD coverage is combined with low overall HBV, so that an accelerated scale-up of timely HepB-BD could have an effect quickly.

### The indirect effects of the COVID-19 pandemic

**Fig. 5:**
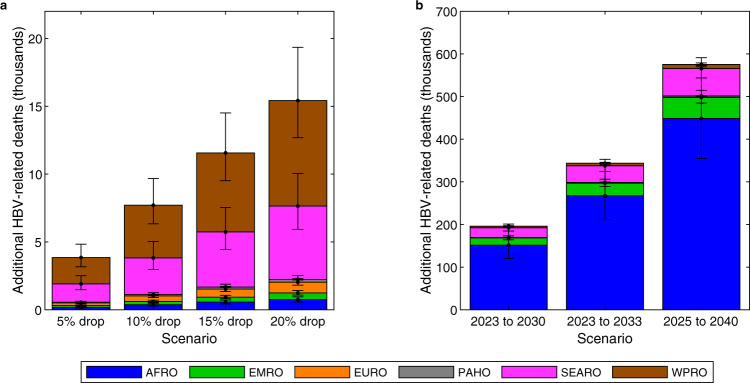
The impact of disruptions to the scale-up of timely HepB-BD on HBV-related deaths. **a** Mean additional HBV-related deaths in the birth cohorts in 2020-2030 due to a drop in the proportion of new-borns receiving timely HepB-BD in 2020 (the HepB-BD disruptions scenarios) relative to the status quo HepB3 & HepB-BD scenario, with 95% credibility intervals. **b** Mean additional HBV-related deaths in the birth cohorts in 2020-2030 due to delays in scaling up timely HepB-BD coverage to ≥90% (the delayed HepB-BD scale-up scenarios) relative to scaling up timely HepB-BD coverage to ≥90% between 2020 and 2030 (the HepB-BD scale-up scenario), with 95% credibility intervals. Results are the sums within WHO regions from all modelled countries of *n* = 200 model outcomes resulting from 200 independent draws from the posterior distribution of each country. The six WHO regions AFRO, EMRO, EURO, PAHO, SEARO, and WPRO are shown in Supplementary Fig. 1. HBV: hepatitis B virus; HepB3: infant HBV vaccine series; timely HepB-BD: timely birth dose; WHO: World Health Organization.

Figure 5a shows that a temporary drop in timely HepB-BD coverage in 2020 (HepB-BD disruptions scenarios) could result in 15,000 (12,000 to 20,000) additional HBV-related deaths, concentrated in the SEARO and WPRO regions, which have large population sizes and normally relatively high timely HepB-BD coverage levels (55% and 91% in SEARO and WPRO, respectively; Supplementary Fig. 2). The additional deaths occur mostly among those born in 2020 when the disruptions occurred, but also, to a lesser extent, to unprotected children (unvaccinated or vaccination failed) born in earlier and later years, who become infected following contact with a child born in 2020 (Supplementary Fig. 5). The effect of the temporary disruption is long-lived—the additional deaths occur mostly from 2050 onwards (Supplementary Fig. 6). However, the impact does not affect the year by which elimination is achieved (Supplementary Tables 4 and 6).

Figure 5b shows the effect that delays in scale-up of timely HepB-BD could have (the delayed HepB-BD scale-up scenarios) compared to the scale-up of timely HepB-BD (HepB-BD scale-up scenario). The nature of these disruptions could be large: at worst 580,000 (470,000 to 720,000) additional deaths globally in the 2020 to 2030 birth cohorts, concentrated mostly in AFRO, if the scale-up of timely HepB-BD to 90% only occurs between 2025 and 2040. Even if timely HepB-BD is scaled up faster after a period of delay between 2020 and 2023, there would remain a significant excess number of deaths in the cohorts that missed out on vaccination. Delays in the scale-up of timely HepB-BD lead to additional deaths that would occur mostly from 2060 onwards (Supplementary Fig. 7).

### Sensitivity analyses

Our results are sensitive to the assumptions made about other aspects of the HBV programme—HepB3 and treatment. Firstly, in the foregoing analyses it was assumed that HepB3 coverage levels will be maintained at the levels recorded in each country for 2019 as our primary aim was to explore the impact that timely HepB-BD scale-up alone could have. However, increasing HepB3 coverage levels to 100% in every country leads to fewer infections (reduction of 34% globally) and deaths (reduction of 20% globally) being averted between 2020 and 2100 as a result of scaling up timely HepB-BD to ≥ 90% by 2030 (the HepB-BD scale-up scenario) and an earlier year of elimination being reached overall (2047 versus 2052; Supplementary Table 4). This is because a somewhat higher HepB3 coverage would result in a lower risk of infection for those who did not receive the timely HepB-BD. The effects are strongest in the countries for which HepB3 coverage is currently lower (Supplementary Tables 5 and 6). For example, in Nigeria, which has a HepB3 coverage of 57% in 2019, there are 29% fewer deaths averted in the 2020 to 2030 birth cohorts in the HepB-BD scale-up scenario relative to the status quo HepB3 & HepB-BD scenario if HepB3 coverage is scaled up to 100% compared to if HepB3 coverage is maintained at status quo levels.

Secondly, whilst in the foregoing analyses it was assumed that the proportion of persons living with HBV that receive treatment remains at the same levels as those recorded in 2016 for each country, if instead treatment coverage were to increase to 40% or 80% of those eligible for treatment by 2030, then the impact (on deaths averted) of scaling up of the timely HepB-BD to ≥ 90% by 2030 is greatly reduced (globally by 42% or 77%, respectively). Similarly, the impact (on deaths averted) of drops in timely HepB-BD coverage relative to the status quo HepB3 & HepB-BD scenario is reduced (globally the impact (on deaths averted) of a 20% drop in timely HepB-BD is reduced by 31% or 70%, respectively), as is the impact (on deaths averted) of delays in timely HepB-BD coverage relative to scaling up the timely HepB-BD to ≥ 90% by 2030 (globally the impact (on deaths averted) of scaling up timely HepB-BD to ≥ 90% between 2025 and 2040 is reduced by 41% or 76%, respectively). This is because, with treatment, the risk of death following infection is reduced substantially.

## Discussion

HepB3 coverage is already very high, having a global coverage of over 80%, protecting large numbers of infants in most countries from horizontal transmission of HBV. However, timely HepB-BD coverage is still low in many places, especially in low and lower-middle income countries where the burden is concentrated, and this is the major impediment to eliminating HBV. Scaling up timely HepB-BD is an effective way of reducing HBV burden in countries, and our results confirm the WHO’s emphasis on the importance of scaling up the timely HepB-BD. The scale-up consistent with the WHO target (≥90% timely HepB-BD coverage by 2030) could reduce HBV-related deaths amongst the 2020 to 2030 birth cohorts globally by 710,000 (580,000 to 890,000). This impact is especially great in the AFRO region, which has the highest HBsAg prevalence and the lowest timely HepB-BD coverage. However, timely HepB-BD coverage is currently very low in countries in AFRO, and it may be a more realistic goal to scale up timely HepB-BD to ≥25% by 2030, which would avert 150,000 (120,000 to 190,000) deaths in AFRO in the 2020 to 2030 birth cohorts compared to the status quo HepB3 & HepB-BD scenario (Supplementary Table 3).

The analyses show it will not be possible to achieve HBV elimination by 2030 (the WHO’s stated ambition for HBV elimination) under the scenarios presented in this paper in any region except for PAHO. Instead, 2040 is now a more realistic goal for the countries modelled in EURO and WPRO, 2050 for EMRO and SEARO, and 2060 for AFRO. However, elimination might be achieved faster if HepB3 coverage were scaled up beyond its level in 2019 and/or if timely HepB-BD coverage were scaled up faster than in the scenarios in these analyses. Additional interventions such as increased testing (for HBsAg +/− HBV DNA or HBeAg^19^) followed by the scale-up of antiviral treatment and the expansion of programmes to provide peripartum antiviral prophylaxis to HBsAg+ pregnant women with high viral loads^20^, which are highly effective in reducing the risk of HBV MTCT^21–24^, could speed up elimination even further in particular countries^25^ or even some WHO regions^26^.

Our findings are consistent with GAVI’s conclusion on the favourability of expanding funding to provide catalytic support for introducing the HBV timely HepB-BD vaccine (https://www.gavi.org/our-alliance/strategy/vaccine-investment-strategy, accessed in January 2021). However, we note that such an expansion has been delayed due, in part, to the circumstances created by the COVID-19 pandemic^27^. This is deeply unfortunate, as delays in the scale-up lead to large numbers of additional avoidable deaths especially in Africa (Fig. 5b) that cannot be mitigated by an accelerated scale-up in later years. This indirect effect of the COVID-19 pandemic dwarves another type of effect—temporary disruptions in the on-going delivery of timely HepB-BD (Fig. 5a)—which has attracted more attention hitherto^28^. For example, a recent study^28^ modelled the impact of disruptions caused by the COVID-19 pandemic on childhood immunization of several diseases in Africa, including HepB3. That study found that a suspension of childhood vaccination programmes for six months without any subsequent catch-up campaigns could cause 3,827 deaths in the unvaccinated children before the age of 5 years in Africa (although that is bound to be an underestimate of HBV-related deaths because HBV tends to take decades to progress from infection to death).

In addition to the COVID-19 pandemic, there are further challenges to scaling up timely HepB-BD to 90% by 2030. Since the birth dose vaccine should be administered within 24 h of birth to have maximum efficacy, home births, especially in rural settings, present challenges to vaccinating new-borns in this time-critical manner. Alternative solutions including outreach interventions or the use of HB-Uniject, which is a pre-filled, auto-disable injection device, could facilitate administration of timely HepB-BD in these settings. Another challenge is that birth dose vaccine should be stored between 2 °C and 8 °C. However, provided that the vaccine is administered soon afterwards, the birth dose vaccine has been found to be heat-stable if it is exposed only once to high temperatures for a limited period of time^6,29,30^. Integrating timely HepB-BD with other vaccinations such as bacillus Calmette-Guérin tuberculosis and oral polio vaccines or other new-born interventions could facilitate and reduce the health opportunity cost of introducing an HBV timely HepB-BD programme^31^.

Limitations of this current analysis include the paucity of historical and current prevalence and treatment data, which compromises the certainty with which projections can be made. Prevalence in sub-Saharan Africa, particularly amongst children, is especially uncertain^32,33^, making the estimates for some African countries less reliable. Furthermore, models of HBV have to rely on assumptions for the natural history of infection from a variety of studies from different international settings, despite concerns population differences have a material effect on risk of transmission and disease progression^34–40^. Most notably, we have assumed an 70–95% efficacy of timely HepB-BD vaccination amongst those born to HBeAg+ mothers, which is based primarily on data from Asian studies. However, a systematic review^10^ found that in Africa, based on pooled data from a very limited number of studies, the timely HepB-BD has much less effect on the risk of transmission. Therefore, our analysis might overestimate impact in the AFRO region if efficacy really is lower than in Asia. Another limitation of the study is that co-infections, such as with HIV and hepatitis D^41^, were not taken into account: if they had been, we would expect to estimate a greater number of deaths averted by timely HepB-BD scale-up. Moreover, there is evidence that patients infected vertically with HBV remain highly infectious for longer due to slower HBeAg loss, and may have elevated risks of progressing to cirrhosis and/or HCC^38,42–44^. This could result in more HBV-related deaths being averted by timely HepB-BD scale-up than we have estimated. Another limitation is that, due to a paucity of data, the model does not model partial immunity between successive vaccination doses. The scenarios for the disruptions due to COVID-19 (the HepB-BD disruptions scenarios and the delayed HepB-BD scale-up scenarios) are non-specific out of necessity, although they are principled on reports that are available^17^. Drops in timely HepB-BD coverage in a country could occur for a number of reasons: temporary disruptions to vaccine supplies, less availability of healthcare staff due to sickness or redeployment to provide COVID-19 relief or a reduction in the proportion of facility-births as a result of transport restrictions during lockdown or patients being fearful of catching COVID-19 in healthcare settings. Delays in the scale-up of the timely HepB-BD vaccine in a country could be the result of prolonged transport disruptions (affecting international supply chain or local delivery systems) or funds being diverted away from routine vaccines and towards fighting the COVID-19 pandemic. It will be important to determine the impact of the COVID-19 pandemic once more data on the disruptions in different countries are available. Another limitation of these analyses is that we did not model high-income countries (Supplementary Table 2). However, EURO and PAHO (which each contain several countries that were not modelled), together contain less than 10% of global prevalent (HBsAg+) cases (http://whohbsagdashboard.com/#hbv-country-profiles, accessed in January 2021, currently available at http://situatedlaboratories.net/who-hepB-dashboard/src/#global-strategies as of September 2021). Moreover, vaccine coverage tends to be higher in high-income countries (HICs) than in LMICs. Hence, our analysis is likely to only yield a slight underestimate of global HBV-related deaths. Since HICs tend to have lower HBsAg prevalences than do LMICs, including all of the EURO and PAHO countries in the analyses would probably result in EURO and PAHO each reaching elimination sooner than was found in these analyses.

Overall, the scale-up of the timely HepB-BD could lead to major gains in health and is a necessary requirement for achieving HBV elimination. Even though the WHO recommends the scale-up and GAVI has indicated willingness to help fund it, delays in the scale-up of timely HepB-BD are leading to future preventable deaths being accumulated, especially in Africa.

## Methods

### Data sources

We focus on 110 LMIC GAVI-eligible countries that together contain 92% of global HBsAg prevalent cases (Supplementary Table 2) and capture all of the HBV burden in the low-income and lower-middle income countries globally. We classify these countries according to the six WHO regions (Supplementary Fig 1).

The model was populated with country-specific demographic data (fertility rates, male-to-female sex ratios at birth, population size, migration rates, and all-cause mortality rates) from the United Nations’ 2019 World Population Prospects (https://population.un.org/wpp/Download/Standard/Population/, accessed in October 2019). Country-specific historical vaccination coverage (1980 to 2019) of HepB3 and timely HepB-BD vaccines were sourced from WHO/UNICEF Estimates of National Immunization Coverage (WUENIC) released in July 2020 (https://apps.who.int/immunization_monitoring/globalsummary/timeseries/tswucoveragehepb3.html and https://apps.who.int/immunization_monitoring/globalsummary/timeseries/tswucoveragehepb_bd.html, accessed in July 2020) (Fig. 1e, f and Supplementary Fig. 2). The exception to this is China, for which the WUENIC vaccination coverage values for 1980 to 2013 were replaced with vaccination coverage values from Cui et al.^45^. The model was fit to country-specific, age-specific HBsAg prevalence data (Supplementary Table 1) obtained from Razavi-Shearer et al.^32^, from the WHO HBsAg dashboard (http://whohbsagdashboard.com/#hbv-country-profiles, accessed in October 2019, currently available at http://situatedlaboratories.net/who-hepB-dashboard/src/#global-strategies as of September 2021) and from Cui et al.^45^ in the case of China. Razavi-Shearer et al.^32^ and the WHO HBsAg dashboard have been found to give similar HBsAg prevalences in many countries^33^. In addition to HBsAg prevalence, the model was also fit to country-specific HBeAg prevalence in HBsAg+ women of childbearing age from Razavi-Shearer et al.^32^ or region-specific HBeAg prevalence in HBsAg+ women of childbearing age from Ott et al.^46,47^, as well as country-specific, age-specific HBV-related death rates (deaths per 100,000) from cirrhosis and HCC sourced from the Global Burden of Disease (GBD) Results Tool website in December 2018 (http://ghdx.healthdata.org/gbd-results-tool). Disability weights for calculating DALYs were obtained from Vos et al.^48^.

### The HBV model

We used a population-level, deterministic, dynamic transmission model^26,49^, resolved according to sex and age, to determine the impact of scaling up timely HepB-BD coverage and the impact of the COVID-19 pandemic on HBV disease burden. In the model, HBV is either transmitted vertically from infected mothers to their new-borns or horizontally from infected members of the population. The horizontal force of infection is determined by the proportion of infectious individuals in the population that are HBeAg+ and HBeAg-, as well as by the risk of transmission amongst young children, which is calibrated for each country. The risk of vertical transmission is also calibrated for each country. The probability of an acute horizontal infection becoming chronic is a function of age, falling from over 53% in one-year-olds to less than 5% in adults over 30 years of age, while the probability of a vertical infection becoming chronic is 88.5%. Individuals who do not develop chronic HBV clear the virus and become immune to HBV. The timely HepB-BD (efficacy of 95% if the mother is HBsAg+ HBeAg-, and 83% if the mother is HBsAg+ HBeAg+) is administered to new-borns, and the administration of HepB3 (efficacy of 95%) is completed at six months of age.

### Model fitting

The model was fit to data from each country using the Approximate Bayesian Computation Sequential Monte Carlo algorithm^50^, and 200 particles were sampled from the posterior distribution of each country (Supplementary Note 3). Vaccine efficacy was varied amongst the 200 particles, with HepB3 efficacy varying between 90% and 100%^51,52^ and the timely HepB-BD efficacy in the case of HBeAg+ mothers varying between 70% and 95%^53–57^. Model results from the 200 particle runs were summarized as the mean or median, with the 2.5 and 97.5 percentiles used to construct 95% credibility intervals. Treatment data and modelling are outlined in Supplementary Note 4.

### Model analysis

Four scenarios were run in the countries (Table 1), all of which involve HepB3 coverage maintained at its 2019 level until 2100 in the country but differ in the levels of timely HepB-BD coverage imposed. The four scenarios are as follows: timely HepB-BD coverage is maintained at its 2019 level until 2100 (status quo HepB3 & HepB-BD scenario), timely HepB-BD coverage is scaled up from its 2019 level to a target level e.g. 90%, between 2020 and 2030 (HepB-BD scale-up scenario), timely HepB-BD coverage drops by a specified percentage (5%, 10%, 15%, or 20%) during 2020, before reverting to its 2019 level from 2021 onwards (HepB-BD disruptions scenario), and timely HepB-BD coverage is maintained at its 2019 level for a few years before being scaled-up to 90% at various rates (delayed HepB-BD scale-up scenario). Analyses were performed by running these scenarios until the year 2100. Analyses were performed for each country-level model and then added up within WHO regions (regional estimates) as well as across WHO regions (global estimates), with 95% credibility intervals calculated for the summed model results under the assumption of independence between the individual country results. Model fitting and analyses were conducted in MATLAB version R2020b^58^ and maps were drawn with the function ggplot2 version 3.3.5^59^ in R version 4.1.1 (2021-08-10) using a shape file from the R package rnaturalearth version 0.1.0^60^.

### Reporting summary

Further information on research design is available in the Nature Research Reporting Summary linked to this article.

## Supplementary information

Supplementary Information

Reporting Summary

## Data Availability

The data sets for running the scripts to generate the results are available at Zenodo (10.5281/zenodo.5177261). This includes demographic data from the United Nations’ 2019 World Population Prospects (Age-specific Fertility Rates, Sex Ratio at Birth, Annual Population by Age – Female, Annual Population by Age – Male, Net Migration Rate, Percentage of Female Deaths by Broad Age Groups, and Percentage of Male Deaths by Broad Age Groups at https://population.un.org/wpp/Download/Standard/, accessed in October 2019), historical vaccination coverage from WHO/UNICEF Estimates of National Immunization Coverage (WUENIC) released in July 2020 (WHO-UNICEF estimates of HepB3 coverage at https://apps.who.int/immunization_monitoring/globalsummary/timeseries/tswucoveragehepb3.html and WHO-UNICEF estimates of HepB_BD coverage at https://apps.who.int/immunization_monitoring/globalsummary/timeseries/tswucoveragehepb_bd.html, accessed in July 2020), and HBsAg prevalence data from the WHO HBsAg dashboard (Hepatitis b surface antigen estimates and number of carriers in 2015 in the general population and Hepatitis b surface antigen estimates and number of carriers in 2015 in the under 5 years of age, currently available at http://situatedlaboratories.net/who-hepB-dashboard/src/#global-strategies as of September 2021). Also included are HepB3 and timely HepB-BD vaccination coverage datasets and HBsAg prevalence datasets from Cui et al.^45^ for China, country-specific HBsAg prevalence data from Razavi-Shearer et al.^32^ and disability weights from Vos et al.^48^.

## Code availability

MATLAB scripts for running the analyses and generating the results are available at Zenodo (10.5281/zenodo.5177261).
