# Supplementary material for: The impact of the timely birth dose vaccine on the global elimination of hepatitis B
Source: Nat Commun. 2021 Oct 28;12:6223. doi: 10.1038/s41467-021-26475-6 (PMC8553835; doi:10.1038/s41467-021-26475-6)
Supplement: Supplementary file 1 — Supplementary Information [file 41467_2021_26475_MOESM1_ESM.pdf]

# Supplementary Information: The impact of the timely birth-dose vaccine on the global elimination of hepatitis B

Margaret J. de Villiers, Shevanthi Nayagam and Timothy B. Hallett

## Supplementary Note 1: Countries included in the modelling

The 110 low and middle-income countries modelled (Supplementary Fig. 1) together represent all six WHO regions and include all current GAVI-eligible countries. All low-income and lower-middle income countries are included in the modelling (https://datahelpdesk.worldbank.org/knowledgebase/articles/906519-world-bank-country-and-lending-groups, accessed in December 2020), and the 110 modelled countries together contain 92% of global HBsAg prevalent cases (Supplementary Table 2; http://whohbsagdashboard.com/#hbv-country-profiles, accessed in January 2021, currently available at http://situatedlaboratories.net/who-hepB-dashboard/src/#global-strategies as of September 2021). Within all six WHO regions, the modelled countries together contain a higher HBsAg prevalence than do the excluded countries. However, EURO and PAHO each contain several high-income countries (HICs). Even though these HICs tend to have low HBsAg prevalences, their combined population sizes within each

region results in the HICs containing over 50% of HBsAg prevalent cases in EURO and PAHO, making model outputs for these two WHO regions less complete than for AFRO, EMRO, SEARO and WPRO.

# Supplementary Note 2: The HBV model

The model[1,2] is partitioned into acute states and chronic states, with the acute part containing the Severe Acute and Non-severe acute states, and the chronic part containing the states Immune Tolerant, Immune Reactive, Asymptomatic Carrier, HBeAg negative Chronic HBV, Compensated Cirrhosis, Decompensated Cirrhosis and Liver Cancer. Individuals in the Immune Tolerant and the Immune Reactive states are HBsAg+ HBeAg+ and are considered to be 15 times more infectious for horizontal transmission than the individuals in the other disease states, who are HBsAg+ HBeAg-. The Immune Reactive, HBeAg negative Chronic HBV, Compensated Cirrhosis and Decompensated Cirrhosis states constitute the population of HBV-infected individuals considered eligible for antiviral treatment. HBV-related deaths can occur from the Severe Acute, Compensated Cirrhosis, Decompensated Cirrhosis and Liver Cancer states.

# Supplementary Note 3: Model fitting

A weighted mean absolute percentage error objective function was used, with the fit of the model to younger age groups in the data from the literature considered more important than the fit to older age groups, since the younger age groups constitute the population group that informs the transmission rates. The most important terms were given the highest weightings (the fit of younger age groups for HBsAg prevalence and cirrhosis and cancer death rates),

the less important terms intermediate weightings (the fit of older age groups for HBsAg prevalence and death rates), and the least important term the lowest weighting (the fit of HBeAg/HBsAg prevalence in women of childbearing age). The fits to the prevalence data and the fits to the death rates data were given equal weighting in the objective function. The seven model parameters were calibrated for each country, including the risk of horizontal transmission to susceptible 1 to 4 year olds, the risk of vertical transmission from HBsAg+, HBeAg- mothers to their infants at birth without intervention, the rate at which seroconversion from HBsAg+ HBeAg+ to HBsAg+ HBeAg- declines with age, the rate at which liver cancer risk increases with age, the rate at which cirrhosis risk increases with age, the ratio of males to females developing liver cancer, and the ratio of males to females developing cirrhosis.

The Approximate Bayesian Computation Sequential Monte Carlo (ABC SMC) algorithm[3] was used to fit the model to data from each country[2], starting with uniform prior distributions for the seven parameters listed above. Successive populations each contained $n$ = 200 particles, with the tolerances of successive populations chosen as the median of the errors of the particles in the previous population of particles. Noise was added to particles sampled from the previous population of particles using a multivariate normal distribution as the perturbation kernel, with zero mean and a covariance matrix calculated from the previous population of particles. The calibration analyses were run until a population of particles was formed with a tolerance level below 0.25.

# Supplementary Note 4: Modelling treatment

Numbers of individuals on antiviral treatment in the countries in 2016 were obtained from Razavi-Shearer et al. (2018)[4], with countries not listed in this source assumed not to have introduced antiviral treatment by 2016. Treatment was initiated in the simulations in the year 2016 in each country, and the rate of treatment-eligible individuals moved to treatment was maintained at a constant rate until 2100, the last year of the simulations in each country. Treatment

levels varied within a country across the 200 particles, ranging from a level that achieves the same treatment coverage amongst treatment-eligible individuals in the years 2016 and 2030 to a level that achieves 40% treatment coverage amongst treatment-eligible individuals in the year 2030. HBsAg+ individuals were considered eligible for antiviral treatment if they are in the Immune Reactive, HBeAg negative chronic HBV, Compensated Cirrhosis or Decompensated Cirrhosis disease states of HBV. The upper level of 40% treatment coverage amongst treatment-eligible individuals in 2030 was considered a reasonable upper limit, since most countries currently have very low levels of treatment[5] and are therefore unlikely to reach the WHO goal of 80% coverage amongst treatment-eligible individuals by 2030[6]. The results of these analyses are mostly shown in the main text of the manuscript, whereas the results of sensitivity analyses involving higher levels of treatment are provided in Supplementary Tables 3–6.

# Supplementary Figures

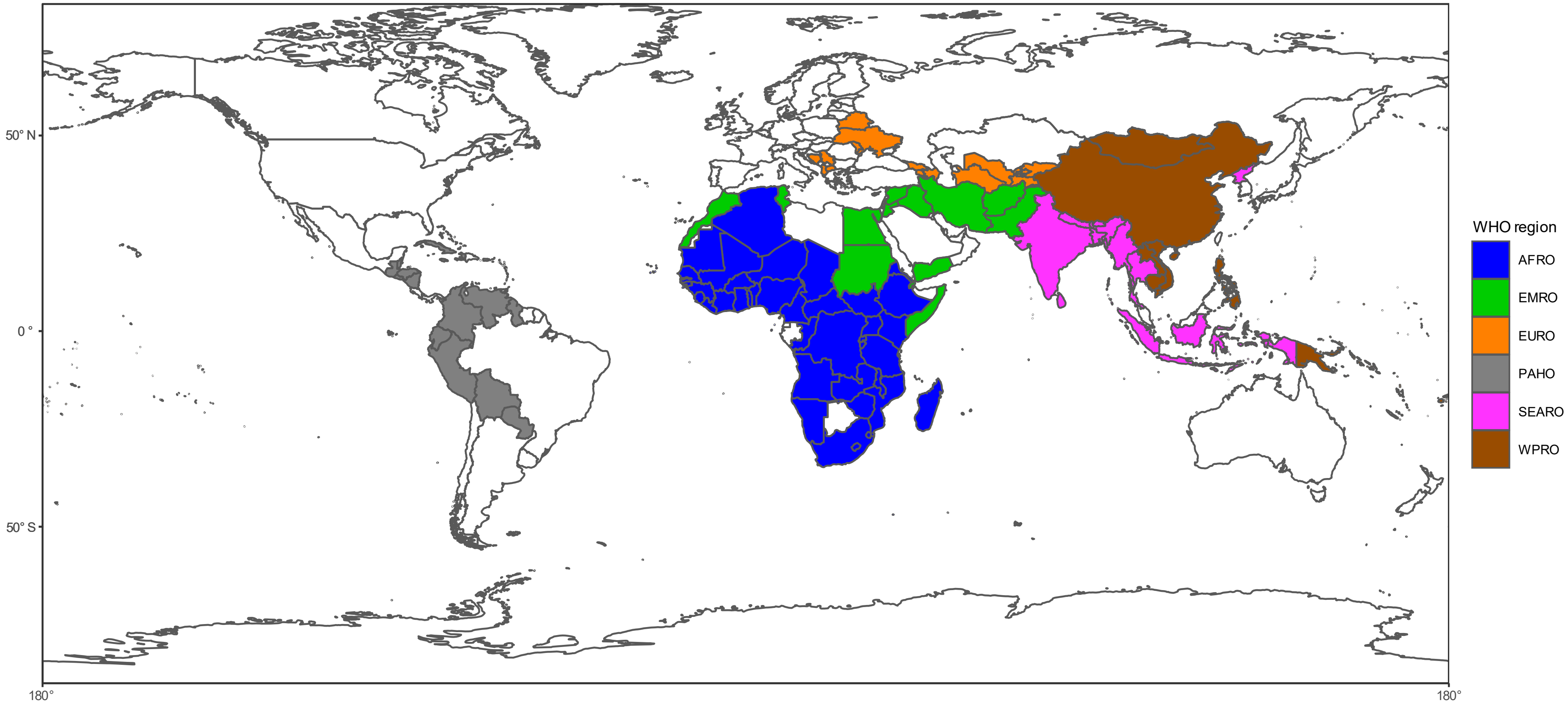


**Supplementary Fig. 1 Map of the 110 low to middle income countries included in the study and their assignment to each of the six WHO regions[7].** WHO: World Health Organization.

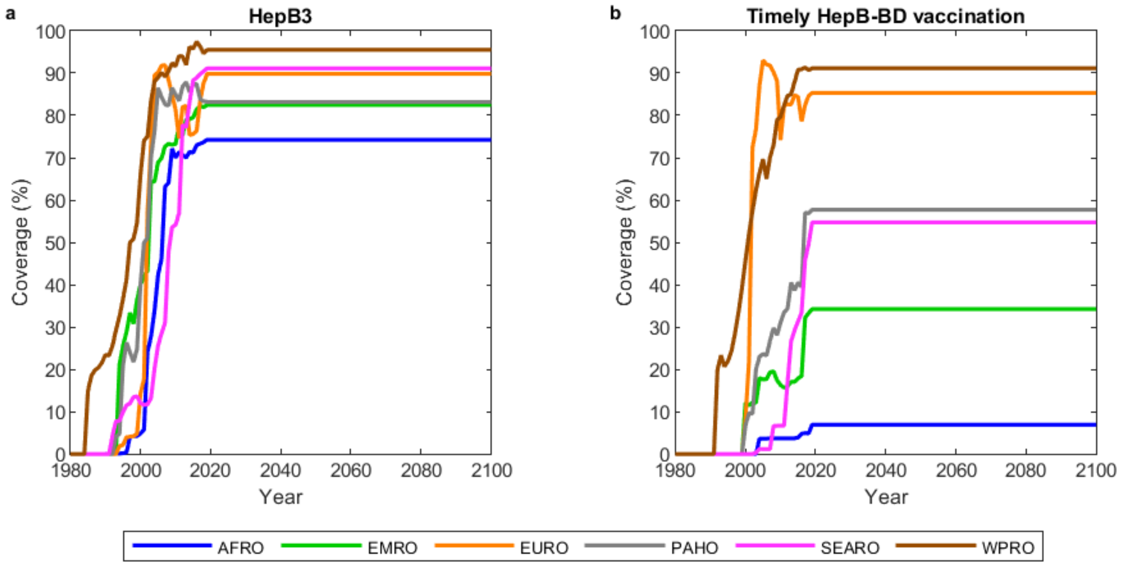


**Supplementary Fig. 2 Vaccination coverage for the six WHO regions in the status quo HepB3 & HepB-BD scenario. a** Overall HepB3 coverage and **b** overall timely HepB-BD vaccination coverage in the status quo HepB3 & HepB-BD scenario within the populations of each of the WHO regions. The six WHO regions AFRO, EMRO, EURO, PAHO, SEARO and WPRO are shown in Supplementary Fig. 1. HBV: hepatitis B virus; HepB3: infant HBV vaccine series; timely HepB-BD: timely birth dose; WHO: World Health Organization.

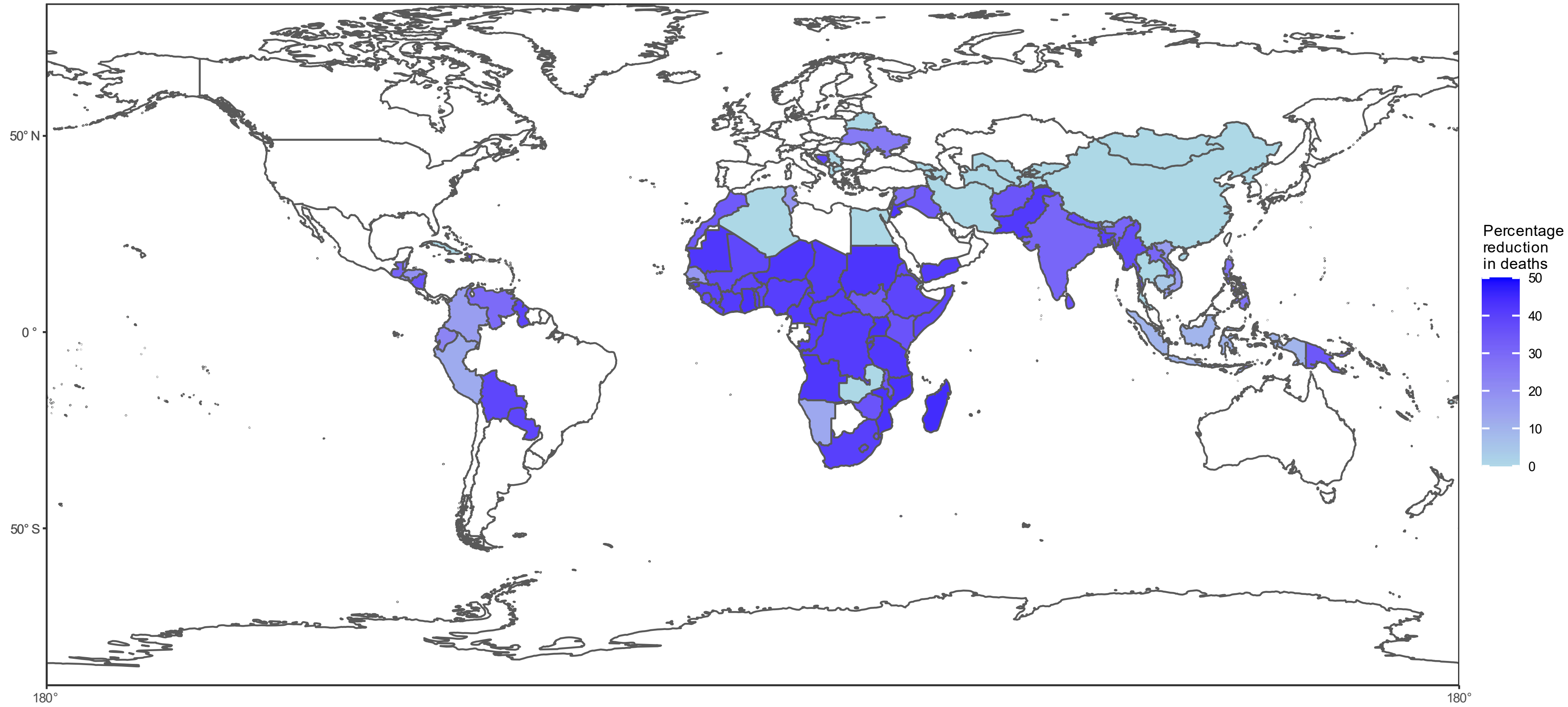


**Supplementary Fig. 3 Percentage reduction of mean HBV-related deaths averted in the 2020 to 2030 birth cohorts in each country if timely HepB-BD coverage is scaled up to ≥90% by 2030 (the HepB-BD scale-up scenario) relative to the status quo HepB3 & HepB-BD scenario.** HBV: hepatitis B virus; HepB3: infant HBV vaccine series; timely HepB-BD: timely birth dose.

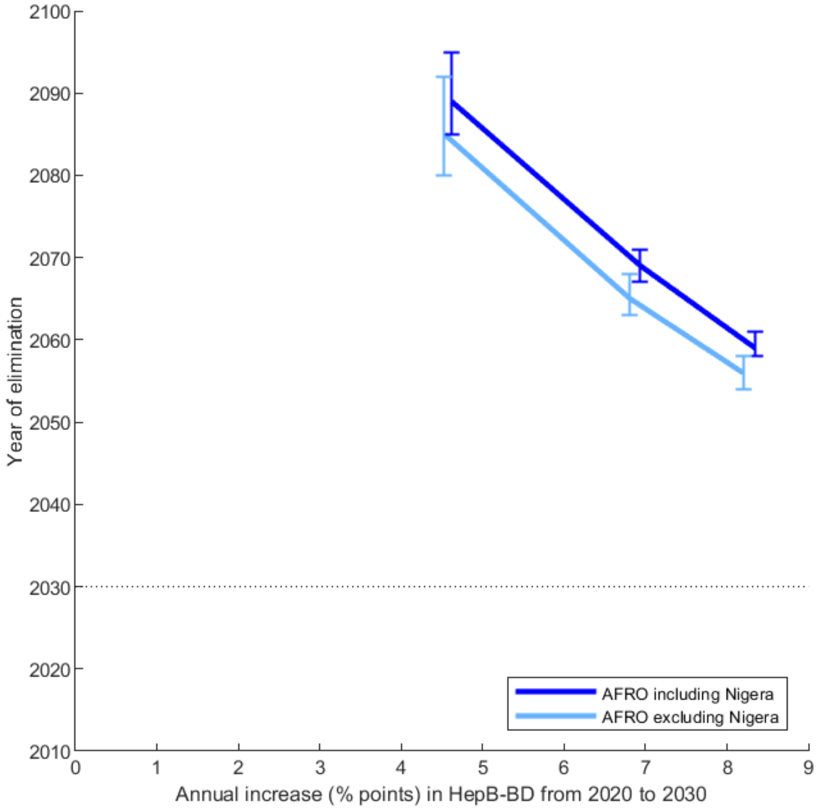


**Supplementary Fig. 4 Year of elimination (median and 95% credibility intervals) for different rates of annual scale-up of timely HepB-BD coverage in the WHO AFRO region, with Nigeria included (dark-blue line) and Nigeria excluded (light-blue line).** At annual increases in timely HepB-BD coverage less than 3%, elimination did not occur before 2100. The dotted line at the year 2030 is for reference purposes only. For each WHO region, number of HBsAg prevalent cases in five-year-olds and total number of five year olds were summed across countries. Number of HBsAg prevalent cases in five-year-olds was divided by total number of five year olds to give HBsAg prevalence in five year olds. The year of elimination was identified as the year in which HBsAg prevalence in five year olds falls below 0.1%. This was repeated $n$ = 200 times using independent draws from the posterior distribution of each country. AFRO: WHO's Africa region; timely HepB-BD: timely birth dose; WHO: World Health Organization.

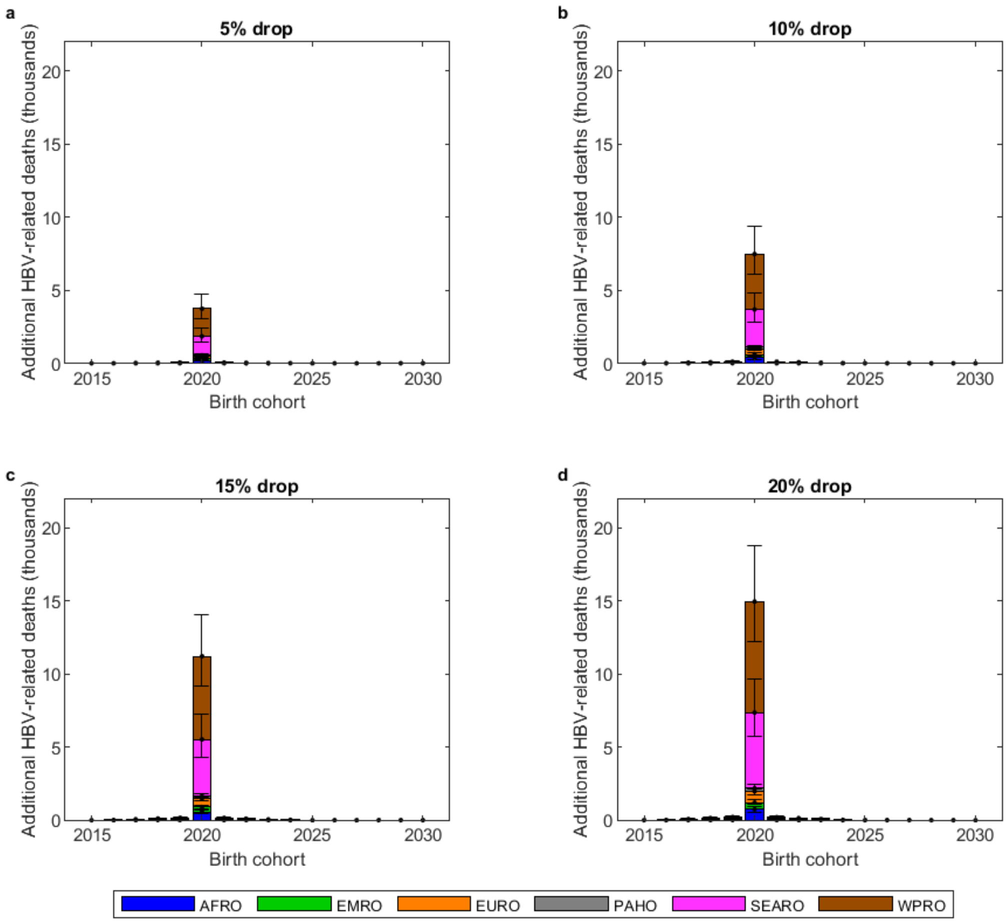


**Supplementary Fig. 5 Additional HBV-related deaths (mean and 95% credibility intervals) in the 2015 to 2030 birth cohorts due to a drop in the percentage of new-borns receiving timely HepB-BD in 2020 (the HepB-BD disruptions scenarios) relative to the status quo HepB3 & HepB-BD scenario.** Results are the sums within WHO regions from all modelled countries of *n* = 200 model outcomes resulting from 200 particles from the posterior distribution of each country. The six WHO regions AFRO, EMRO, EURO, PAHO, SEARO and WPRO are shown in Supplementary Fig. 1. HBV: hepatitis B virus; HepB3: infant HBV vaccine series; timely HepB-BD: timely birth dose; WHO: World Health Organization.

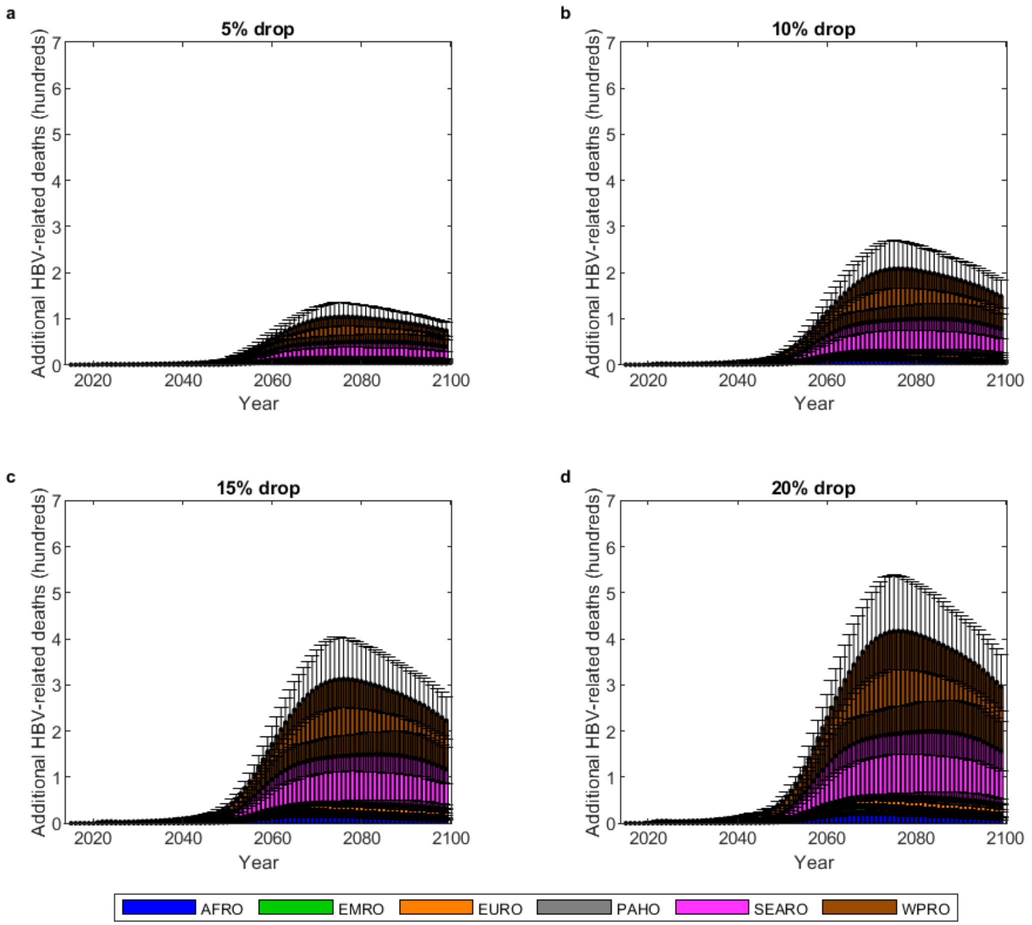


**Supplementary Fig. 6 Additional HBV-related deaths (mean and 95% credibility intervals) in the years 2020 to 2100 due to a drop in the percentage of new-borns receiving timely HepB-BD in 2020 (the HepB-BD disruptions scenarios) relative to the status quo HepB3 & HepB-BD scenario.** Results are the sums within WHO regions from all modelled countries of *n* = 200 model outcomes resulting from 200 particles from the posterior distribution of each country. The six WHO regions AFRO, EMRO, EURO, PAHO, SEARO and WPRO are shown in Supplementary Fig. 1. HBV: hepatitis B virus; HepB3: infant HBV vaccine series; timely HepB-BD: timely birth dose; WHO: World Health Organization.

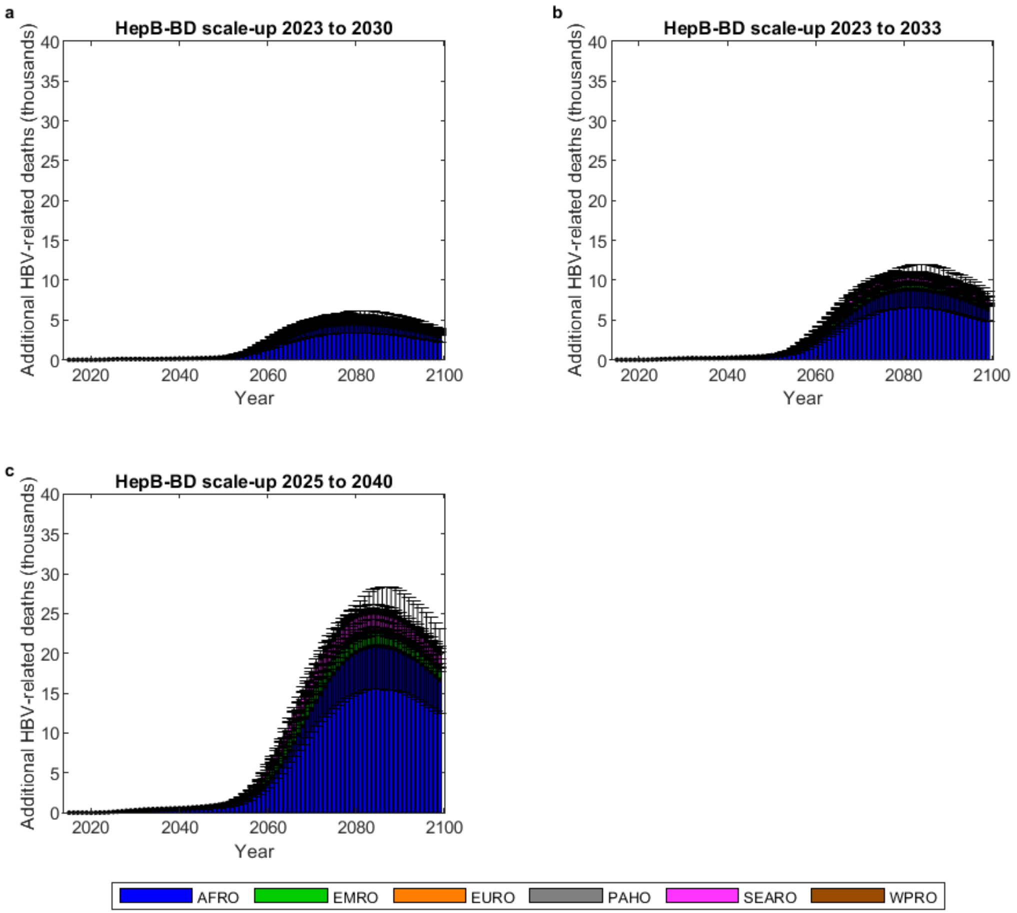


**Supplementary Fig. 7 Additional HBV-related deaths (mean and 95% credibility intervals) in the years 2020 to 2100 due to delays in scaling up timely HepB-BD coverage to ≥90% (the delayed HepB-BD scale-up scenarios) relative to scaling up timely HepB-BD coverage to ≥90% between 2020 and 2030 (the HepB-BD scale-up scenario)**. Results are the sums within WHO regions from all modelled countries of $n$ = 200 model outcomes resulting from 200 particles from the posterior distribution of each country. The six WHO regions AFRO, EMRO, EURO, PAHO, SEARO and WPRO are shown in Supplementary Fig. 1. HBV: hepatitis B virus; timely HepB-BD: timely birth dose; WHO: World Health Organization.

# Supplementary Tables

**Supplementary Table 1** Sources of data used for model calibrations. The six WHO regions AFRO, EMRO, EURO, PAHO, SEARO and WPRO are shown in Supplementary Fig. 1. GBD: Global Burden of Disease; HBeAg: hepatitis B e antigen; HBsAg: hepatitis B surface antigen; HBV: hepatitis B virus; HCC: hepatocellular carcinoma; ISO: International Organization for Standardization; WHO: World Health Organization.

| Country | ISO country code (WHO region) | HBsAg prevalence* | HBeAg/HBsAg prevalence in women of childbearing age** | Death rates (deaths per 100,000) from HBV-related cirrhosis or HCC*** |
|---|---|---|---|---|
| Afghanistan | AFG (EMRO) | WHO dashboard | Ott et al. (2012)[8,9] | GBD Results Tool |
| Angola | AGO (AFRO) | Razavi-Shearer et al. (2018)[4] | Ott et al. (2012)[8,9] | GBD Results Tool |
| Albania | ALB (EURO) | Razavi-Shearer et al. (2018)[4] | Razavi-Shearer et al. (2018)[4] | GBD Results Tool |
| Armenia | ARM (EURO) | Razavi-Shearer et al. (2018)[4] | Ott et al. (2012)[8,9] | GBD Results Tool |
| Azerbaijan | AZE (EURO) | Razavi-Shearer et al. (2018)[4] | Ott et al. (2012)[8,9] | GBD Results Tool |
| Burundi | BDI (AFRO) | Razavi-Shearer et al. (2018)[4] | Razavi-Shearer et al. (2018)[4] | GBD Results Tool |
| Benin | BEN (AFRO) | WHO dashboard | Ott et al. (2012)[8,9] | GBD Results Tool |
| Burkina Faso | BFA (AFRO) | Razavi-Shearer et al. (2018)[4] | Razavi-Shearer et al. (2018)[4] | GBD Results Tool |
| Bangladesh | BGD (SEARO) | Razavi-Shearer et al. (2018)[4] | Razavi-Shearer et al. (2018)[4] | GBD Results Tool |
| Bosnia and Herzegovina | BIH (EURO) | WHO dashboard | Ott et al. (2012)[8,9] | GBD Results Tool |
| Belarus | BLR (EURO) | Razavi-Shearer et al. (2018)[4] | Ott et al. (2012)[8,9] | GBD Results Tool |
| Belize | BLZ (PAHO) | Razavi-Shearer et al. (2018)[4] | Ott et al. (2012)[8,9] | GBD Results Tool |
| Bolivia | BOL (PAHO) | WHO dashboard | Ott et al. (2012)[8,9] | GBD Results Tool |
| Bhutan | BTN (SEARO) | WHO dashboard | Ott et al. (2012)[8,9] | GBD Results Tool |
| Central African Republic | CAF (AFRO) | Razavi-Shearer et al. (2018)[4] | Ott et al. (2012)[8,9] | GBD Results Tool |
| China | CHN (WPRO) | Cui et al. (2017)[10] | Razavi-Shearer et al. (2018)[4] | GBD Results Tool |
| Ivory Coast | CIV (AFRO) | Razavi-Shearer et al. (2018)[4] | Razavi-Shearer et al. (2018)[4] | GBD Results Tool |
| Cameroon | CMR (AFRO) | Razavi-Shearer et al. (2018)[4] | Razavi-Shearer et al. (2018)[4] | GBD Results Tool |
| Democratic Republic of the Congo | COD (AFRO) | WHO dashboard | Ott et al. (2012)[8,9] | GBD Results Tool |
| Congo | COG (AFRO) | WHO dashboard | Ott et al. (2012)[8,9] | GBD Results Tool |
| Colombia | COL (PAHO) | Razavi-Shearer et al. (2018)[4] | Ott et al. (2012)[8,9] | GBD Results Tool |
| Comoros | COM (AFRO) | WHO dashboard | Ott et al. (2012)[8,9] | GBD Results Tool |
| Cape Verde | CPV (AFRO) | WHO dashboard | Ott et al. (2012)[8,9] | GBD Results Tool |
| Cuba | CUB (PAHO) | Razavi-Shearer et al. (2018)[4] | Ott et al. (2012)[8,9] | GBD Results Tool |
| Djibouti | DJI (EMRO) | WHO dashboard | Ott et al. (2012)[8,9] | GBD Results Tool |
| Algeria | DZA (AFRO) | Razavi-Shearer et al. (2018)[4] | Ott et al. (2012)[8,9] | GBD Results Tool |
| Ecuador | ECU (PAHO) | WHO dashboard | Ott et al. (2012)[8,9] | GBD Results Tool |
| Egypt | EGY (EMRO) | Razavi-Shearer et al. (2018)[4] | Razavi-Shearer et al. (2018)[4] | GBD Results Tool |

| Country | ISO country code (WHO region) | HBsAg prevalence* | HBeAg/HBsAg prevalence in women of childbearing age** | Death rates (deaths per 100,000) from HBV-related cirrhosis or HCC*** |
|---|---|---|---|---|
| Eritrea | ERI (AFRO) | WHO dashboard | Ott et al. (2012)[8,9] | GBD Results Tool |
| Ethiopia | ETH (AFRO) | Razavi-Shearer et al. (2018)[4] | Razavi-Shearer et al. (2018)[4] | GBD Results Tool |
| Fiji | FJI (WPRO) | Razavi-Shearer et al. (2018)[4] | Razavi-Shearer et al. (2018)[4] | GBD Results Tool |
| Micronesia | FSM (WPRO) | WHO dashboard | Ott et al. (2012)[8,9] | GBD Results Tool |
| Georgia | GEO (EURO) | Razavi-Shearer et al. (2018)[4] | Ott et al. (2012)[8,9] | GBD Results Tool |
| Ghana | GHA (AFRO) | Razavi-Shearer et al. (2018)[4] | Razavi-Shearer et al. (2018)[4] | GBD Results Tool |
| Guinea | GIN (AFRO) | WHO dashboard | Ott et al. (2012)[8,9] | GBD Results Tool |
| Gambia | GMB (AFRO) | Razavi-Shearer et al. (2018)[4] | Razavi-Shearer et al. (2018)[4] | GBD Results Tool |
| Guinea-Bissau | GNB (AFRO) | WHO dashboard | Ott et al. (2012)[8,9] | GBD Results Tool |
| Guatemala | GTM (PAHO) | Razavi-Shearer et al. (2018)[4] | Ott et al. (2012)[8,9] | GBD Results Tool |
| Guyana | GUY (PAHO) | WHO dashboard | Ott et al. (2012)[8,9] | GBD Results Tool |
| Honduras | HND (PAHO) | WHO dashboard | Ott et al. (2012)[8,9] | GBD Results Tool |
| Haiti | HTI (PAHO) | Razavi-Shearer et al. (2018)[4] | Ott et al. (2012)[8,9] | GBD Results Tool |
| Indonesia | IDN (SEARO) | Razavi-Shearer et al. (2018)[4] | Razavi-Shearer et al. (2018)[4] | GBD Results Tool |
| India | IND (SEARO) | Razavi-Shearer et al. (2018)[4] | Razavi-Shearer et al. (2018)[4] | GBD Results Tool |
| Iran | IRN (EMRO) | Razavi-Shearer et al. (2018)[4] | Razavi-Shearer et al. (2018)[4] | GBD Results Tool |
| Iraq | IRQ (EMRO) | Razavi-Shearer et al. (2018)[4] | Ott et al. (2012)[8,9] | GBD Results Tool |
| Jamaica | JAM (PAHO) | Razavi-Shearer et al. (2018)[4] | Ott et al. (2012)[8,9] | GBD Results Tool |
| Jordan | JOR (EMRO) | Razavi-Shearer et al. (2018)[4] | Razavi-Shearer et al. (2018)[4] | GBD Results Tool |
| Kenya | KEN (AFRO) | Razavi-Shearer et al. (2018)[4] | Razavi-Shearer et al. (2018)[4] | GBD Results Tool |
| Kyrgyzstan | KGZ (EURO) | Razavi-Shearer et al. (2018)[4] | Razavi-Shearer et al. (2018)[4] | GBD Results Tool |
| Cambodia | KHM (WPRO) | Razavi-Shearer et al. (2018)[4] | Ott et al. (2012)[8,9] | GBD Results Tool |
| Kiribati | KIR (WPRO) | Razavi-Shearer et al. (2018)[4] | Razavi-Shearer et al. (2018)[4] | GBD Results Tool |
| Laos | LAO (WPRO) | Razavi-Shearer et al. (2018)[4] | Ott et al. (2012)[8,9] | GBD Results Tool |
| Liberia | LBR (AFRO) | WHO dashboard | Ott et al. (2012)[8,9] | GBD Results Tool |
| Sri Lanka | LKA (SEARO) | WHO dashboard | Ott et al. (2012)[8,9] | GBD Results Tool |
| Lesotho | LSO (AFRO) | WHO dashboard | Ott et al. (2012)[8,9] | GBD Results Tool |
| Morocco | MAR (EMRO) | Razavi-Shearer et al. (2018)[4] | Razavi-Shearer et al. (2018)[4] | GBD Results Tool |
| Moldova | MDA (EURO) | WHO dashboard | Ott et al. (2012)[8,9] | GBD Results Tool |
| Madagascar | MDG (AFRO) | Razavi-Shearer et al. (2018)[4] | Razavi-Shearer et al. (2018)[4] | GBD Results Tool |
| Marshall Islands | MHL (WPRO) | WHO dashboard | Ott et al. (2012)[8,9] | GBD Results Tool |
| North Macedonia | MKD (EURO) | WHO dashboard | Ott et al. (2012)[8,9] | GBD Results Tool |
| Mali | MLI (AFRO) | Razavi-Shearer et al. (2018)[4] | Razavi-Shearer et al. (2018)[4] | GBD Results Tool |
| Myanmar | MMR (SEARO) | Razavi-Shearer et al. (2018)[4] | Ott et al. (2012)[8,9] | GBD Results Tool |
| Mongolia | MNG (WPRO) | Razavi-Shearer et al. (2018)[4] | Ott et al. (2012)[8,9] | GBD Results Tool |
| Mozambique | MOZ (AFRO) | Razavi-Shearer et al. (2018)[4] | Ott et al. (2012)[8,9] | GBD Results Tool |
| Mauritania | MRT (AFRO) | Razavi-Shearer et al. (2018)[4] | Ott et al. (2012)[8,9] | GBD Results Tool |

| Country | ISO country code (WHO region) | HBsAg prevalence* | HBeAg/HBsAg prevalence in women of childbearing age** | Death rates (deaths per 100,000) from HBV-related cirrhosis or HCC*** |
|---|---|---|---|---|
| Malawi | MWI (AFRO) | Razavi-Shearer et al. (2018)[4] | Ott et al. (2012)[8,9] | GBD Results Tool |
| Namibia | NAM (AFRO) | WHO dashboard | Ott et al. (2012)[8,9] | GBD Results Tool |
| Niger | NER (AFRO) | WHO dashboard | Ott et al. (2012)[8,9] | GBD Results Tool |
| Nigeria | NGA (AFRO) | Razavi-Shearer et al. (2018)[4] | Razavi-Shearer et al. (2018)[4] | GBD Results Tool |
| Nicaragua | NIC (PAHO) | Razavi-Shearer et al. (2018)[4] | Ott et al. (2012)[8,9] | GBD Results Tool |
| Nepal | NPL (SEARO) | WHO dashboard | Ott et al. (2012)[8,9] | GBD Results Tool |
| Pakistan | PAK (EMRO) | Razavi-Shearer et al. (2018)[4] | Razavi-Shearer et al. (2018)[4] | GBD Results Tool |
| Peru | PER (PAHO) | Razavi-Shearer et al. (2018)[4] | Ott et al. (2012)[8,9] | GBD Results Tool |
| Philippines | PHL (WPRO) | Razavi-Shearer et al. (2018)[4] | Razavi-Shearer et al. (2018)[4] | GBD Results Tool |
| Papua New Guinea | PNG (WPRO) | Razavi-Shearer et al. (2018)[4] | Razavi-Shearer et al. (2018)[4] | GBD Results Tool |
| Korea, North | PRK (SEARO) | WHO dashboard | Ott et al. (2012)[8,9] | GBD Results Tool |
| Paraguay | PRY (PAHO) | WHO dashboard | Ott et al. (2012)[8,9] | GBD Results Tool |
| Rwanda | RWA (AFRO) | Razavi-Shearer et al. (2018)[4] | Ott et al. (2012)[8,9] | GBD Results Tool |
| Sudan | SDN (EMRO) | Razavi-Shearer et al. (2018)[4] | Ott et al. (2012)[8,9] | GBD Results Tool |
| Senegal | SEN (AFRO) | Razavi-Shearer et al. (2018)[4] | Razavi-Shearer et al. (2018)[4] | GBD Results Tool |
| Solomon Islands | SLB (WPRO) | WHO dashboard | Ott et al. (2012)[8,9] | GBD Results Tool |
| Sierra Leone | SLE (AFRO) | WHO dashboard | Ott et al. (2012)[8,9] | GBD Results Tool |
| El Salvador | SLV (PAHO) | Razavi-Shearer et al. (2018)[4] | Ott et al. (2012)[8,9] | GBD Results Tool |
| Somalia | SOM (EMRO) | WHO dashboard | Ott et al. (2012)[8,9] | GBD Results Tool |
| Serbia | SRB (EURO) | WHO dashboard | Ott et al. (2012)[8,9] | GBD Results Tool |
| South Sudan | SSD (AFRO) | WHO dashboard | Ott et al. (2012)[8,9] | GBD Results Tool |
| Sao Tome and Principe | STP (AFRO) | WHO dashboard | Ott et al. (2012)[8,9] | GBD Results Tool |
| Eswatini | SWZ (AFRO) | WHO dashboard | Ott et al. (2012)[8,9] | GBD Results Tool |
| Syria | SYR (EMRO) | Razavi-Shearer et al. (2018)[4] | Ott et al. (2012)[8,9] | GBD Results Tool |
| Chad | TCD (AFRO) | Razavi-Shearer et al. (2018)[4] | Ott et al. (2012)[8,9] | GBD Results Tool |
| Togo | TGO (AFRO) | WHO dashboard | Ott et al. (2012)[8,9] | GBD Results Tool |
| Thailand | THA (SEARO) | Razavi-Shearer et al. (2018)[4] | Razavi-Shearer et al. (2018)[4] | GBD Results Tool |
| Tajikistan | TJK (EURO) | Razavi-Shearer et al. (2018)[4] | Ott et al. (2012)[8,9] | GBD Results Tool |
| Turkmenistan | TKM (EURO) | Razavi-Shearer et al. (2018)[4] | Ott et al. (2012)[8,9] | GBD Results Tool |
| Timor-Leste | TLS (SEARO) | WHO dashboard | Ott et al. (2012)[8,9] | GBD Results Tool |
| Tonga | TON (WPRO) | WHO dashboard | Ott et al. (2012)[8,9] | GBD Results Tool |
| Tunisia | TUN (EMRO) | Razavi-Shearer et al. (2018)[4] | Razavi-Shearer et al. (2018)[4] | GBD Results Tool |
| Tuvalu | TUV (WPRO) | WHO dashboard | Ott et al. (2012)[8,9] | GBD Results Tool |
| Tanzania | TZA (AFRO) | Razavi-Shearer et al. (2018)[4] | Ott et al. (2012)[8,9] | GBD Results Tool |
| Uganda | UGA (AFRO) | Razavi-Shearer et al. (2018)[4] | Razavi-Shearer et al. (2018)[4] | GBD Results Tool |
| Ukraine | UKR (EURO) | WHO dashboard | Ott et al. (2012)[8,9] | GBD Results Tool |
| Uzbekistan | UZB (EURO) | Razavi-Shearer et al. (2018)[4] | Razavi-Shearer et al. (2018)[4] | GBD Results Tool |

| Country | ISO country code (WHO region) | HBsAg prevalence* | HBeAg/HBsAg prevalence in women of childbearing age** | Death rates (deaths per 100,000) from HBV-related cirrhosis or HCC*** |
|---|---|---|---|---|
| Venezuela | VEN (PAHO) | Razavi-Shearer et al. (2018)[4] | Ott et al. (2012)[8,9] | GBD Results Tool |
| Viet Nam | VNM (WPRO) | Razavi-Shearer et al. (2018)[4] | Razavi-Shearer et al. (2018)[4] | GBD Results Tool |
| Vanuatu | VUT (WPRO) | WHO dashboard | Ott et al. (2012)[8,9] | GBD Results Tool |
| Samoa | WSM (WPRO) | WHO dashboard | Ott et al. (2012)[8,9] | GBD Results Tool |
| Yemen | YEM (EMRO) | Razavi-Shearer et al. (2018)[4] | Ott et al. (2012)[8,9] | GBD Results Tool |
| South Africa | ZAF (AFRO) | WHO dashboard | Ott et al. (2012)[8,9] | GBD Results Tool |
| Zambia | ZMB (AFRO) | Razavi-Shearer et al. (2018)[4] | Razavi-Shearer et al. (2018)[4] | GBD Results Tool |
| Zimbabwe | ZWE (AFRO) | Razavi-Shearer et al. (2018)[4] | Razavi-Shearer et al. (2018)[4] | GBD Results Tool |

*HBsAg prevalence data from Razavi-Shearer et al. (2018)[4] are for five-year-olds and for all ages in 2016; HBsAg prevalence data from the WHO dashboard (http://whohbsagdashboard.com/#hbv-country-profiles, accessed in October 2019, currently available at http://situatedlaboratories.net/who-hepB-dashboard/src/#global-strategies as of September 2021) are for under-five-year-olds and for all ages in 2015; HBsAg prevalence data from Cui et al. (2017)[10] are for under-five-year-olds and for all ages in 1992, 2006 and 2014. Note that for countries with an HBsAg prevalence of <0.1% in Razavi-Shearer et al. (2018)[4], we fit to an HBsAg prevalence of 0.05%. Where possible, we used HBsAg prevalence data from Razavi-Shearer et al. (2018)[4] (this is the most up-to-date published source for most countries). Where unavailable, we instead used estimates from the WHO dashboard (http://whohbsagdashboard.com/#hbv-country-profiles, accessed in October 2019, currently available at http://situatedlaboratories.net/who-hepB-dashboard/src/#global-strategies as of September 2021). The single exception is for China, where multiple surveys are available furnishing estimates of prevalence at multiple time-points (Cui et al. 2017[10]).

**HBeAg prevalence amongst HBsAg+ women of childbearing age from Ott et al. (2012)[8,9] are for 2005; HBeAg prevalence amongst HBsAg+ women of childbearing age from Razavi-Shearer et al. (2018)[4] are for a variety of different years. Where possible, we used HBeAg prevalence data from Razavi-Shearer et al. (2018)[4] (this is the most up-to-date published source for most countries). Where unavailable, we instead used the regional average data reported in Ott et al. (2012)[8,9], weighting prevalences in the more fertile age groups more heavily than in less fertile age groups (per the population size and fertility data from the United Nations' 2019 World Population Prospects (https://population.un.org/wpp/Download/Standard/Population/, accessed in October 2019).

***The HBV-related death rates due to cirrhosis and HCC from the GBD Results Tool website (http://ghdx.healthdata.org/gbd-results-tool, accessed in December 2018) used were for those under-40-year-olds and those over-40-year-olds in 1990 and 2005.

**Supplementary Table 2** HBsAg prevalence (http://whohbsagdashboard.com/#hbv-country-profiles, accessed in January 2021, currently available at http://situatedlaboratories.net/who-hepB-dashboard/src/#global-strategies as of September 2021) in all age groups in 2015 in the countries modelled in the WHO regions. The six WHO regions AFRO, EMRO, EURO, PAHO, SEARO and WPRO are shown in Supplementary Fig. 1. HBsAg: hepatitis B surface antigen; WHO: World Health Organization.

| WHO region | HBsAg prevalence in modelled countries (%) | HBsAg prevalence in excluded countries (%) | HBsAg prevalent cases located in modelled countries (%) |
|---|---|---|---|
| AFRO | 6.10 | 4.40 | 99.52 |
| EMRO | 3.41 | 1.68 | 94.81 |
| EURO | 2.75 | 1.39 | 26.81 |
| PAHO | 1.51 | 0.46 | 46.90 |
| SEARO | 2.05 | 1.10 | 99.99 |
| WPRO | 6.71 | 1.97 | 95.81 |
| Global | 4.31 | 1.10 | 92.02 |

**Supplementary Table 3** Mean number of HBV-related deaths averted regionally and globally in birth cohorts born between 2020 and 2030 in selected scenarios relative to the status quo HepB3 & HepB-BD scenario, with 95% credibility intervals in parentheses. Two target values are presented for the HepB-BD scale-up scenario i.e. timely HepB-BD expansion to ≥25% and ≥90% by 2030. The six WHO regions AFRO, EMRO, EURO, PAHO, SEARO and WPRO are shown in Supplementary Fig. 1. HBV: hepatitis B virus; HepB3: infant HBV vaccine series; timely HepB-BD: timely birth dose; WHO: World Health Organization.

| WHO region | HepB-BD scale-up (≥25%) | HepB-BD scale-up (≥90%) | HepB-BD disruptions (5%) | HepB-BD disruptions (20%) | delayed HepB-BD scale-up (2023 to 2030) | delayed HepB-BD scale-up (2025 to 2040) |
|---|---|---|---|---|---|---|
| Default settings | | | | | | |
| AFRO | 151,494 (119,519 to 193,142) | 554,318 (438,263 to 704,048) | -189 (-239 to -143) | -758 (-958 to -574) | 402,659 (317,374 to 514,354) | 105,616 (82,944 to 134,661) |
| EMRO | 15,287 (10,640 to 20,937) | 60,970 (44,369 to 81,588) | -121 (-168 to -83) | -482 (-671 to -332) | 44,169 (31,670 to 59,601) | 11,604 (8,227 to 15,747) |
| EURO | 14 (7 to 23) | 534 (277 to 929) | -200 (-321 to -143) | -799 (-1,283 to -571) | 361 (185 to 629) | 90 (46 to 156) |
| PAHO | 653 (421 to 1,042) | 3,538 (2,665 to 4,824) | -51 (-64 to -40) | -204 (-254 to -161) | 2,502 (1,870 to 3,447) | 647 (480 to 903) |
| SEARO | 6,638 (4,471 to 10,628) | 78,360 (51,355 to 109,840) | -1,351 (-1,952 to -921) | -5,406 (-7,811 to -3,683) | 55,702 (36,212 to 78,435) | 14,265 (9,223 to 20,157) |
| WPRO | 0 (0 to 0) | 12,011 (8,775 to 16,402) | -1,942 (-2,923 to -1,258) | -7,769 (-11,693 to -5,034) | 8,293 (6,033 to 11,339) | 2,112 (1,521 to 2,908) |

| WHO region | HepB-BD scale-up (≥25%) | HepB-BD scale-up (≥90%) | HepB-BD disruptions (5%) | HepB-BD disruptions (20%) | delayed HepB-BD scale-up (2023 to 2030) | delayed HepB-BD scale-up (2025 to 2040) |
|---|---|---|---|---|---|---|
| Global | 174,086 (140,552 to 215,953) | 709,732 (582,513 to 885,156) | -3,854 (-4,991 to -2,988) | -15,417 (-19,967 to -11,954) | 513,686 (420,829 to 641,136) | 134,334 (109,995 to 167,603) |
| HepB3 coverage scaled up to 100% coverage in 2020 | | | | | | |
| AFRO | 125,119 (99,143 to 156,005) | 455,090 (360,024 to 565,464) | -184 (-234 to -140) | -738 (-936 to -559) | 330,694 (261,281 to 412,011) | 85,894 (67,819 to 107,233) |
| EMRO | 13,435 (9,075 to 18,715) | 53,391 (38,131 to 73,082) | -116 (-164 to -79) | -465 (-656 to -315) | 38,621 (27,062 to 53,223) | 10,013 (6,920 to 13,894) |
| EURO | 13 (7 to 22) | 471 (238 to 816) | -195 (-315 to -139) | -782 (-1,262 to -556) | 317 (158 to 554) | 78 (39 to 137) |
| PAHO | 578 (363 to 953) | 3,153 (2,333 to 4,395) | -48 (-60 to -38) | -194 (-242 to -153) | 2,224 (1,628 to 3,134) | 566 (411 to 805) |
| SEARO | 6,484 (4,365 to 10,455) | 74,521 (48,851 to 104,130) | -1,292 (-1,868 to -880) | -5,170 (-7,473 to -3,519) | 52,925 (34,420 to 74,292) | 13,504 (8,737 to 19,060) |
| WPRO | 0 (0 to 0) | 9,258 (6,558 to 13,148) | -1,906 (-2,886 to -1,225) | -7,626 (-11,545 to -4,901) | 6,398 (4,458 to 9,106) | 1,604 (1,106 to 2,285) |
| Global | 145,629 (118,020 to 177,435) | 595,883 (493,540 to 735,745) | -3,743 (-4,856 to -2,887) | -14,974 (-19,430 to -11,551) | 431,179 (355,632 to 533,688) | 111,659 (91,815 to 138,422) |
| Treatment level such that 40% of treatment-eligible individuals are in treatment in 2030 | | | | | | |
| AFRO | 88,124 (73,728 to 98,974) | 322,582 (270,440 to 362,036) | -128 (-144 to -108) | -511 (-578 to -432) | 232,983 (194,904 to 262,598) | 60,838 (50,815 to 68,769) |
| EMRO | 9,780 (7,627 to 11,064) | 38,746 (31,140 to 43,331) | -80 (-96 to -59) | -321 (-384 to -237) | 27,914 (22,085 to 31,431) | 7,303 (5,711 to 8,258) |
| EURO | 8 (6 to 10) | 315 (240 to 412) | -133 (-190 to -109) | -534 (-759 to -437) | 211 (159 to 278) | 52 (39 to 69) |
| PAHO | 388 (309 to 457) | 2,147 (1,810 to 2,435) | -34 (-37 to -30) | -136 (-148 to -120) | 1,505 (1,262 to 1,719) | 387 (322 to 444) |
| SEARO | 4,069 (3,381 to 4,804) | 47,807 (38,826 to 58,176) | -856 (-1,007 to -696) | -3,423 (-4,029 to -2,786) | 33,760 (27,119 to 41,382) | 8,608 (6,847 to 10,613) |
| WPRO | 0 (0 to 0) | 7,633 (6,676 to 8,651) | -1,434 (-2,018 to -960) | -5,737 (-8,072 to -3,842) | 5,234 (4,564 to 5,945) | 1,326 (1,151 to 1,508) |
| Global | 102,369 (88,383 to 114,214) | 419,231 (367,213 to 460,110) | -2,665 (-3,296 to -2,128) | -10,663 (-13,187 to -8,513) | 301,607 (263,766 to 332,036) | 78,513 (68,644 to 86,575) |
| Treatment level such that 80% of treatment-eligible individuals are in treatment in 2030 | | | | | | |
| AFRO | 36,720 (33,270 to 40,493) | 134,171 (121,305 to 147,886) | -51 (-59 to -42) | -204 (-236 to -168) | 95,835 (86,292 to 106,176) | 24,770 (22,245 to 27,547) |
| EMRO | 4,160 (3,224 to 4,918) | 16,195 (12,859 to 19,021) | -33 (-40 to -24) | -132 (-159 to -97) | 11,512 (9,023 to 13,656) | 2,975 (2,313 to 3,554) |
| EURO | 3 (2 to 3) | 116 (78 to 151) | -82 (-112 to -65) | -329 (-449 to -260) | 76 (49 to 100) | 18 (12 to 25) |
| PAHO | 151 (114 to 178) | 833 (673 to 957) | -14 (-16 to -12) | -57 (-63 to -50) | 572 (457 to 661) | 144 (114 to 167) |
| SEARO | 1,341 (1,068 to 1,657) | 16,718 (12,858 to 20,733) | -381 (-437 to -315) | -1,526 (-1,747 to -1,259) | 11,595 (8,806 to 14,536) | 2,924 (2,203 to 3,692) |
| WPRO | 0 (0 to 0) | 3,694 (3,137 to 4,213) | -611 (-904 to -377) | -2,443 (-3,617 to -1,509) | 2,482 (2,084 to 2,854) | 618 (516 to 715) |
| Global | 42,375 (39,060 to 46,161) | 171,727 (159,720 to 184,662) | -1,173 (-1,482 to -921) | -4,691 (-5,927 to -3,685) | 122,070 (113,061 to 131,902) | 31,450 (29,036 to 34,110) |

**Supplementary Table 4** Median year of elimination of HBV (year that HBsAg prevalence in five-year-olds falls below 0.1%) regionally and globally in selected scenarios, with 95% credibility intervals in parentheses. Two target values are presented for the HepB-BD scale-up scenario i.e. timely HepB-BD expansion to ≥25% and ≥90% by 2030. The six WHO regions AFRO, EMRO, EURO, PAHO, SEARO and WPRO are shown in Supplementary Fig. 1. HBsAg: hepatitis B surface antigen; HBV: hepatitis B virus; HepB3: infant HBV vaccine series; timely HepB-BD: timely birth dose; WHO: World Health Organization.

| WHO region | Status quo HepB3 & HepB-BD | HepB-BD scale-up (≥25%) | HepB-BD scale-up (≥90%) | HepB-BD disruptions (5%) | HepB-BD disruptions (20%) | delayed HepB-BD scale-up (2023 to 2030) | delayed HepB-BD scale-up (2025 to 2040) |
|---|---|---|---|---|---|---|---|
| Default settings | | | | | | | |
| AFRO | after 2100 (after 2100 to after 2100) | after 2100 (after 2100 to after 2100) | 2059 (2058 to 2061) | after 2100 (after 2100 to after 2100) | after 2100 (after 2100 to after 2100) | 2060 (2059 to 2062) | 2065 (2063 to 2067) |
| EMRO | after 2100 (after 2100 to after 2100) | after 2100 (2081 to after 2100) | 2047 (2039 to 2055) | after 2100 (after 2100 to after 2100) | after 2100 (after 2100 to after 2100) | 2048 (2040 to 2057) | 2049 (2045 to 2062) |
| EURO | 2037 (2035 to 2038) | 2037 (2035 to 2038) | 2035 (2034 to 2036) | 2037 (2035 to 2038) | 2037 (2035 to 2038) | 2035 (2034 to 2036) | 2036 (2035 to 2037) |
| PAHO | 2042 (2038 to 2048) | 2038 (2035 to 2042) | 2030 (2030 to 2031) | 2042 (2038 to 2048) | 2042 (2038 to 2048) | 2032 (2031 to 2032) | 2035 (2034 to 2036) |
| SEARO | 2053 (2049 to 2061) | 2052 (2048 to 2058) | 2041 (2037 to 2044) | 2053 (2049 to 2061) | 2053 (2049 to 2061) | 2041 (2037 to 2044) | 2043 (2042 to 2044) |
| WPRO | 2045 (2043 to 2047) | 2045 (2043 to 2047) | 2036 (2035 to 2038) | 2045 (2043 to 2047) | 2045 (2043 to 2047) | 2036 (2035 to 2038) | 2040 (2038 to 2041) |
| Global | after 2100 (after 2100 to after 2100) | after 2100 (2093 to after 2100) | 2052 (2050 to 2054) | after 2100 (after 2100 to after 2100) | after 2100 (after 2100 to after 2100) | 2053 (2051 to 2055) | 2055 (2053 to 2058) |
| HepB3 coverage scaled up to 100% coverage in 2020 | | | | | | | |
| AFRO | after 2100 (after 2100 to after 2100) | 2094 (2088 to after 2100) | 2054 (2053 to 2056) | after 2100 (after 2100 to after 2100) | after 2100 (after 2100 to after 2100) | 2055 (2053 to 2057) | 2057 (2055 to 2060) |
| EMRO | 2095 (2068 to after 2100) | 2077 (2059 to after 2100) | 2039 (2035 to 2047) | 2095 (2068 to after 2100) | 2095 (2068 to after 2100) | 2039 (2035 to 2047) | 2044 (2043 to 2047) |
| EURO | 2036 (2035 to 2037) | 2036 (2035 to 2037) | 2035 (2034 to 2036) | 2036 (2035 to 2037) | 2036 (2035 to 2037) | 2035 (2034 to 2036) | 2036 (2034 to 2037) |
| PAHO | 2039 (2035 to 2045) | 2036 (2033 to 2039) | 2030 (2029 to 2030) | 2039 (2035 to 2045) | 2039 (2035 to 2045) | 2031 (2030 to 2032) | 2034 (2033 to 2035) |
| SEARO | 2052 (2048 to 2059) | 2051 (2047 to 2056) | 2040 (2036 to 2043) | 2052 (2048 to 2059) | 2052 (2048 to 2059) | 2040 (2036 to 2043) | 2043 (2041 to 2044) |
| WPRO | 2041 (2039 to 2043) | 2041 (2039 to 2043) | 2034 (2033 to 2036) | 2041 (2039 to 2043) | 2041 (2039 to 2043) | 2035 (2034 to 2036) | 2038 (2037 to 2039) |
| Global | after 2100 (2091 to after 2100) | 2082 (2076 to 2089) | 2047 (2045 to 2049) | after 2100 (2091 to after 2100) | after 2100 (2091 to after 2100) | 2047 (2045 to 2049) | 2047 (2046 to 2050) |
| Treatment level such that 40% of treatment-eligible individuals are in treatment in 2030 | | | | | | | |
| AFRO | after 2100 (after 2100 to after 2100) | 2098 (2093 to after 2100) | 2056 (2055 to 2058) | after 2100 (after 2100 to after 2100) | after 2100 (after 2100 to after 2100) | 2057 (2056 to 2059) | 2061 (2059 to 2063) |
| EMRO | after 2100 (2087 to after 2100) | 2086 (2072 to 2099) | 2042 (2037 to 2047) | after 2100 (2087 to after 2100) | after 2100 (2087 to after 2100) | 2042 (2037 to 2048) | 2045 (2044 to 2049) |
| EURO | 2036 (2034 to 2037) | 2036 (2034 to 2037) | 2035 (2033 to 2036) | 2036 (2034 to 2037) | 2036 (2034 to 2037) | 2035 (2034 to 2036) | 2035 (2034 to 2036) |
| PAHO | 2039 (2036 to 2041) | 2035 (2033 to 2037) | 2030 (2029 to 2030) | 2039 (2036 to 2041) | 2039 (2036 to 2041) | 2031 (2031 to 2032) | 2034 (2033 to 2035) |
| SEARO | 2050 (2047 to 2056) | 2049 (2046 to 2054) | 2039 (2035 to 2042) | 2050 (2047 to 2056) | 2050 (2047 to 2056) | 2039 (2035 to 2042) | 2042 (2041 to 2044) |
| WPRO | 2043 (2041 to 2044) | 2043 (2041 to 2044) | 2035 (2034 to 2036) | 2043 (2041 to 2044) | 2043 (2041 to 2044) | 2035 (2034 to 2036) | 2039 (2038 to 2040) |

| **WHO region** | **Status quo HepB3 & HepB-BD** | **HepB-BD scale-up (≥25%)** | **HepB-BD scale-up (≥90%)** | **HepB-BD disruptions (5%)** | **HepB-BD disruptions (20%)** | **delayed HepB-BD scale-up (2023 to 2030)** | **delayed HepB-BD scale-up (2025 to 2040)** |
|---|---|---|---|---|---|---|---|
| Global | after 2100 (2100 to after 2100) | 2086 (2082 to 2092) | 2049 (2047 to 2050) | after 2100 (2100 to after 2100) | after 2100 (2100 to after 2100) | 2049 (2048 to 2051) | 2050 (2049 to 2052) |
| Treatment level such that 80% of treatment-eligible individuals are in treatment in 2030 | | | | | | | |
| AFRO | 2084 (2080 to 2091) | 2074 (2071 to 2080) | 2049 (2048 to 2051) | 2084 (2080 to 2091) | 2084 (2080 to 2091) | 2050 (2048 to 2052) | 2050 (2049 to 2053) |
| EMRO | 2063 (2055 to 2075) | 2056 (2050 to 2063) | 2035 (2034 to 2035) | 2063 (2055 to 2075) | 2063 (2055 to 2075) | 2035 (2034 to 2035) | 2042 (2041 to 2043) |
| EURO | 2033 (2032 to 2034) | 2033 (2032 to 2034) | 2032 (2031 to 2034) | 2033 (2032 to 2034) | 2033 (2032 to 2034) | 2032 (2031 to 2034) | 2033 (2032 to 2034) |
| PAHO | 2032 (2029 to 2033) | 2030 (2029 to 2032) | 2028 (2027 to 2029) | 2032 (2029 to 2033) | 2032 (2029 to 2033) | 2029 (2029 to 2030) | 2031 (2029 to 2032) |
| SEARO | 2045 (2042 to 2048) | 2044 (2041 to 2047) | 2034 (2033 to 2035) | 2045 (2042 to 2048) | 2045 (2042 to 2048) | 2035 (2034 to 2035) | 2039 (2038 to 2041) |
| WPRO | 2038 (2036 to 2039) | 2038 (2036 to 2039) | 2033 (2032 to 2034) | 2038 (2036 to 2039) | 2038 (2036 to 2039) | 2033 (2032 to 2034) | 2036 (2034 to 2037) |
| Global | 2073 (2070 to 2079) | 2064 (2062 to 2068) | 2041 (2039 to 2042) | 2073 (2070 to 2079) | 2073 (2070 to 2079) | 2041 (2039 to 2042) | 2044 (2044 to 2045) |

**Supplementary Table 5** Mean number of HBV-related deaths averted in birth cohorts born between 2020 and 2030 in selected scenarios relative to the status quo HepB3 & HepB-BD scenario for each country modelled, with 95% credibility intervals in parentheses. Two target values are presented for the HepB-BD scale-up scenario i.e. timely HepB-BD expansion to ≥25% and ≥90% by 2030. The six WHO regions AFRO, EMRO, EURO, PAHO, SEARO and WPRO are shown in Supplementary Fig. 1. HBV: hepatitis B virus; HepB3: infant HBV vaccine series; timely HepB-BD: timely birth dose; ISO: International Organization for Standardization; WHO: World Health Organization.

| Country | ISO country code (WHO region) | HepB-BD scale-up (≥25%) | HepB-BD scale-up (≥90%) | HepB-BD disruptions (5%) | HepB-BD disruptions (20%) | delayed HepB-BD scale-up (2023 to 2030) | delayed HepB-BD scale-up (2025 to 2040) |
|---|---|---|---|---|---|---|---|
| Default settings | | | | | | | |
| Afghanistan | AFG (EMRO) | 0 (0 to 0) | 3,485 (1,835 to 5,865) | -23 (-36 to -13) | -91 (-146 to -52) | 2,525 (1,301 to 4,273) | 664 (336 to 1,131) |
| Angola | AGO (AFRO) | 6,418 (3,185 to 11,284) | 23,133 (11,501 to 40,618) | 0 (0 to 0) | 0 (0 to 0) | 17,122 (8,324 to 30,307) | 4,626 (2,206 to 8,236) |
| Albania | ALB (EURO) | 0 (0 to 0) | 0 (0 to 0) | 0 (0 to 0) | -1 (-2 to -1) | 0 (0 to 0) | 0 (0 to 0) |
| Armenia | ARM (EURO) | 0 (0 to 0) | 0 (0 to 0) | -3 (-6 to -2) | -13 (-24 to -6) | 0 (0 to 0) | 0 (0 to 0) |
| Azerbaijan | AZE (EURO) | 0 (0 to 0) | 0 (0 to 0) | -27 (-47 to -15) | -108 (-189 to -58) | 0 (0 to 0) | 0 (0 to 0) |
| Burundi | BDI (AFRO) | 503 (291 to 818) | 1,810 (1,047 to 2,944) | 0 (0 to 0) | 0 (0 to 0) | 1,329 (754 to 2,177) | 350 (195 to 575) |
| Benin | BEN (AFRO) | 2,546 (1,263 to 4,405) | 9,216 (4,568 to 15,961) | 0 (0 to 0) | 0 (0 to 0) | 6,476 (3,144 to 11,252) | 1,648 (788 to 2,865) |
| Burkina Faso | BFA (AFRO) | 1,613 (942 to 2,639) | 5,807 (3,390 to 9,498) | 0 (0 to 0) | 0 (0 to 0) | 4,108 (2,356 to 6,866) | 1,045 (591 to 1,774) |
| Bangladesh | BGD (SEARO) | 3,943 (2,163 to 6,947) | 14,190 (7,787 to 24,997) | 0 (0 to 0) | 0 (0 to 0) | 10,037 (5,403 to 17,899) | 2,564 (1,362 to 4,612) |
| Bosnia and Herzegovina | BIH (EURO) | 14 (7 to 23) | 51 (27 to 84) | 0 (0 to 0) | 0 (0 to 0) | 36 (19 to 60) | 9 (5 to 15) |
| Belarus | BLR (EURO) | 0 (0 to 0) | 0 (0 to 0) | -2 (-4 to -1) | -9 (-16 to -5) | 0 (0 to 0) | 0 (0 to 0) |
| Belize | BLZ (PAHO) | 0 (0 to 0) | 1 (1 to 2) | 0 (0 to 0) | 0 (0 to 0) | 1 (0 to 1) | 0 (0 to 0) |
| Bolivia | BOL (PAHO) | 123 (58 to 213) | 443 (207 to 767) | 0 (0 to 0) | 0 (0 to 0) | 314 (140 to 559) | 81 (35 to 148) |
| Bhutan | BTN (SEARO) | 0 (0 to 0) | 5 (2 to 8) | -1 (-2 to -1) | -5 (-8 to -3) | 3 (1 to 6) | 1 (0 to 1) |
| Central African Republic | CAF (AFRO) | 1,158 (542 to 2,114) | 4,201 (1,984 to 7,633) | 0 (0 to 0) | 0 (0 to 0) | 3,098 (1,411 to 5,693) | 829 (363 to 1,542) |
| China | CHN (WPRO) | 0 (0 to 0) | 0 (0 to 0) | -1,548 (-2,527 to -868) | -6,193 (-10,110 to -3,470) | 0 (0 to 0) | 0 (0 to 0) |
| Ivory Coast | CIV (AFRO) | 2,992 (1,226 to 5,639) | 15,149 (6,216 to 28,533) | -16 (-28 to -8) | -64 (-113 to -32) | 11,046 (4,340 to 21,095) | 2,904 (1,105 to 5,598) |
| Cameroon | CMR (AFRO) | 4,144 (2,225 to 7,177) | 14,918 (8,016 to 25,820) | 0 (0 to 0) | 0 (0 to 0) | 10,918 (5,743 to 19,111) | 2,910 (1,491 to 5,134) |
| Democratic Republic of the Congo | COD (AFRO) | 11,147 (4,978 to 22,199) | 40,138 (17,936 to 79,794) | 0 (0 to 0) | 0 (0 to 0) | 29,496 (12,773 to 59,789) | 7,932 (3,340 to 16,278) |

| Country | ISO country code (WHO region) | HepB-BD scale-up (≥25%) | HepB-BD scale-up (≥90%) | HepB-BD disruptions (5%) | HepB-BD disruptions (20%) | delayed HepB-BD scale-up (2023 to 2030) | delayed HepB-BD scale-up (2025 to 2040) |
|---|---|---|---|---|---|---|---|
| Congo | COG (AFRO) | 919 (409 to 1,645) | 3,312 (1,478 to 5,920) | 0 (0 to 0) | 0 (0 to 0) | 2,404 (1,038 to 4,362) | 630 (265 to 1,158) |
| Colombia | COL (PAHO) | 0 (0 to 0) | 27 (15 to 44) | -3 (-4 to -2) | -11 (-18 to -7) | 17 (10 to 29) | 4 (2 to 7) |
| Comoros | COM (AFRO) | 38 (16 to 75) | 136 (57 to 272) | 0 (0 to 0) | 0 (0 to 0) | 94 (39 to 189) | 24 (10 to 47) |
| Cape Verde | CPV (AFRO) | 0 (0 to 0) | 0 (0 to 0) | -3 (-6 to -2) | -13 (-23 to -7) | 0 (0 to 0) | 0 (0 to 0) |
| Cuba | CUB (PAHO) | 0 (0 to 0) | 0 (0 to 0) | -3 (-5 to -1) | -11 (-19 to -5) | 0 (0 to 0) | 0 (0 to 0) |
| Djibouti | DJI (EMRO) | 0 (0 to 0) | 0 (0 to 0) | -3 (-4 to -1) | -10 (-17 to -5) | 0 (0 to 0) | 0 (0 to 0) |
| Algeria | DZA (AFRO) | 0 (0 to 0) | 0 (0 to 0) | -48 (-80 to -27) | -192 (-320 to -108) | 0 (0 to 0) | 0 (0 to 0) |
| Ecuador | ECU (PAHO) | 0 (0 to 0) | 312 (161 to 532) | -13 (-21 to -8) | -53 (-85 to -31) | 221 (110 to 381) | 56 (27 to 99) |
| Egypt | EGY (EMRO) | 0 (0 to 0) | 0 (0 to 0) | -23 (-31 to -16) | -93 (-125 to -66) | 0 (0 to 0) | 0 (0 to 0) |
| Eritrea | ERI (AFRO) | 137 (66 to 245) | 494 (239 to 884) | 0 (0 to 0) | 0 (0 to 0) | 348 (160 to 635) | 89 (39 to 165) |
| Ethiopia | ETH (AFRO) | 7,478 (2,757 to 15,892) | 27,064 (9,979 to 57,499) | 0 (0 to 0) | 0 (0 to 0) | 18,973 (6,880 to 40,810) | 4,807 (1,720 to 10,442) |
| Fiji | FJI (WPRO) | 0 (0 to 0) | 0 (0 to 0) | -2 (-3 to -1) | -7 (-13 to -4) | 0 (0 to 0) | 0 (0 to 0) |
| Micronesia | FSM (WPRO) | 0 (0 to 0) | 6 (2 to 12) | 0 (0 to 0) | -1 (-2 to 0) | 4 (1 to 8) | 1 (0 to 2) |
| Georgia | GEO (EURO) | 0 (0 to 0) | 0 (0 to 0) | -6 (-10 to -3) | -22 (-41 to -11) | 0 (0 to 0) | 0 (0 to 0) |
| Ghana | GHA (AFRO) | 4,499 (2,556 to 7,228) | 16,185 (9,198 to 25,988) | 0 (0 to 0) | 0 (0 to 0) | 11,818 (6,583 to 19,161) | 3,090 (1,694 to 5,050) |
| Guinea | GIN (AFRO) | 7,118 (3,488 to 12,541) | 25,843 (12,748 to 45,476) | 0 (0 to 0) | 0 (0 to 0) | 18,894 (9,019 to 33,469) | 5,050 (2,334 to 9,051) |
| Gambia | GMB (AFRO) | 175 (70 to 347) | 629 (252 to 1,249) | 0 (0 to 0) | 0 (0 to 0) | 446 (168 to 910) | 115 (41 to 239) |
| Guinea-Bissau | GNB (AFRO) | 379 (193 to 676) | 1,362 (695 to 2,433) | 0 (0 to 0) | 0 (0 to 0) | 999 (499 to 1,799) | 264 (129 to 479) |
| Guatemala | GTM (PAHO) | 0 (0 to 0) | 114 (83 to 156) | -2 (-2 to -1) | -7 (-9 to -5) | 78 (56 to 107) | 19 (14 to 26) |
| Guyana | GUY (PAHO) | 14 (7 to 23) | 49 (25 to 82) | 0 (0 to 0) | 0 (0 to 0) | 34 (17 to 58) | 9 (4 to 15) |
| Honduras | HND (PAHO) | 0 (0 to 0) | 162 (81 to 292) | -10 (-17 to -6) | -41 (-70 to -23) | 111 (55 to 202) | 28 (13 to 51) |
| Haiti | HTI (PAHO) | 390 (198 to 742) | 1,404 (712 to 2,668) | 0 (0 to 0) | 0 (0 to 0) | 1,018 (511 to 1,945) | 271 (135 to 519) |
| Indonesia | IDN (SEARO) | 0 (0 to 0) | 2,402 (1,362 to 4,214) | -476 (-822 to -281) | -1,904 (-3,288 to -1,122) | 1,626 (918 to 2,848) | 406 (228 to 710) |
| India | IND (SEARO) | 0 (0 to 0) | 40,195 (21,501 to 73,926) | -673 (-1,189 to -373) | -2,691 (-4,758 to -1,491) | 28,770 (15,233 to 53,359) | 7,390 (3,879 to 13,791) |
| Iran | IRN (EMRO) | 0 (0 to 0) | 0 (0 to 0) | -48 (-86 to -18) | -193 (-345 to -70) | 0 (0 to 0) | 0 (0 to 0) |
| Iraq | IRQ (EMRO) | 0 (0 to 0) | 2,109 (1,189 to 3,745) | -21 (-37 to -12) | -86 (-147 to -50) | 1,468 (821 to 2,634) | 372 (207 to 673) |
| Jamaica | JAM (PAHO) | 9 (5 to 16) | 34 (18 to 58) | 0 (0 to 0) | 0 (0 to 0) | 23 (11 to 39) | 6 (3 to 10) |
| Jordan | JOR (EMRO) | 20 (12 to 31) | 73 (44 to 110) | 0 (0 to 0) | 0 (0 to 0) | 52 (30 to 79) | 13 (8 to 21) |
| Kenya | KEN (AFRO) | 210 (139 to 309) | 757 (500 to 1,111) | 0 (0 to 0) | 0 (0 to 0) | 499 (327 to 737) | 121 (79 to 179) |
| Kyrgyzstan | KGZ (EURO) | 0 (0 to 0) | 0 (0 to 0) | -16 (-23 to -11) | -63 (-90 to -43) | 0 (0 to 0) | 0 (0 to 0) |
| Cambodia | KHM (WPRO) | 0 (0 to 0) | 96 (54 to 156) | -49 (-76 to -29) | -197 (-306 to -118) | 66 (37 to 110) | 16 (9 to 28) |
| Kiribati | KIR (WPRO) | 0 (0 to 0) | 0 (0 to 0) | 0 (-1 to 0) | -2 (-3 to -1) | 0 (0 to 0) | 0 (0 to 0) |

| Country | ISO country code (WHO region) | HepB-BD scale-up (≥25%) | HepB-BD scale-up (≥90%) | HepB-BD disruptions (5%) | HepB-BD disruptions (20%) | delayed HepB-BD scale-up (2023 to 2030) | delayed HepB-BD scale-up (2025 to 2040) |
|---|---|---|---|---|---|---|---|
| Laos | LAO (WPRO) | 0 (0 to 0) | 584 (345 to 930) | -10 (-15 to -6) | -38 (-59 to -24) | 419 (241 to 669) | 110 (62 to 176) |
| Liberia | LBR (AFRO) | 2,144 (957 to 4,187) | 7,746 (3,465 to 15,055) | 0 (0 to 0) | 0 (0 to 0) | 5,616 (2,456 to 11,128) | 1,472 (632 to 2,971) |
| Sri Lanka | LKA (SEARO) | 231 (123 to 396) | 831 (441 to 1,425) | 0 (0 to 0) | 0 (0 to 0) | 570 (295 to 992) | 142 (72 to 249) |
| Lesotho | LSO (AFRO) | 81 (36 to 141) | 293 (128 to 508) | 0 (0 to 0) | 0 (0 to 0) | 206 (85 to 366) | 53 (21 to 95) |
| Morocco | MAR (EMRO) | 0 (0 to 0) | 114 (87 to 152) | -1 (-2 to -1) | -5 (-7 to -4) | 77 (59 to 104) | 19 (14 to 26) |
| Moldova | MDA (EURO) | 0 (0 to 0) | 0 (0 to 0) | -13 (-23 to -7) | -52 (-91 to -29) | 0 (0 to 0) | 0 (0 to 0) |
| Madagascar | MDG (AFRO) | 3,809 (2,346 to 6,179) | 13,709 (8,447 to 22,230) | 0 (0 to 0) | 0 (0 to 0) | 10,076 (6,172 to 16,407) | 2,658 (1,621 to 4,345) |
| Marshall Islands | MHL (WPRO) | 0 (0 to 0) | 0 (0 to 0) | 0 (-1 to 0) | -1 (-2 to -1) | 0 (0 to 0) | 0 (0 to 0) |
| North Macedonia | MKD (EURO) | 0 (0 to 0) | 0 (0 to 0) | -2 (-3 to -1) | -7 (-12 to -3) | 0 (0 to 0) | 0 (0 to 0) |
| Mali | MLI (AFRO) | 1,280 (711 to 2,130) | 4,613 (2,561 to 7,672) | 0 (0 to 0) | 0 (0 to 0) | 3,202 (1,743 to 5,423) | 810 (434 to 1,390) |
| Myanmar | MMR (SEARO) | 2,133 (1,146 to 3,785) | 19,476 (10,479 to 34,498) | -49 (-83 to -28) | -195 (-331 to -112) | 13,825 (7,279 to 24,785) | 3,544 (1,836 to 6,414) |
| Mongolia | MNG (WPRO) | 0 (0 to 0) | 0 (0 to 0) | -6 (-12 to -3) | -25 (-49 to -11) | 0 (0 to 0) | 0 (0 to 0) |
| Mozambique | MOZ (AFRO) | 4,802 (2,648 to 8,510) | 17,275 (9,529 to 30,598) | 0 (0 to 0) | 0 (0 to 0) | 12,726 (6,946 to 22,695) | 3,365 (1,820 to 6,028) |
| Mauritania | MRT (AFRO) | 747 (388 to 1,237) | 2,688 (1,398 to 4,451) | 0 (0 to 0) | 0 (0 to 0) | 1,976 (1,013 to 3,289) | 524 (266 to 879) |
| Malawi | MWI (AFRO) | 332 (194 to 557) | 1,195 (700 to 2,005) | 0 (0 to 0) | 0 (0 to 0) | 810 (468 to 1,385) | 202 (116 to 348) |
| Namibia | NAM (AFRO) | 0 (0 to 0) | 21 (12 to 37) | -2 (-3 to -1) | -8 (-13 to -4) | 15 (8 to 27) | 4 (2 to 7) |
| Niger | NER (AFRO) | 12,937 (6,679 to 22,967) | 46,600 (24,135 to 82,485) | 0 (0 to 0) | 0 (0 to 0) | 34,499 (17,436 to 61,804) | 9,171 (4,528 to 16,611) |
| Nigeria | NGA (AFRO) | 48,929 (22,321 to 89,657) | 178,909 (81,613 to 327,990) | 0 (0 to 0) | 0 (0 to 0) | 129,018 (58,281 to 236,990) | 33,404 (14,989 to 61,570) |
| Nicaragua | NIC (PAHO) | 10 (6 to 15) | 36 (21 to 55) | 0 (0 to 0) | 0 (0 to 0) | 24 (14 to 37) | 6 (3 to 9) |
| Nepal | NPL (SEARO) | 331 (185 to 565) | 1,193 (666 to 2,035) | 0 (0 to 0) | 0 (0 to 0) | 821 (442 to 1,447) | 205 (106 to 371) |
| Pakistan | PAK (EMRO) | 8,398 (4,531 to 13,569) | 30,228 (16,316 to 48,830) | 0 (0 to 0) | 0 (0 to 0) | 21,827 (11,352 to 35,704) | 5,730 (2,895 to 9,469) |
| Peru | PER (PAHO) | 0 (0 to 0) | 79 (49 to 115) | -10 (-14 to -7) | -42 (-57 to -28) | 54 (32 to 80) | 13 (8 to 20) |
| Philippines | PHL (WPRO) | 0 (0 to 0) | 4,903 (3,062 to 8,111) | -85 (-142 to -55) | -342 (-567 to -220) | 3,245 (2,028 to 5,360) | 795 (496 to 1,307) |
| Papua New Guinea | PNG (WPRO) | 0 (0 to 0) | 3,734 (1,781 to 6,916) | -14 (-25 to -8) | -57 (-98 to -31) | 2,671 (1,240 to 5,040) | 711 (325 to 1,358) |
| Korea, North | PRK (SEARO) | 0 (0 to 0) | 0 (0 to 0) | -139 (-239 to -67) | -554 (-959 to -266) | 0 (0 to 0) | 0 (0 to 0) |
| Paraguay | PRY (PAHO) | 107 (59 to 188) | 385 (211 to 678) | 0 (0 to 0) | 0 (0 to 0) | 272 (144 to 486) | 70 (36 to 126) |
| Rwanda | RWA (AFRO) | 326 (202 to 523) | 1,174 (729 to 1,884) | 0 (0 to 0) | 0 (0 to 0) | 850 (523 to 1,380) | 221 (134 to 362) |
| Sudan | SDN (EMRO) | 2,251 (1,202 to 4,097) | 8,102 (4,328 to 14,739) | 0 (0 to 0) | 0 (0 to 0) | 5,957 (3,152 to 10,936) | 1,567 (823 to 2,895) |

| Country | ISO country code (WHO region) | HepB-BD scale-up (≥25%) | HepB-BD scale-up (≥90%) | HepB-BD disruptions (5%) | HepB-BD disruptions (20%) | delayed HepB-BD scale-up (2023 to 2030) | delayed HepB-BD scale-up (2025 to 2040) |
|---|---|---|---|---|---|---|---|
| Senegal | SEN (AFRO) | 0 (0 to 0) | 830 (411 to 1,313) | -72 (-105 to -42) | -287 (-421 to -168) | 603 (288 to 969) | 157 (73 to 255) |
| Solomon Islands | SLB (WPRO) | 0 (0 to 0) | 20 (12 to 37) | -1 (-2 to -1) | -4 (-8 to -3) | 13 (8 to 24) | 3 (2 to 6) |
| Sierra Leone | SLE (AFRO) | 1,817 (957 to 3,207) | 6,560 (3,454 to 11,567) | 0 (0 to 0) | 0 (0 to 0) | 4,584 (2,374 to 8,170) | 1,153 (590 to 2,074) |
| El Salvador | SLV (PAHO) | 0 (0 to 0) | 24 (13 to 40) | -2 (-2 to -1) | -6 (-9 to -4) | 17 (9 to 28) | 4 (2 to 7) |
| Somalia | SOM (EMRO) | 3,091 (1,972 to 5,055) | 11,353 (7,242 to 18,625) | 0 (0 to 0) | 0 (0 to 0) | 8,367 (5,320 to 13,698) | 2,227 (1,408 to 3,640) |
| Serbia | SRB (EURO) | 0 (0 to 0) | 0 (0 to 0) | -5 (-9 to -2) | -19 (-35 to -9) | 0 (0 to 0) | 0 (0 to 0) |
| South Sudan | SSD (AFRO) | 1,430 (1,030 to 2,128) | 5,407 (3,881 to 8,040) | 0 (0 to 0) | 0 (0 to 0) | 3,915 (2,808 to 5,809) | 999 (720 to 1,477) |
| Sao Tome and Principe | STP (AFRO) | 0 (0 to 0) | 0 (0 to 0) | -1 (-1 to -1) | -4 (-6 to -2) | 0 (0 to 0) | 0 (0 to 0) |
| Eswatini | SWZ (AFRO) | 20 (8 to 36) | 72 (29 to 128) | 0 (0 to 0) | 0 (0 to 0) | 49 (19 to 89) | 12 (5 to 23) |
| Syria | SYR (EMRO) | 229 (135 to 391) | 827 (487 to 1,412) | 0 (0 to 0) | 0 (0 to 0) | 508 (298 to 871) | 120 (70 to 207) |
| Chad | TCD (AFRO) | 8,223 (3,867 to 15,825) | 29,779 (13,994 to 57,409) | 0 (0 to 0) | 0 (0 to 0) | 22,085 (10,311 to 42,697) | 5,976 (2,762 to 11,607) |
| Togo | TGO (AFRO) | 1,819 (936 to 3,298) | 6,547 (3,378 to 11,865) | 0 (0 to 0) | 0 (0 to 0) | 4,801 (2,406 to 8,733) | 1,266 (622 to 2,325) |
| Thailand | THA (SEARO) | 0 (0 to 0) | 0 (0 to 0) | -12 (-16 to -8) | -47 (-65 to -34) | 0 (0 to 0) | 0 (0 to 0) |
| Tajikistan | TJK (EURO) | 0 (0 to 0) | 0 (0 to 0) | -13 (-23 to -8) | -54 (-93 to -33) | 0 (0 to 0) | 0 (0 to 0) |
| Turkmenistan | TKM (EURO) | 0 (0 to 0) | 0 (0 to 0) | -10 (-19 to -6) | -42 (-76 to -25) | 0 (0 to 0) | 0 (0 to 0) |
| Timor-Leste | TLS (SEARO) | 0 (0 to 0) | 68 (36 to 115) | -2 (-4 to -1) | -9 (-14 to -5) | 49 (26 to 85) | 13 (7 to 22) |
| Tonga | TON (WPRO) | 0 (0 to 0) | 0 (0 to 0) | -1 (-1 to 0) | -3 (-5 to -2) | 0 (0 to 0) | 0 (0 to 0) |
| Tunisia | TUN (EMRO) | 0 (0 to 0) | 14 (10 to 21) | -1 (-2 to -1) | -5 (-8 to -4) | 8 (6 to 12) | 2 (1 to 3) |
| Tuvalu | TUV (WPRO) | 0 (0 to 0) | 0 (0 to 0) | 0 (0 to 0) | 0 (0 to 0) | 0 (0 to 0) | 0 (0 to 0) |
| Tanzania | TZA (AFRO) | 3,466 (1,663 to 6,570) | 12,473 (5,986 to 23,635) | 0 (0 to 0) | 0 (0 to 0) | 9,135 (4,307 to 17,565) | 2,409 (1,121 to 4,679) |
| Uganda | UGA (AFRO) | 4,866 (2,790 to 8,318) | 17,511 (10,042 to 29,923) | 0 (0 to 0) | 0 (0 to 0) | 12,771 (7,262 to 21,987) | 3,339 (1,887 to 5,776) |
| Ukraine | UKR (EURO) | 0 (0 to 0) | 483 (231 to 859) | -14 (-23 to -7) | -54 (-93 to -28) | 325 (152 to 583) | 80 (37 to 144) |
| Uzbekistan | UZB (EURO) | 0 (0 to 0) | 0 (0 to 0) | -89 (-211 to -45) | -355 (-844 to -179) | 0 (0 to 0) | 0 (0 to 0) |
| Venezuela | VEN (PAHO) | 0 (0 to 0) | 468 (214 to 790) | -8 (-13 to -4) | -33 (-54 to -18) | 319 (139 to 543) | 80 (33 to 137) |
| Viet Nam | VNM (WPRO) | 0 (0 to 0) | 2,629 (1,244 to 4,556) | -221 (-372 to -118) | -886 (-1,487 to -471) | 1,847 (854 to 3,233) | 468 (213 to 823) |
| Vanuatu | VUT (WPRO) | 0 (0 to 0) | 27 (10 to 52) | -3 (-5 to -1) | -11 (-20 to -5) | 19 (7 to 38) | 5 (2 to 10) |
| Samoa | WSM (WPRO) | 0 (0 to 0) | 13 (6 to 22) | 0 (-1 to 0) | -2 (-3 to -1) | 9 (4 to 15) | 2 (1 to 4) |
| Yemen | YEM (EMRO) | 1,297 (634 to 2,337) | 4,666 (2,284 to 8,407) | 0 (0 to 0) | 0 (0 to 0) | 3,379 (1,616 to 6,162) | 889 (419 to 1,635) |
| South Africa | ZAF (AFRO) | 2,601 (1,067 to 4,544) | 9,362 (3,843 to 16,351) | 0 (0 to 0) | 0 (0 to 0) | 6,705 (2,615 to 11,829) | 1,749 (663 to 3,106) |

| Country | ISO country code (WHO region) | HepB-BD scale-up (≥25%) | HepB-BD scale-up (≥90%) | HepB-BD disruptions (5%) | HepB-BD disruptions (20%) | delayed HepB-BD scale-up (2023 to 2030) | delayed HepB-BD scale-up (2025 to 2040) |
|---|---|---|---|---|---|---|---|
| Zambia | ZMB (AFRO) | 0 (0 to 0) | 0 (0 to 0) | -48 (-71 to -30) | -191 (-284 to -121) | 0 (0 to 0) | 0 (0 to 0) |
| Zimbabwe | ZWE (AFRO) | 389 (217 to 645) | 1,399 (781 to 2,324) | 0 (0 to 0) | 0 (0 to 0) | 948 (518 to 1,596) | 236 (127 to 402) |
| HepB3 coverage scaled up to 100% coverage in 2020 | | | | | | | |
| Afghanistan | AFG (EMRO) | 0 (0 to 0) | 2,889 (1,431 to 4,944) | -20 (-32 to -11) | -79 (-127 to -43) | 2,090 (1,008 to 3,627) | 540 (255 to 950) |
| Angola | AGO (AFRO) | 5,324 (2,322 to 9,771) | 19,153 (8,358 to 35,131) | 0 (0 to 0) | 0 (0 to 0) | 14,134 (6,015 to 26,119) | 3,717 (1,552 to 6,911) |
| Albania | ALB (EURO) | 0 (0 to 0) | 0 (0 to 0) | 0 (0 to 0) | -1 (-2 to -1) | 0 (0 to 0) | 0 (0 to 0) |
| Armenia | ARM (EURO) | 0 (0 to 0) | 0 (0 to 0) | -3 (-6 to -2) | -13 (-23 to -6) | 0 (0 to 0) | 0 (0 to 0) |
| Azerbaijan | AZE (EURO) | 0 (0 to 0) | 0 (0 to 0) | -26 (-47 to -14) | -105 (-186 to -56) | 0 (0 to 0) | 0 (0 to 0) |
| Burundi | BDI (AFRO) | 494 (282 to 805) | 1,778 (1,015 to 2,896) | 0 (0 to 0) | 0 (0 to 0) | 1,305 (729 to 2,140) | 342 (188 to 562) |
| Benin | BEN (AFRO) | 2,133 (1,039 to 3,683) | 7,679 (3,738 to 13,263) | 0 (0 to 0) | 0 (0 to 0) | 5,385 (2,574 to 9,328) | 1,361 (643 to 2,372) |
| Burkina Faso | BFA (AFRO) | 1,513 (882 to 2,486) | 5,444 (3,176 to 8,948) | 0 (0 to 0) | 0 (0 to 0) | 3,848 (2,205 to 6,463) | 975 (550 to 1,654) |
| Bangladesh | BGD (SEARO) | 3,924 (2,148 to 6,925) | 14,121 (7,733 to 24,918) | 0 (0 to 0) | 0 (0 to 0) | 9,987 (5,364 to 17,840) | 2,549 (1,351 to 4,592) |
| Bosnia and Herzegovina | BIH (EURO) | 13 (7 to 22) | 47 (24 to 79) | 0 (0 to 0) | 0 (0 to 0) | 33 (16 to 57) | 8 (4 to 15) |
| Belarus | BLR (EURO) | 0 (0 to 0) | 0 (0 to 0) | -2 (-4 to -1) | -9 (-16 to -5) | 0 (0 to 0) | 0 (0 to 0) |
| Belize | BLZ (PAHO) | 0 (0 to 0) | 1 (1 to 2) | 0 (0 to 0) | 0 (0 to 0) | 1 (0 to 1) | 0 (0 to 0) |
| Bolivia | BOL (PAHO) | 108 (48 to 198) | 389 (173 to 714) | 0 (0 to 0) | 0 (0 to 0) | 275 (116 to 519) | 70 (29 to 135) |
| Bhutan | BTN (SEARO) | 0 (0 to 0) | 5 (2 to 8) | -1 (-2 to -1) | -5 (-8 to -3) | 3 (1 to 6) | 1 (0 to 1) |
| Central African Republic | CAF (AFRO) | 909 (376 to 1,773) | 3,268 (1,353 to 6,365) | 0 (0 to 0) | 0 (0 to 0) | 2,406 (976 to 4,728) | 631 (251 to 1,249) |
| China | CHN (WPRO) | 0 (0 to 0) | 0 (0 to 0) | -1,543 (-2,519 to -864) | -6,173 (-10,080 to -3,457) | 0 (0 to 0) | 0 (0 to 0) |
| Ivory Coast | CIV (AFRO) | 2,784 (1,089 to 5,308) | 14,084 (5,514 to 26,838) | -15 (-27 to -8) | -61 (-108 to -30) | 10,254 (3,843 to 19,809) | 2,673 (971 to 5,211) |
| Cameroon | CMR (AFRO) | 3,619 (1,808 to 6,470) | 13,021 (6,509 to 23,265) | 0 (0 to 0) | 0 (0 to 0) | 9,510 (4,653 to 17,172) | 2,484 (1,191 to 4,515) |
| Democratic Republic of the Congo | COD (AFRO) | 9,186 (3,608 to 19,781) | 33,055 (12,987 to 71,144) | 0 (0 to 0) | 0 (0 to 0) | 24,243 (9,292 to 53,019) | 6,346 (2,385 to 14,079) |
| Congo | COG (AFRO) | 829 (353 to 1,526) | 2,984 (1,269 to 5,484) | 0 (0 to 0) | 0 (0 to 0) | 2,162 (895 to 4,024) | 561 (227 to 1,054) |
| Colombia | COL (PAHO) | 0 (0 to 0) | 25 (14 to 42) | -3 (-4 to -2) | -11 (-17 to -7) | 16 (9 to 27) | 4 (2 to 7) |
| Comoros | COM (AFRO) | 35 (15 to 71) | 128 (54 to 254) | 0 (0 to 0) | 0 (0 to 0) | 88 (37 to 176) | 22 (9 to 44) |
| Cape Verde | CPV (AFRO) | 0 (0 to 0) | 0 (0 to 0) | -3 (-6 to -2) | -13 (-23 to -7) | 0 (0 to 0) | 0 (0 to 0) |
| Cuba | CUB (PAHO) | 0 (0 to 0) | 0 (0 to 0) | -3 (-5 to -1) | -11 (-19 to -5) | 0 (0 to 0) | 0 (0 to 0) |

| Country | ISO country code (WHO region) | HepB-BD scale-up (≥25%) | HepB-BD scale-up (≥90%) | HepB-BD disruptions (5%) | HepB-BD disruptions (20%) | delayed HepB-BD scale-up (2023 to 2030) | delayed HepB-BD scale-up (2025 to 2040) |
|---|---|---|---|---|---|---|---|
| Djibouti | DJI (EMRO) | 0 (0 to 0) | 0 (0 to 0) | -2 (-4 to -1) | -10 (-17 to -5) | 0 (0 to 0) | 0 (0 to 0) |
| Algeria | DZA (AFRO) | 0 (0 to 0) | 0 (0 to 0) | -47 (-79 to -26) | -188 (-315 to -104) | 0 (0 to 0) | 0 (0 to 0) |
| Ecuador | ECU (PAHO) | 0 (0 to 0) | 297 (147 to 518) | -13 (-21 to -7) | -51 (-82 to -30) | 210 (100 to 372) | 54 (25 to 96) |
| Egypt | EGY (EMRO) | 0 (0 to 0) | 0 (0 to 0) | -23 (-31 to -16) | -90 (-122 to -64) | 0 (0 to 0) | 0 (0 to 0) |
| Eritrea | ERI (AFRO) | 133 (64 to 238) | 480 (230 to 858) | 0 (0 to 0) | 0 (0 to 0) | 338 (154 to 616) | 86 (38 to 160) |
| Ethiopia | ETH (AFRO) | 5,474 (1,918 to 11,974) | 19,710 (6,903 to 43,110) | 0 (0 to 0) | 0 (0 to 0) | 13,863 (4,768 to 30,677) | 3,498 (1,186 to 7,813) |
| Fiji | FJI (WPRO) | 0 (0 to 0) | 0 (0 to 0) | -2 (-3 to -1) | -7 (-13 to -4) | 0 (0 to 0) | 0 (0 to 0) |
| Micronesia | FSM (WPRO) | 0 (0 to 0) | 6 (2 to 11) | 0 (0 to 0) | -1 (-2 to 0) | 4 (1 to 8) | 1 (0 to 2) |
| Georgia | GEO (EURO) | 0 (0 to 0) | 0 (0 to 0) | -5 (-10 to -3) | -22 (-40 to -11) | 0 (0 to 0) | 0 (0 to 0) |
| Ghana | GHA (AFRO) | 4,466 (2,525 to 7,189) | 16,064 (9,086 to 25,846) | 0 (0 to 0) | 0 (0 to 0) | 11,726 (6,500 to 19,051) | 3,061 (1,671 to 5,015) |
| Guinea | GIN (AFRO) | 5,574 (2,361 to 10,534) | 20,031 (8,497 to 37,802) | 0 (0 to 0) | 0 (0 to 0) | 14,618 (5,972 to 27,922) | 3,819 (1,516 to 7,374) |
| Gambia | GMB (AFRO) | 163 (64 to 327) | 585 (229 to 1,176) | 0 (0 to 0) | 0 (0 to 0) | 414 (152 to 856) | 106 (37 to 224) |
| Guinea-Bissau | GNB (AFRO) | 353 (173 to 637) | 1,269 (622 to 2,289) | 0 (0 to 0) | 0 (0 to 0) | 929 (443 to 1,697) | 243 (113 to 448) |
| Guatemala | GTM (PAHO) | 0 (0 to 0) | 100 (73 to 137) | -1 (-2 to -1) | -6 (-8 to -5) | 68 (49 to 94) | 17 (12 to 23) |
| Guyana | GUY (PAHO) | 14 (7 to 23) | 49 (24 to 81) | 0 (0 to 0) | 0 (0 to 0) | 34 (17 to 58) | 9 (4 to 15) |
| Honduras | HND (PAHO) | 0 (0 to 0) | 153 (75 to 277) | -10 (-17 to -5) | -39 (-68 to -22) | 105 (50 to 192) | 26 (12 to 48) |
| Haiti | HTI (PAHO) | 335 (154 to 666) | 1,207 (554 to 2,395) | 0 (0 to 0) | 0 (0 to 0) | 872 (397 to 1,739) | 226 (102 to 453) |
| Indonesia | IDN (SEARO) | 0 (0 to 0) | 2,161 (1,221 to 3,786) | -445 (-766 to -262) | -1,780 (-3,064 to -1,049) | 1,459 (821 to 2,555) | 361 (203 to 633) |
| India | IND (SEARO) | 0 (0 to 0) | 37,867 (20,196 to 69,952) | -648 (-1,152 to -358) | -2,594 (-4,608 to -1,432) | 27,075 (14,280 to 50,431) | 6,926 (3,622 to 12,976) |
| Iran | IRN (EMRO) | 0 (0 to 0) | 0 (0 to 0) | -48 (-86 to -18) | -192 (-344 to -70) | 0 (0 to 0) | 0 (0 to 0) |
| Iraq | IRQ (EMRO) | 0 (0 to 0) | 2,014 (1,109 to 3,652) | -21 (-36 to -12) | -83 (-143 to -48) | 1,399 (764 to 2,565) | 352 (191 to 651) |
| Jamaica | JAM (PAHO) | 9 (5 to 16) | 33 (17 to 57) | 0 (0 to 0) | 0 (0 to 0) | 22 (11 to 39) | 6 (3 to 10) |
| Jordan | JOR (EMRO) | 20 (12 to 30) | 72 (42 to 109) | 0 (0 to 0) | 0 (0 to 0) | 51 (29 to 78) | 13 (7 to 20) |
| Kenya | KEN (AFRO) | 198 (131 to 290) | 713 (471 to 1,045) | 0 (0 to 0) | 0 (0 to 0) | 470 (308 to 693) | 113 (74 to 168) |
| Kyrgyzstan | KGZ (EURO) | 0 (0 to 0) | 0 (0 to 0) | -16 (-22 to -11) | -62 (-90 to -42) | 0 (0 to 0) | 0 (0 to 0) |
| Cambodia | KHM (WPRO) | 0 (0 to 0) | 91 (51 to 150) | -48 (-74 to -29) | -191 (-296 to -114) | 63 (35 to 105) | 16 (8 to 27) |
| Kiribati | KIR (WPRO) | 0 (0 to 0) | 0 (0 to 0) | 0 (-1 to 0) | -2 (-3 to -1) | 0 (0 to 0) | 0 (0 to 0) |
| Laos | LAO (WPRO) | 0 (0 to 0) | 527 (290 to 853) | -9 (-14 to -5) | -36 (-55 to -22) | 378 (203 to 617) | 97 (52 to 160) |
| Liberia | LBR (AFRO) | 1,908 (809 to 3,862) | 6,859 (2,911 to 13,865) | 0 (0 to 0) | 0 (0 to 0) | 4,963 (2,056 to 10,105) | 1,288 (522 to 2,636) |
| Sri Lanka | LKA (SEARO) | 229 (122 to 394) | 826 (438 to 1,417) | 0 (0 to 0) | 0 (0 to 0) | 566 (293 to 986) | 141 (71 to 247) |
| Lesotho | LSO (AFRO) | 77 (32 to 136) | 276 (117 to 488) | 0 (0 to 0) | 0 (0 to 0) | 194 (78 to 351) | 49 (19 to 91) |

| Country | ISO country code (WHO region) | HepB-BD scale-up (≥25%) | HepB-BD scale-up (≥90%) | HepB-BD disruptions (5%) | HepB-BD disruptions (20%) | delayed HepB-BD scale-up (2023 to 2030) | delayed HepB-BD scale-up (2025 to 2040) |
|---|---|---|---|---|---|---|---|
| Morocco | MAR (EMRO) | 0 (0 to 0) | 113 (87 to 151) | -1 (-2 to -1) | -5 (-7 to -4) | 77 (58 to 104) | 19 (14 to 26) |
| Moldova | MDA (EURO) | 0 (0 to 0) | 0 (0 to 0) | -13 (-22 to -7) | -52 (-90 to -29) | 0 (0 to 0) | 0 (0 to 0) |
| Madagascar | MDG (AFRO) | 3,613 (2,225 to 5,863) | 12,999 (8,008 to 21,088) | 0 (0 to 0) | 0 (0 to 0) | 9,538 (5,847 to 15,539) | 2,496 (1,524 to 4,083) |
| Marshall Islands | MHL (WPRO) | 0 (0 to 0) | 0 (0 to 0) | 0 (-1 to 0) | -1 (-2 to -1) | 0 (0 to 0) | 0 (0 to 0) |
| North Macedonia | MKD (EURO) | 0 (0 to 0) | 0 (0 to 0) | -2 (-3 to -1) | -6 (-12 to -3) | 0 (0 to 0) | 0 (0 to 0) |
| Mali | MLI (AFRO) | 1,018 (544 to 1,744) | 3,663 (1,959 to 6,278) | 0 (0 to 0) | 0 (0 to 0) | 2,548 (1,334 to 4,410) | 641 (330 to 1,117) |
| Myanmar | MMR (SEARO) | 2,010 (1,065 to 3,602) | 18,324 (9,723 to 32,795) | -47 (-80 to -27) | -187 (-319 to -107) | 12,995 (6,746 to 23,557) | 3,316 (1,706 to 6,066) |
| Mongolia | MNG (WPRO) | 0 (0 to 0) | 0 (0 to 0) | -6 (-12 to -3) | -24 (-49 to -10) | 0 (0 to 0) | 0 (0 to 0) |
| Mozambique | MOZ (AFRO) | 4,676 (2,552 to 8,326) | 16,822 (9,186 to 29,939) | 0 (0 to 0) | 0 (0 to 0) | 12,380 (6,680 to 22,183) | 3,251 (1,740 to 5,851) |
| Mauritania | MRT (AFRO) | 705 (359 to 1,189) | 2,534 (1,291 to 4,275) | 0 (0 to 0) | 0 (0 to 0) | 1,860 (934 to 3,173) | 488 (242 to 838) |
| Malawi | MWI (AFRO) | 320 (188 to 538) | 1,153 (675 to 1,935) | 0 (0 to 0) | 0 (0 to 0) | 781 (452 to 1,335) | 194 (111 to 334) |
| Namibia | NAM (AFRO) | 0 (0 to 0) | 19 (11 to 34) | -2 (-3 to -1) | -7 (-12 to -4) | 14 (8 to 25) | 4 (2 to 6) |
| Niger | NER (AFRO) | 11,965 (5,915 to 21,627) | 43,005 (21,296 to 77,581) | 0 (0 to 0) | 0 (0 to 0) | 31,791 (15,443 to 57,771) | 8,367 (3,999 to 15,366) |
| Nigeria | NGA (AFRO) | 35,366 (15,629 to 65,481) | 127,333 (56,308 to 235,667) | 0 (0 to 0) | 0 (0 to 0) | 91,985 (40,172 to 170,621) | 23,725 (10,301 to 44,343) |
| Nicaragua | NIC (PAHO) | 10 (6 to 15) | 35 (21 to 54) | 0 (0 to 0) | 0 (0 to 0) | 24 (13 to 37) | 6 (3 to 9) |
| Nepal | NPL (SEARO) | 321 (177 to 556) | 1,155 (638 to 2,001) | 0 (0 to 0) | 0 (0 to 0) | 794 (421 to 1,426) | 198 (101 to 365) |
| Pakistan | PAK (EMRO) | 7,537 (3,771 to 12,547) | 27,127 (13,576 to 45,151) | 0 (0 to 0) | 0 (0 to 0) | 19,543 (9,420 to 32,971) | 5,056 (2,369 to 8,621) |
| Peru | PER (PAHO) | 0 (0 to 0) | 73 (44 to 107) | -10 (-14 to -7) | -39 (-55 to -26) | 50 (30 to 74) | 12 (7 to 19) |
| Philippines | PHL (WPRO) | 0 (0 to 0) | 3,542 (2,188 to 5,842) | -68 (-111 to -43) | -270 (-446 to -174) | 2,344 (1,445 to 3,870) | 570 (350 to 938) |
| Papua New Guinea | PNG (WPRO) | 0 (0 to 0) | 2,531 (1,029 to 5,280) | -10 (-20 to -5) | -42 (-81 to -19) | 1,813 (721 to 3,825) | 467 (183 to 993) |
| Korea, North | PRK (SEARO) | 0 (0 to 0) | 0 (0 to 0) | -137 (-237 to -66) | -549 (-951 to -263) | 0 (0 to 0) | 0 (0 to 0) |
| Paraguay | PRY (PAHO) | 102 (54 to 184) | 367 (194 to 661) | 0 (0 to 0) | 0 (0 to 0) | 258 (132 to 469) | 66 (32 to 121) |
| Rwanda | RWA (AFRO) | 325 (202 to 522) | 1,169 (726 to 1,879) | 0 (0 to 0) | 0 (0 to 0) | 846 (520 to 1,376) | 220 (133 to 361) |
| Sudan | SDN (EMRO) | 2,216 (1,178 to 4,075) | 7,976 (4,242 to 14,662) | 0 (0 to 0) | 0 (0 to 0) | 5,861 (3,079 to 10,873) | 1,536 (801 to 2,866) |
| Senegal | SEN (AFRO) | 0 (0 to 0) | 807 (391 to 1,289) | -70 (-104 to -41) | -282 (-415 to -163) | 587 (274 to 950) | 152 (69 to 249) |
| Solomon Islands | SLB (WPRO) | 0 (0 to 0) | 19 (12 to 35) | -1 (-2 to -1) | -4 (-7 to -3) | 12 (7 to 22) | 3 (2 to 5) |
| Sierra Leone | SLE (AFRO) | 1,746 (917 to 3,091) | 6,287 (3,304 to 11,118) | 0 (0 to 0) | 0 (0 to 0) | 4,390 (2,269 to 7,855) | 1,104 (564 to 1,988) |
| El Salvador | SLV (PAHO) | 0 (0 to 0) | 23 (12 to 38) | -1 (-2 to -1) | -6 (-9 to -4) | 16 (8 to 27) | 4 (2 to 7) |

| Country | ISO country code (WHO region) | HepB-BD scale-up (≥25%) | HepB-BD scale-up (≥90%) | HepB-BD disruptions (5%) | HepB-BD disruptions (20%) | delayed HepB-BD scale-up (2023 to 2030) | delayed HepB-BD scale-up (2025 to 2040) |
|---|---|---|---|---|---|---|---|
| Somalia | SOM (EMRO) | 2,311 (1,377 to 3,899) | 8,325 (4,962 to 14,024) | 0 (0 to 0) | 0 (0 to 0) | 6,138 (3,660 to 10,313) | 1,608 (960 to 2,693) |
| Serbia | SRB (EURO) | 0 (0 to 0) | 0 (0 to 0) | -5 (-9 to -2) | -18 (-34 to -9) | 0 (0 to 0) | 0 (0 to 0) |
| South Sudan | SSD (AFRO) | 1,000 (675 to 1,566) | 3,612 (2,433 to 5,641) | 0 (0 to 0) | 0 (0 to 0) | 2,629 (1,768 to 4,081) | 683 (459 to 1,054) |
| Sao Tome and Principe | STP (AFRO) | 0 (0 to 0) | 0 (0 to 0) | -1 (-1 to -1) | -4 (-6 to -2) | 0 (0 to 0) | 0 (0 to 0) |
| Eswatini | SWZ (AFRO) | 19 (8 to 33) | 68 (27 to 120) | 0 (0 to 0) | 0 (0 to 0) | 46 (18 to 83) | 11 (4 to 21) |
| Syria | SYR (EMRO) | 153 (88 to 267) | 552 (317 to 961) | 0 (0 to 0) | 0 (0 to 0) | 339 (194 to 593) | 79 (45 to 139) |
| Chad | TCD (AFRO) | 6,619 (2,940 to 13,265) | 23,791 (10,577 to 47,598) | 0 (0 to 0) | 0 (0 to 0) | 17,600 (7,716 to 35,462) | 4,639 (2,008 to 9,406) |
| Togo | TGO (AFRO) | 1,705 (842 to 3,135) | 6,133 (3,031 to 11,265) | 0 (0 to 0) | 0 (0 to 0) | 4,492 (2,188 to 8,350) | 1,174 (565 to 2,206) |
| Thailand | THA (SEARO) | 0 (0 to 0) | 0 (0 to 0) | -12 (-16 to -8) | -47 (-64 to -33) | 0 (0 to 0) | 0 (0 to 0) |
| Tajikistan | TJK (EURO) | 0 (0 to 0) | 0 (0 to 0) | -13 (-23 to -8) | -53 (-92 to -32) | 0 (0 to 0) | 0 (0 to 0) |
| Turkmenistan | TKM (EURO) | 0 (0 to 0) | 0 (0 to 0) | -10 (-19 to -6) | -42 (-76 to -25) | 0 (0 to 0) | 0 (0 to 0) |
| Timor-Leste | TLS (SEARO) | 0 (0 to 0) | 63 (33 to 111) | -2 (-4 to -1) | -8 (-14 to -5) | 46 (24 to 82) | 12 (6 to 21) |
| Tonga | TON (WPRO) | 0 (0 to 0) | 0 (0 to 0) | -1 (-1 to 0) | -3 (-5 to -2) | 0 (0 to 0) | 0 (0 to 0) |
| Tunisia | TUN (EMRO) | 0 (0 to 0) | 13 (9 to 19) | -1 (-2 to -1) | -5 (-8 to -4) | 8 (6 to 12) | 2 (1 to 3) |
| Tuvalu | TUV (WPRO) | 0 (0 to 0) | 0 (0 to 0) | 0 (0 to 0) | 0 (0 to 0) | 0 (0 to 0) | 0 (0 to 0) |
| Tanzania | TZA (AFRO) | 3,345 (1,586 to 6,408) | 12,037 (5,709 to 23,057) | 0 (0 to 0) | 0 (0 to 0) | 8,808 (4,101 to 17,120) | 2,306 (1,056 to 4,527) |
| Uganda | UGA (AFRO) | 4,764 (2,726 to 8,161) | 17,142 (9,812 to 29,357) | 0 (0 to 0) | 0 (0 to 0) | 12,493 (7,090 to 21,557) | 3,255 (1,836 to 5,644) |
| Ukraine | UKR (EURO) | 0 (0 to 0) | 424 (195 to 760) | -12 (-22 to -6) | -50 (-86 to -25) | 284 (127 to 512) | 70 (31 to 126) |
| Uzbekistan | UZB (EURO) | 0 (0 to 0) | 0 (0 to 0) | -87 (-208 to -44) | -349 (-832 to -175) | 0 (0 to 0) | 0 (0 to 0) |
| Venezuela | VEN (PAHO) | 0 (0 to 0) | 400 (163 to 699) | -7 (-12 to -4) | -30 (-49 to -15) | 272 (106 to 482) | 67 (25 to 120) |
| Viet Nam | VNM (WPRO) | 0 (0 to 0) | 2,507 (1,151 to 4,399) | -215 (-362 to -112) | -859 (-1,447 to -447) | 1,759 (789 to 3,104) | 444 (196 to 786) |
| Vanuatu | VUT (WPRO) | 0 (0 to 0) | 25 (9 to 50) | -3 (-5 to -1) | -11 (-19 to -4) | 18 (7 to 37) | 5 (2 to 10) |
| Samoa | WSM (WPRO) | 0 (0 to 0) | 10 (5 to 19) | 0 (-1 to 0) | -2 (-3 to -1) | 7 (4 to 13) | 2 (1 to 3) |
| Yemen | YEM (EMRO) | 1,198 (552 to 2,232) | 4,310 (1,988 to 8,033) | 0 (0 to 0) | 0 (0 to 0) | 3,115 (1,403 to 5,876) | 808 (358 to 1,535) |
| South Africa | ZAF (AFRO) | 2,399 (902 to 4,302) | 8,632 (3,248 to 15,474) | 0 (0 to 0) | 0 (0 to 0) | 6,171 (2,226 to 11,182) | 1,591 (561 to 2,899) |
| Zambia | ZMB (AFRO) | 0 (0 to 0) | 0 (0 to 0) | -46 (-68 to -29) | -183 (-274 to -116) | 0 (0 to 0) | 0 (0 to 0) |
| Zimbabwe | ZWE (AFRO) | 362 (202 to 602) | 1,305 (727 to 2,169) | 0 (0 to 0) | 0 (0 to 0) | 883 (482 to 1,489) | 219 (118 to 373) |
| Treatment level such that 40% of treatment-eligible individuals are in treatment in 2030 | | | | | | | |
| Afghanistan | AFG (EMRO) | 0 (0 to 0) | 2,040 (1,536 to 2,456) | -14 (-17 to -11) | -57 (-68 to -45) | 1,463 (1,089 to 1,777) | 382 (280 to 467) |

| Country | ISO country code (WHO region) | HepB-BD scale-up (≥25%) | HepB-BD scale-up (≥90%) | HepB-BD disruptions (5%) | HepB-BD disruptions (20%) | delayed HepB-BD scale-up (2023 to 2030) | delayed HepB-BD scale-up (2025 to 2040) |
|---|---|---|---|---|---|---|---|
| Angola | AGO (AFRO) | 3,635 (2,449 to 4,219) | 13,101 (8,847 to 15,186) | 0 (0 to 0) | 0 (0 to 0) | 9,632 (6,337 to 11,268) | 2,587 (1,661 to 3,048) |
| Albania | ALB (EURO) | 0 (0 to 0) | 0 (0 to 0) | 0 (0 to 0) | -1 (-1 to -1) | 0 (0 to 0) | 0 (0 to 0) |
| Armenia | ARM (EURO) | 0 (0 to 0) | 0 (0 to 0) | -2 (-3 to -1) | -8 (-11 to -5) | 0 (0 to 0) | 0 (0 to 0) |
| Azerbaijan | AZE (EURO) | 0 (0 to 0) | 0 (0 to 0) | -17 (-23 to -11) | -67 (-92 to -46) | 0 (0 to 0) | 0 (0 to 0) |
| Burundi | BDI (AFRO) | 312 (259 to 350) | 1,123 (933 to 1,260) | 0 (0 to 0) | 0 (0 to 0) | 818 (670 to 923) | 214 (174 to 243) |
| Benin | BEN (AFRO) | 1,533 (1,072 to 2,019) | 5,547 (3,879 to 7,300) | 0 (0 to 0) | 0 (0 to 0) | 3,881 (2,636 to 5,205) | 985 (655 to 1,334) |
| Burkina Faso | BFA (AFRO) | 1,028 (785 to 1,296) | 3,703 (2,828 to 4,666) | 0 (0 to 0) | 0 (0 to 0) | 2,593 (1,957 to 3,331) | 655 (490 to 858) |
| Bangladesh | BGD (SEARO) | 2,474 (1,910 to 3,054) | 8,905 (6,877 to 10,993) | 0 (0 to 0) | 0 (0 to 0) | 6,249 (4,719 to 7,789) | 1,587 (1,187 to 1,989) |
| Bosnia and Herzegovina | BIH (EURO) | 8 (6 to 10) | 30 (22 to 36) | 0 (0 to 0) | 0 (0 to 0) | 21 (15 to 26) | 5 (4 to 7) |
| Belarus | BLR (EURO) | 0 (0 to 0) | 0 (0 to 0) | -1 (-2 to -1) | -6 (-7 to -4) | 0 (0 to 0) | 0 (0 to 0) |
| Belize | BLZ (PAHO) | 0 (0 to 0) | 1 (1 to 1) | 0 (0 to 0) | 0 (0 to 0) | 1 (0 to 1) | 0 (0 to 0) |
| Bolivia | BOL (PAHO) | 79 (46 to 109) | 285 (166 to 391) | 0 (0 to 0) | 0 (0 to 0) | 200 (110 to 281) | 51 (27 to 74) |
| Bhutan | BTN (SEARO) | 0 (0 to 0) | 3 (1 to 4) | -1 (-1 to -1) | -3 (-4 to -2) | 2 (1 to 3) | 1 (0 to 1) |
| Central African Republic | CAF (AFRO) | 674 (396 to 826) | 2,446 (1,444 to 2,987) | 0 (0 to 0) | 0 (0 to 0) | 1,790 (1,022 to 2,206) | 476 (263 to 592) |
| China | CHN (WPRO) | 0 (0 to 0) | 0 (0 to 0) | -1,175 (-1,773 to -707) | -4,701 (-7,092 to -2,827) | 0 (0 to 0) | 0 (0 to 0) |
| Ivory Coast | CIV (AFRO) | 1,730 (854 to 2,501) | 8,762 (4,330 to 12,663) | -10 (-13 to -6) | -39 (-52 to -24) | 6,345 (2,985 to 9,298) | 1,660 (750 to 2,456) |
| Cameroon | CMR (AFRO) | 2,417 (1,726 to 2,903) | 8,701 (6,222 to 10,451) | 0 (0 to 0) | 0 (0 to 0) | 6,305 (4,334 to 7,649) | 1,668 (1,110 to 2,041) |
| Democratic Republic of the Congo | COD (AFRO) | 5,923 (4,385 to 7,898) | 21,330 (15,815 to 28,414) | 0 (0 to 0) | 0 (0 to 0) | 15,510 (11,159 to 20,862) | 4,137 (2,885 to 5,612) |
| Congo | COG (AFRO) | 530 (351 to 665) | 1,910 (1,268 to 2,393) | 0 (0 to 0) | 0 (0 to 0) | 1,375 (883 to 1,754) | 358 (223 to 465) |
| Colombia | COL (PAHO) | 0 (0 to 0) | 17 (12 to 26) | -2 (-3 to -1) | -8 (-10 to -6) | 11 (8 to 17) | 3 (2 to 4) |
| Comoros | COM (AFRO) | 22 (14 to 31) | 80 (52 to 113) | 0 (0 to 0) | 0 (0 to 0) | 55 (35 to 79) | 14 (9 to 20) |
| Cape Verde | CPV (AFRO) | 0 (0 to 0) | 0 (0 to 0) | -2 (-3 to -1) | -9 (-11 to -6) | 0 (0 to 0) | 0 (0 to 0) |
| Cuba | CUB (PAHO) | 0 (0 to 0) | 0 (0 to 0) | -2 (-2 to -1) | -7 (-9 to -4) | 0 (0 to 0) | 0 (0 to 0) |
| Djibouti | DJI (EMRO) | 0 (0 to 0) | 0 (0 to 0) | -2 (-2 to -1) | -6 (-8 to -4) | 0 (0 to 0) | 0 (0 to 0) |
| Algeria | DZA (AFRO) | 0 (0 to 0) | 0 (0 to 0) | -31 (-40 to -18) | -123 (-159 to -70) | 0 (0 to 0) | 0 (0 to 0) |
| Ecuador | ECU (PAHO) | 0 (0 to 0) | 190 (121 to 250) | -8 (-11 to -6) | -34 (-42 to -25) | 133 (81 to 177) | 34 (20 to 45) |
| Egypt | EGY (EMRO) | 0 (0 to 0) | 0 (0 to 0) | -17 (-21 to -14) | -70 (-83 to -57) | 0 (0 to 0) | 0 (0 to 0) |
| Eritrea | ERI (AFRO) | 82 (50 to 115) | 294 (178 to 416) | 0 (0 to 0) | 0 (0 to 0) | 205 (118 to 300) | 52 (29 to 78) |

| Country | ISO country code (WHO region) | HepB-BD scale-up (≥25%) | HepB-BD scale-up (≥90%) | HepB-BD disruptions (5%) | HepB-BD disruptions (20%) | delayed HepB-BD scale-up (2023 to 2030) | delayed HepB-BD scale-up (2025 to 2040) |
|---|---|---|---|---|---|---|---|
| Ethiopia | ETH (AFRO) | 4,599 (2,336 to 7,151) | 16,642 (8,460 to 25,865) | 0 (0 to 0) | 0 (0 to 0) | 11,680 (5,874 to 18,411) | 2,965 (1,478 to 4,734) |
| Fiji | FJI (WPRO) | 0 (0 to 0) | 0 (0 to 0) | -1 (-2 to -1) | -5 (-6 to -3) | 0 (0 to 0) | 0 (0 to 0) |
| Micronesia | FSM (WPRO) | 0 (0 to 0) | 4 (1 to 5) | 0 (0 to 0) | -1 (-1 to 0) | 2 (1 to 3) | 1 (0 to 1) |
| Georgia | GEO (EURO) | 0 (0 to 0) | 0 (0 to 0) | -4 (-5 to -2) | -14 (-19 to -9) | 0 (0 to 0) | 0 (0 to 0) |
| Ghana | GHA (AFRO) | 2,806 (2,235 to 3,241) | 10,097 (8,043 to 11,660) | 0 (0 to 0) | 0 (0 to 0) | 7,315 (5,728 to 8,485) | 1,902 (1,469 to 2,212) |
| Guinea | GIN (AFRO) | 3,983 (2,955 to 5,002) | 14,457 (10,764 to 18,091) | 0 (0 to 0) | 0 (0 to 0) | 10,480 (7,676 to 13,265) | 2,783 (2,005 to 3,538) |
| Gambia | GMB (AFRO) | 120 (48 to 222) | 433 (174 to 801) | 0 (0 to 0) | 0 (0 to 0) | 305 (115 to 586) | 78 (28 to 154) |
| Guinea-Bissau | GNB (AFRO) | 221 (159 to 285) | 797 (574 to 1,026) | 0 (0 to 0) | 0 (0 to 0) | 578 (408 to 750) | 152 (105 to 197) |
| Guatemala | GTM (PAHO) | 0 (0 to 0) | 85 (76 to 98) | -1 (-1 to -1) | -5 (-6 to -5) | 58 (51 to 67) | 14 (13 to 17) |
| Guyana | GUY (PAHO) | 8 (5 to 10) | 30 (18 to 37) | 0 (0 to 0) | 0 (0 to 0) | 21 (12 to 26) | 5 (3 to 7) |
| Honduras | HND (PAHO) | 0 (0 to 0) | 98 (59 to 127) | -6 (-8 to -5) | -26 (-31 to -18) | 67 (39 to 88) | 17 (10 to 22) |
| Haiti | HTI (PAHO) | 222 (166 to 276) | 800 (597 to 995) | 0 (0 to 0) | 0 (0 to 0) | 575 (428 to 716) | 152 (112 to 189) |
| Indonesia | IDN (SEARO) | 0 (0 to 0) | 1,473 (1,140 to 2,014) | -294 (-382 to -236) | -1,174 (-1,528 to -944) | 997 (764 to 1,380) | 249 (189 to 347) |
| India | IND (SEARO) | 0 (0 to 0) | 24,707 (16,090 to 33,817) | -430 (-574 to -295) | -1,722 (-2,295 to -1,179) | 17,577 (11,302 to 24,238) | 4,497 (2,866 to 6,243) |
| Iran | IRN (EMRO) | 0 (0 to 0) | 0 (0 to 0) | -32 (-46 to -13) | -127 (-184 to -50) | 0 (0 to 0) | 0 (0 to 0) |
| Iraq | IRQ (EMRO) | 0 (0 to 0) | 1,254 (876 to 1,599) | -13 (-17 to -10) | -53 (-66 to -40) | 867 (597 to 1,109) | 219 (150 to 281) |
| Jamaica | JAM (PAHO) | 7 (4 to 10) | 26 (15 to 34) | 0 (0 to 0) | 0 (0 to 0) | 18 (10 to 24) | 4 (2 to 6) |
| Jordan | JOR (EMRO) | 15 (11 to 20) | 54 (38 to 71) | 0 (0 to 0) | 0 (0 to 0) | 39 (26 to 51) | 10 (7 to 13) |
| Kenya | KEN (AFRO) | 145 (127 to 167) | 522 (458 to 600) | 0 (0 to 0) | 0 (0 to 0) | 342 (299 to 396) | 83 (72 to 96) |
| Kyrgyzstan | KGZ (EURO) | 0 (0 to 0) | 0 (0 to 0) | -11 (-11 to -10) | -43 (-45 to -40) | 0 (0 to 0) | 0 (0 to 0) |
| Cambodia | KHM (WPRO) | 0 (0 to 0) | 61 (42 to 77) | -33 (-40 to -25) | -132 (-160 to -101) | 42 (28 to 53) | 10 (7 to 13) |
| Kiribati | KIR (WPRO) | 0 (0 to 0) | 0 (0 to 0) | 0 (0 to 0) | -1 (-1 to -1) | 0 (0 to 0) | 0 (0 to 0) |
| Laos | LAO (WPRO) | 0 (0 to 0) | 361 (292 to 421) | -6 (-7 to -5) | -25 (-29 to -21) | 257 (202 to 301) | 67 (52 to 79) |
| Liberia | LBR (AFRO) | 1,185 (793 to 1,485) | 4,283 (2,874 to 5,348) | 0 (0 to 0) | 0 (0 to 0) | 3,081 (2,019 to 3,897) | 803 (515 to 1,028) |
| Sri Lanka | LKA (SEARO) | 135 (99 to 173) | 486 (356 to 624) | 0 (0 to 0) | 0 (0 to 0) | 330 (239 to 434) | 81 (58 to 109) |
| Lesotho | LSO (AFRO) | 51 (31 to 70) | 183 (111 to 251) | 0 (0 to 0) | 0 (0 to 0) | 128 (73 to 180) | 33 (18 to 47) |
| Morocco | MAR (EMRO) | 0 (0 to 0) | 89 (76 to 101) | -1 (-1 to -1) | -4 (-5 to -4) | 60 (51 to 69) | 15 (12 to 17) |
| Moldova | MDA (EURO) | 0 (0 to 0) | 0 (0 to 0) | -8 (-9 to -6) | -32 (-38 to -23) | 0 (0 to 0) | 0 (0 to 0) |
| Madagascar | MDG (AFRO) | 2,328 (2,177 to 2,469) | 8,379 (7,836 to 8,888) | 0 (0 to 0) | 0 (0 to 0) | 6,130 (5,715 to 6,521) | 1,611 (1,500 to 1,718) |
| Marshall Islands | MHL (WPRO) | 0 (0 to 0) | 0 (0 to 0) | 0 (0 to 0) | -1 (-1 to -1) | 0 (0 to 0) | 0 (0 to 0) |
| North Macedonia | MKD (EURO) | 0 (0 to 0) | 0 (0 to 0) | -1 (-1 to -1) | -4 (-6 to -3) | 0 (0 to 0) | 0 (0 to 0) |
| Mali | MLI (AFRO) | 854 (553 to 1,165) | 3,078 (1,991 to 4,199) | 0 (0 to 0) | 0 (0 to 0) | 2,124 (1,346 to 2,946) | 535 (334 to 753) |

| Country | ISO country code (WHO region) | HepB-BD scale-up (≥25%) | HepB-BD scale-up (≥90%) | HepB-BD disruptions (5%) | HepB-BD disruptions (20%) | delayed HepB-BD scale-up (2023 to 2030) | delayed HepB-BD scale-up (2025 to 2040) |
|---|---|---|---|---|---|---|---|
| Myanmar | MMR (SEARO) | 1,253 (785 to 1,722) | 11,450 (7,175 to 15,713) | -30 (-39 to -21) | -120 (-155 to -84) | 8,069 (4,923 to 11,244) | 2,058 (1,232 to 2,899) |
| Mongolia | MNG (WPRO) | 0 (0 to 0) | 0 (0 to 0) | -4 (-7 to -2) | -17 (-29 to -9) | 0 (0 to 0) | 0 (0 to 0) |
| Mozambique | MOZ (AFRO) | 2,682 (2,290 to 3,051) | 9,652 (8,243 to 10,980) | 0 (0 to 0) | 0 (0 to 0) | 7,048 (5,977 to 8,035) | 1,851 (1,561 to 2,116) |
| Mauritania | MRT (AFRO) | 424 (321 to 518) | 1,528 (1,157 to 1,865) | 0 (0 to 0) | 0 (0 to 0) | 1,112 (830 to 1,364) | 293 (216 to 360) |
| Malawi | MWI (AFRO) | 200 (162 to 252) | 720 (584 to 906) | 0 (0 to 0) | 0 (0 to 0) | 481 (383 to 617) | 119 (93 to 154) |
| Namibia | NAM (AFRO) | 0 (0 to 0) | 13 (9 to 16) | -1 (-1 to -1) | -5 (-6 to -4) | 9 (7 to 11) | 2 (2 to 3) |
| Niger | NER (AFRO) | 7,353 (5,454 to 9,047) | 26,507 (19,701 to 32,579) | 0 (0 to 0) | 0 (0 to 0) | 19,456 (14,141 to 24,105) | 5,135 (3,633 to 6,390) |
| Nigeria | NGA (AFRO) | 28,697 (14,962 to 38,411) | 104,890 (54,682 to 140,133) | 0 (0 to 0) | 0 (0 to 0) | 75,588 (39,031 to 101,793) | 19,584 (10,032 to 26,563) |
| Nicaragua | NIC (PAHO) | 7 (5 to 10) | 25 (18 to 35) | 0 (0 to 0) | 0 (0 to 0) | 17 (12 to 23) | 4 (3 to 6) |
| Nepal | NPL (SEARO) | 207 (141 to 264) | 743 (507 to 950) | 0 (0 to 0) | 0 (0 to 0) | 507 (330 to 668) | 126 (79 to 170) |
| Pakistan | PAK (EMRO) | 5,315 (3,248 to 6,296) | 19,131 (11,697 to 22,658) | 0 (0 to 0) | 0 (0 to 0) | 13,715 (7,969 to 16,422) | 3,582 (1,997 to 4,326) |
| Peru | PER (PAHO) | 0 (0 to 0) | 57 (39 to 71) | -8 (-9 to -6) | -32 (-37 to -24) | 39 (26 to 48) | 10 (6 to 12) |
| Philippines | PHL (WPRO) | 0 (0 to 0) | 3,431 (2,674 to 4,129) | -60 (-71 to -48) | -238 (-285 to -194) | 2,277 (1,766 to 2,754) | 559 (433 to 680) |
| Papua New Guinea | PNG (WPRO) | 0 (0 to 0) | 2,117 (1,487 to 2,619) | -9 (-10 to -6) | -34 (-40 to -26) | 1,500 (1,035 to 1,884) | 397 (270 to 503) |
| Korea, North | PRK (SEARO) | 0 (0 to 0) | 0 (0 to 0) | -90 (-123 to -60) | -362 (-493 to -240) | 0 (0 to 0) | 0 (0 to 0) |
| Paraguay | PRY (PAHO) | 64 (40 to 79) | 230 (145 to 285) | 0 (0 to 0) | 0 (0 to 0) | 161 (98 to 202) | 41 (24 to 52) |
| Rwanda | RWA (AFRO) | 199 (166 to 223) | 715 (599 to 803) | 0 (0 to 0) | 0 (0 to 0) | 512 (422 to 578) | 132 (108 to 150) |
| Sudan | SDN (EMRO) | 1,229 (982 to 1,440) | 4,422 (3,536 to 5,183) | 0 (0 to 0) | 0 (0 to 0) | 3,221 (2,533 to 3,793) | 842 (653 to 995) |
| Senegal | SEN (AFRO) | 0 (0 to 0) | 561 (359 to 709) | -50 (-61 to -37) | -201 (-243 to -147) | 406 (252 to 517) | 105 (64 to 135) |
| Solomon Islands | SLB (WPRO) | 0 (0 to 0) | 14 (11 to 19) | -1 (-1 to -1) | -3 (-4 to -2) | 9 (7 to 12) | 2 (2 to 3) |
| Sierra Leone | SLE (AFRO) | 1,055 (753 to 1,330) | 3,810 (2,720 to 4,808) | 0 (0 to 0) | 0 (0 to 0) | 2,637 (1,868 to 3,353) | 659 (464 to 841) |
| El Salvador | SLV (PAHO) | 0 (0 to 0) | 16 (11 to 19) | -1 (-1 to -1) | -4 (-5 to -3) | 11 (7 to 13) | 3 (2 to 3) |
| Somalia | SOM (EMRO) | 2,353 (1,732 to 2,852) | 8,617 (6,373 to 10,412) | 0 (0 to 0) | 0 (0 to 0) | 6,355 (4,661 to 7,715) | 1,689 (1,229 to 2,062) |
| Serbia | SRB (EURO) | 0 (0 to 0) | 0 (0 to 0) | -3 (-4 to -2) | -12 (-17 to -7) | 0 (0 to 0) | 0 (0 to 0) |
| South Sudan | SSD (AFRO) | 1,241 (943 to 1,500) | 4,676 (3,530 to 5,657) | 0 (0 to 0) | 0 (0 to 0) | 3,390 (2,543 to 4,124) | 868 (651 to 1,059) |
| Sao Tome and Principe | STP (AFRO) | 0 (0 to 0) | 0 (0 to 0) | -1 (-1 to 0) | -2 (-3 to -2) | 0 (0 to 0) | 0 (0 to 0) |
| Eswatini | SWZ (AFRO) | 12 (7 to 18) | 44 (26 to 66) | 0 (0 to 0) | 0 (0 to 0) | 30 (17 to 46) | 7 (4 to 12) |
| Syria | SYR (EMRO) | 151 (126 to 182) | 543 (454 to 658) | 0 (0 to 0) | 0 (0 to 0) | 335 (277 to 410) | 79 (65 to 98) |

| Country | ISO country code (WHO region) | HepB-BD scale-up (≥25%) | HepB-BD scale-up (≥90%) | HepB-BD disruptions (5%) | HepB-BD disruptions (20%) | delayed HepB-BD scale-up (2023 to 2030) | delayed HepB-BD scale-up (2025 to 2040) |
|---|---|---|---|---|---|---|---|
| Chad | TCD (AFRO) | 4,440 (3,240 to 5,513) | 16,081 (11,779 to 19,938) | 0 (0 to 0) | 0 (0 to 0) | 11,824 (8,526 to 14,735) | 3,177 (2,257 to 3,984) |
| Togo | TGO (AFRO) | 1,012 (757 to 1,346) | 3,646 (2,729 to 4,843) | 0 (0 to 0) | 0 (0 to 0) | 2,649 (1,946 to 3,555) | 694 (502 to 939) |
| Thailand | THA (SEARO) | 0 (0 to 0) | 0 (0 to 0) | -9 (-11 to -7) | -36 (-44 to -29) | 0 (0 to 0) | 0 (0 to 0) |
| Tajikistan | TJK (EURO) | 0 (0 to 0) | 0 (0 to 0) | -9 (-11 to -7) | -37 (-46 to -29) | 0 (0 to 0) | 0 (0 to 0) |
| Turkmenistan | TKM (EURO) | 0 (0 to 0) | 0 (0 to 0) | -7 (-9 to -6) | -29 (-36 to -22) | 0 (0 to 0) | 0 (0 to 0) |
| Timor-Leste | TLS (SEARO) | 0 (0 to 0) | 40 (31 to 49) | -1 (-2 to -1) | -6 (-7 to -4) | 29 (22 to 36) | 8 (6 to 9) |
| Tonga | TON (WPRO) | 0 (0 to 0) | 0 (0 to 0) | -1 (-1 to 0) | -2 (-3 to -2) | 0 (0 to 0) | 0 (0 to 0) |
| Tunisia | TUN (EMRO) | 0 (0 to 0) | 11 (9 to 13) | -1 (-1 to -1) | -4 (-5 to -3) | 7 (6 to 8) | 2 (1 to 2) |
| Tuvalu | TUV (WPRO) | 0 (0 to 0) | 0 (0 to 0) | 0 (0 to 0) | 0 (0 to 0) | 0 (0 to 0) | 0 (0 to 0) |
| Tanzania | TZA (AFRO) | 1,931 (1,310 to 2,451) | 6,950 (4,715 to 8,821) | 0 (0 to 0) | 0 (0 to 0) | 5,035 (3,301 to 6,479) | 1,317 (845 to 1,710) |
| Uganda | UGA (AFRO) | 2,888 (2,119 to 3,427) | 10,395 (7,627 to 12,332) | 0 (0 to 0) | 0 (0 to 0) | 7,528 (5,391 to 8,990) | 1,958 (1,378 to 2,348) |
| Ukraine | UKR (EURO) | 0 (0 to 0) | 285 (211 to 379) | -8 (-11 to -6) | -33 (-43 to -25) | 190 (139 to 255) | 47 (34 to 63) |
| Uzbekistan | UZB (EURO) | 0 (0 to 0) | 0 (0 to 0) | -62 (-115 to -39) | -249 (-462 to -156) | 0 (0 to 0) | 0 (0 to 0) |
| Venezuela | VEN (PAHO) | 0 (0 to 0) | 288 (160 to 365) | -5 (-6 to -4) | -21 (-26 to -14) | 195 (103 to 249) | 49 (25 to 62) |
| Viet Nam | VNM (WPRO) | 0 (0 to 0) | 1,621 (1,075 to 2,055) | -142 (-174 to -105) | -569 (-698 to -420) | 1,131 (734 to 1,444) | 285 (182 to 366) |
| Vanuatu | VUT (WPRO) | 0 (0 to 0) | 16 (8 to 25) | -2 (-2 to -1) | -7 (-10 to -4) | 12 (6 to 18) | 3 (2 to 5) |
| Samoa | WSM (WPRO) | 0 (0 to 0) | 8 (5 to 10) | 0 (0 to 0) | -1 (-1 to -1) | 5 (4 to 7) | 1 (1 to 2) |
| Yemen | YEM (EMRO) | 718 (484 to 875) | 2,584 (1,744 to 3,150) | 0 (0 to 0) | 0 (0 to 0) | 1,854 (1,216 to 2,278) | 485 (312 to 597) |
| South Africa | ZAF (AFRO) | 1,579 (911 to 2,037) | 5,684 (3,281 to 7,332) | 0 (0 to 0) | 0 (0 to 0) | 4,039 (2,225 to 5,251) | 1,047 (559 to 1,371) |
| Zambia | ZMB (AFRO) | 0 (0 to 0) | 0 (0 to 0) | -33 (-40 to -25) | -132 (-162 to -98) | 0 (0 to 0) | 0 (0 to 0) |
| Zimbabwe | ZWE (AFRO) | 234 (176 to 303) | 843 (633 to 1,092) | 0 (0 to 0) | 0 (0 to 0) | 565 (417 to 741) | 140 (102 to 185) |
| Treatment level such that 80% of treatment-eligible individuals are in treatment in 2030 | | | | | | | |
| Afghanistan | AFG (EMRO) | 0 (0 to 0) | 596 (414 to 781) | -5 (-6 to -4) | -20 (-24 to -15) | 416 (277 to 556) | 106 (68 to 144) |
| Angola | AGO (AFRO) | 1,417 (818 to 1,718) | 5,104 (2,951 to 6,185) | 0 (0 to 0) | 0 (0 to 0) | 3,688 (2,038 to 4,507) | 968 (519 to 1,187) |
| Albania | ALB (EURO) | 0 (0 to 0) | 0 (0 to 0) | 0 (0 to 0) | -1 (-1 to -1) | 0 (0 to 0) | 0 (0 to 0) |
| Armenia | ARM (EURO) | 0 (0 to 0) | 0 (0 to 0) | -1 (-1 to -1) | -3 (-5 to -2) | 0 (0 to 0) | 0 (0 to 0) |
| Azerbaijan | AZE (EURO) | 0 (0 to 0) | 0 (0 to 0) | -6 (-9 to -4) | -25 (-35 to -16) | 0 (0 to 0) | 0 (0 to 0) |
| Burundi | BDI (AFRO) | 96 (79 to 110) | 344 (284 to 395) | 0 (0 to 0) | 0 (0 to 0) | 246 (200 to 284) | 63 (51 to 74) |
| Benin | BEN (AFRO) | 660 (442 to 883) | 2,384 (1,595 to 3,190) | 0 (0 to 0) | 0 (0 to 0) | 1,657 (1,081 to 2,256) | 420 (269 to 578) |
| Burkina Faso | BFA (AFRO) | 324 (219 to 457) | 1,168 (790 to 1,647) | 0 (0 to 0) | 0 (0 to 0) | 800 (521 to 1,159) | 200 (127 to 295) |
| Bangladesh | BGD (SEARO) | 794 (581 to 1,071) | 2,857 (2,091 to 3,854) | 0 (0 to 0) | 0 (0 to 0) | 1,965 (1,425 to 2,690) | 492 (353 to 680) |
| Bosnia and Herzegovina | BIH (EURO) | 3 (2 to 3) | 10 (7 to 12) | 0 (0 to 0) | 0 (0 to 0) | 7 (4 to 8) | 2 (1 to 2) |
| Belarus | BLR (EURO) | 0 (0 to 0) | 0 (0 to 0) | -1 (-1 to -1) | -3 (-4 to -2) | 0 (0 to 0) | 0 (0 to 0) |
| Belize | BLZ (PAHO) | 0 (0 to 0) | 0 (0 to 0) | 0 (0 to 0) | 0 (0 to 0) | 0 (0 to 0) | 0 (0 to 0) |

| Country | ISO country code (WHO region) | HepB-BD scale-up (≥25%) | HepB-BD scale-up (≥90%) | HepB-BD disruptions (5%) | HepB-BD disruptions (20%) | delayed HepB-BD scale-up (2023 to 2030) | delayed HepB-BD scale-up (2025 to 2040) |
|---|---|---|---|---|---|---|---|
| Bolivia | BOL (PAHO) | 25 (13 to 37) | 90 (46 to 132) | 0 (0 to 0) | 0 (0 to 0) | 62 (29 to 92) | 16 (7 to 24) |
| Bhutan | BTN (SEARO) | 0 (0 to 0) | 1 (0 to 1) | 0 (0 to 0) | -1 (-2 to -1) | 1 (0 to 1) | 0 (0 to 0) |
| Central African Republic | CAF (AFRO) | 326 (176 to 408) | 1,179 (637 to 1,475) | 0 (0 to 0) | 0 (0 to 0) | 848 (439 to 1,072) | 221 (111 to 281) |
| China | CHN (WPRO) | 0 (0 to 0) | 0 (0 to 0) | -490 (-782 to -257) | -1,959 (-3,126 to -1,029) | 0 (0 to 0) | 0 (0 to 0) |
| Ivory Coast | CIV (AFRO) | 640 (280 to 876) | 3,243 (1,417 to 4,438) | -4 (-5 to -2) | -16 (-20 to -10) | 2,312 (947 to 3,212) | 597 (235 to 835) |
| Cameroon | CMR (AFRO) | 733 (459 to 946) | 2,641 (1,655 to 3,406) | 0 (0 to 0) | 0 (0 to 0) | 1,869 (1,107 to 2,446) | 483 (275 to 637) |
| Democratic Republic of the Congo | COD (AFRO) | 1,923 (1,349 to 2,458) | 6,925 (4,860 to 8,848) | 0 (0 to 0) | 0 (0 to 0) | 4,916 (3,355 to 6,350) | 1,276 (854 to 1,661) |
| Congo | COG (AFRO) | 224 (129 to 293) | 807 (464 to 1,055) | 0 (0 to 0) | 0 (0 to 0) | 571 (314 to 759) | 147 (78 to 197) |
| Colombia | COL (PAHO) | 0 (0 to 0) | 6 (4 to 11) | -1 (-1 to -1) | -3 (-5 to -2) | 4 (2 to 7) | 1 (1 to 2) |
| Comoros | COM (AFRO) | 10 (7 to 13) | 36 (26 to 48) | 0 (0 to 0) | 0 (0 to 0) | 24 (17 to 33) | 6 (4 to 8) |
| Cape Verde | CPV (AFRO) | 0 (0 to 0) | 0 (0 to 0) | -1 (-1 to -1) | -4 (-5 to -2) | 0 (0 to 0) | 0 (0 to 0) |
| Cuba | CUB (PAHO) | 0 (0 to 0) | 0 (0 to 0) | -1 (-1 to 0) | -3 (-4 to -1) | 0 (0 to 0) | 0 (0 to 0) |
| Djibouti | DJI (EMRO) | 0 (0 to 0) | 0 (0 to 0) | -1 (-1 to 0) | -3 (-3 to -2) | 0 (0 to 0) | 0 (0 to 0) |
| Algeria | DZA (AFRO) | 0 (0 to 0) | 0 (0 to 0) | -11 (-15 to -6) | -45 (-60 to -24) | 0 (0 to 0) | 0 (0 to 0) |
| Ecuador | ECU (PAHO) | 0 (0 to 0) | 60 (35 to 80) | -3 (-4 to -2) | -13 (-16 to -9) | 41 (23 to 55) | 10 (5 to 14) |
| Egypt | EGY (EMRO) | 0 (0 to 0) | 0 (0 to 0) | -7 (-8 to -5) | -26 (-32 to -21) | 0 (0 to 0) | 0 (0 to 0) |
| Eritrea | ERI (AFRO) | 25 (14 to 38) | 89 (49 to 135) | 0 (0 to 0) | 0 (0 to 0) | 61 (31 to 97) | 15 (7 to 25) |
| Ethiopia | ETH (AFRO) | 2,448 (1,983 to 3,138) | 8,848 (7,167 to 11,340) | 0 (0 to 0) | 0 (0 to 0) | 6,228 (5,023 to 8,045) | 1,588 (1,276 to 2,066) |
| Fiji | FJI (WPRO) | 0 (0 to 0) | 0 (0 to 0) | -1 (-1 to 0) | -2 (-3 to -1) | 0 (0 to 0) | 0 (0 to 0) |
| Micronesia | FSM (WPRO) | 0 (0 to 0) | 1 (1 to 2) | 0 (0 to 0) | 0 (0 to 0) | 1 (0 to 1) | 0 (0 to 0) |
| Georgia | GEO (EURO) | 0 (0 to 0) | 0 (0 to 0) | -2 (-2 to -1) | -6 (-9 to -4) | 0 (0 to 0) | 0 (0 to 0) |
| Ghana | GHA (AFRO) | 986 (749 to 1,193) | 3,548 (2,696 to 4,296) | 0 (0 to 0) | 0 (0 to 0) | 2,531 (1,889 to 3,076) | 651 (479 to 794) |
| Guinea | GIN (AFRO) | 1,468 (1,027 to 1,933) | 5,309 (3,719 to 6,976) | 0 (0 to 0) | 0 (0 to 0) | 3,768 (2,574 to 5,044) | 978 (656 to 1,324) |
| Gambia | GMB (AFRO) | 46 (15 to 91) | 165 (53 to 327) | 0 (0 to 0) | 0 (0 to 0) | 115 (34 to 235) | 29 (8 to 61) |
| Guinea-Bissau | GNB (AFRO) | 67 (45 to 93) | 241 (163 to 335) | 0 (0 to 0) | 0 (0 to 0) | 170 (112 to 241) | 44 (28 to 63) |
| Guatemala | GTM (PAHO) | 0 (0 to 0) | 32 (28 to 38) | -1 (-1 to 0) | -2 (-2 to -2) | 21 (18 to 25) | 5 (4 to 6) |
| Guyana | GUY (PAHO) | 3 (2 to 4) | 12 (7 to 16) | 0 (0 to 0) | 0 (0 to 0) | 8 (5 to 11) | 2 (1 to 3) |
| Honduras | HND (PAHO) | 0 (0 to 0) | 38 (20 to 50) | -3 (-3 to -2) | -11 (-14 to -8) | 26 (13 to 34) | 6 (3 to 9) |
| Haiti | HTI (PAHO) | 86 (59 to 107) | 309 (211 to 385) | 0 (0 to 0) | 0 (0 to 0) | 218 (148 to 272) | 56 (38 to 70) |
| Indonesia | IDN (SEARO) | 0 (0 to 0) | 750 (647 to 858) | -157 (-176 to -141) | -629 (-704 to -563) | 507 (433 to 583) | 127 (107 to 146) |
| India | IND (SEARO) | 0 (0 to 0) | 8,682 (5,106 to 12,332) | -170 (-226 to -115) | -678 (-903 to -459) | 6,085 (3,488 to 8,748) | 1,544 (870 to 2,239) |

| Country | ISO country code (WHO region) | HepB-BD scale-up (≥25%) | HepB-BD scale-up (≥90%) | HepB-BD disruptions (5%) | HepB-BD disruptions (20%) | delayed HepB-BD scale-up (2023 to 2030) | delayed HepB-BD scale-up (2025 to 2040) |
|---|---|---|---|---|---|---|---|
| Iran | IRN (EMRO) | 0 (0 to 0) | 0 (0 to 0) | -13 (-19 to -5) | -54 (-77 to -20) | 0 (0 to 0) | 0 (0 to 0) |
| Iraq | IRQ (EMRO) | 0 (0 to 0) | 523 (368 to 657) | -6 (-8 to -5) | -25 (-30 to -18) | 356 (245 to 448) | 89 (60 to 112) |
| Jamaica | JAM (PAHO) | 6 (3 to 8) | 21 (11 to 28) | 0 (0 to 0) | 0 (0 to 0) | 14 (7 to 19) | 3 (2 to 5) |
| Jordan | JOR (EMRO) | 6 (4 to 8) | 21 (14 to 28) | 0 (0 to 0) | 0 (0 to 0) | 15 (10 to 20) | 4 (2 to 5) |
| Kenya | KEN (AFRO) | 49 (42 to 58) | 175 (150 to 211) | 0 (0 to 0) | 0 (0 to 0) | 113 (96 to 138) | 27 (23 to 34) |
| Kyrgyzstan | KGZ (EURO) | 0 (0 to 0) | 0 (0 to 0) | -4 (-4 to -4) | -16 (-17 to -15) | 0 (0 to 0) | 0 (0 to 0) |
| Cambodia | KHM (WPRO) | 0 (0 to 0) | 18 (11 to 25) | -11 (-14 to -8) | -46 (-57 to -34) | 12 (7 to 17) | 3 (2 to 4) |
| Kiribati | KIR (WPRO) | 0 (0 to 0) | 0 (0 to 0) | 0 (0 to 0) | -1 (-1 to 0) | 0 (0 to 0) | 0 (0 to 0) |
| Laos | LAO (WPRO) | 0 (0 to 0) | 106 (82 to 129) | -2 (-3 to -2) | -9 (-10 to -7) | 73 (55 to 90) | 19 (14 to 23) |
| Liberia | LBR (AFRO) | 445 (309 to 559) | 1,608 (1,117 to 2,016) | 0 (0 to 0) | 0 (0 to 0) | 1,133 (755 to 1,444) | 290 (188 to 374) |
| Sri Lanka | LKA (SEARO) | 43 (31 to 58) | 154 (113 to 208) | 0 (0 to 0) | 0 (0 to 0) | 102 (73 to 142) | 25 (17 to 35) |
| Lesotho | LSO (AFRO) | 21 (11 to 30) | 77 (40 to 109) | 0 (0 to 0) | 0 (0 to 0) | 53 (26 to 77) | 13 (6 to 20) |
| Morocco | MAR (EMRO) | 0 (0 to 0) | 27 (22 to 32) | 0 (0 to 0) | -1 (-2 to -1) | 18 (14 to 21) | 4 (3 to 5) |
| Moldova | MDA (EURO) | 0 (0 to 0) | 0 (0 to 0) | -3 (-4 to -2) | -13 (-16 to -9) | 0 (0 to 0) | 0 (0 to 0) |
| Madagascar | MDG (AFRO) | 829 (776 to 883) | 2,983 (2,794 to 3,180) | 0 (0 to 0) | 0 (0 to 0) | 2,164 (2,022 to 2,311) | 564 (527 to 603) |
| Marshall Islands | MHL (WPRO) | 0 (0 to 0) | 0 (0 to 0) | 0 (0 to 0) | -1 (-1 to 0) | 0 (0 to 0) | 0 (0 to 0) |
| North Macedonia | MKD (EURO) | 0 (0 to 0) | 0 (0 to 0) | -1 (-1 to 0) | -2 (-3 to -2) | 0 (0 to 0) | 0 (0 to 0) |
| Mali | MLI (AFRO) | 313 (183 to 459) | 1,129 (658 to 1,652) | 0 (0 to 0) | 0 (0 to 0) | 771 (439 to 1,145) | 193 (108 to 290) |
| Myanmar | MMR (SEARO) | 442 (264 to 639) | 4,036 (2,413 to 5,830) | -12 (-16 to -9) | -49 (-65 to -34) | 2,778 (1,596 to 4,100) | 698 (390 to 1,044) |
| Mongolia | MNG (WPRO) | 0 (0 to 0) | 0 (0 to 0) | -2 (-3 to -1) | -6 (-11 to -3) | 0 (0 to 0) | 0 (0 to 0) |
| Mozambique | MOZ (AFRO) | 957 (813 to 1,072) | 3,444 (2,927 to 3,860) | 0 (0 to 0) | 0 (0 to 0) | 2,468 (2,078 to 2,775) | 638 (535 to 719) |
| Mauritania | MRT (AFRO) | 134 (92 to 179) | 481 (331 to 644) | 0 (0 to 0) | 0 (0 to 0) | 342 (230 to 465) | 88 (58 to 121) |
| Malawi | MWI (AFRO) | 54 (43 to 72) | 195 (154 to 261) | 0 (0 to 0) | 0 (0 to 0) | 126 (97 to 173) | 30 (23 to 42) |
| Namibia | NAM (AFRO) | 0 (0 to 0) | 5 (3 to 6) | -1 (-1 to 0) | -2 (-3 to -2) | 3 (2 to 4) | 1 (1 to 1) |
| Niger | NER (AFRO) | 2,889 (1,976 to 3,787) | 10,410 (7,126 to 13,644) | 0 (0 to 0) | 0 (0 to 0) | 7,490 (4,993 to 9,930) | 1,943 (1,271 to 2,596) |
| Nigeria | NGA (AFRO) | 13,734 (10,917 to 16,748) | 50,028 (39,731 to 60,934) | 0 (0 to 0) | 0 (0 to 0) | 36,043 (28,463 to 44,286) | 9,355 (7,354 to 11,580) |
| Nicaragua | NIC (PAHO) | 2 (2 to 3) | 9 (6 to 12) | 0 (0 to 0) | 0 (0 to 0) | 6 (3 to 8) | 1 (1 to 2) |
| Nepal | NPL (SEARO) | 62 (39 to 84) | 225 (142 to 301) | 0 (0 to 0) | 0 (0 to 0) | 150 (88 to 207) | 37 (20 to 52) |
| Pakistan | PAK (EMRO) | 1,598 (838 to 1,947) | 5,754 (3,019 to 7,010) | 0 (0 to 0) | 0 (0 to 0) | 4,046 (2,011 to 4,988) | 1,039 (497 to 1,290) |
| Peru | PER (PAHO) | 0 (0 to 0) | 20 (12 to 27) | -3 (-4 to -2) | -13 (-17 to -9) | 13 (8 to 18) | 3 (2 to 4) |
| Philippines | PHL (WPRO) | 0 (0 to 0) | 2,270 (1,741 to 2,729) | -41 (-47 to -35) | -165 (-187 to -140) | 1,505 (1,130 to 1,835) | 371 (275 to 456) |
| Papua New Guinea | PNG (WPRO) | 0 (0 to 0) | 700 (452 to 924) | -3 (-4 to -3) | -14 (-16 to -10) | 483 (302 to 648) | 124 (76 to 168) |
| Korea, North | PRK (SEARO) | 0 (0 to 0) | 0 (0 to 0) | -38 (-52 to -23) | -152 (-207 to -94) | 0 (0 to 0) | 0 (0 to 0) |
| Paraguay | PRY (PAHO) | 28 (16 to 36) | 102 (59 to 130) | 0 (0 to 0) | 0 (0 to 0) | 70 (38 to 91) | 18 (9 to 23) |

| Country | ISO country code (WHO region) | HepB-BD scale-up (≥25%) | HepB-BD scale-up (≥90%) | HepB-BD disruptions (5%) | HepB-BD disruptions (20%) | delayed HepB-BD scale-up (2023 to 2030) | delayed HepB-BD scale-up (2025 to 2040) |
|---|---|---|---|---|---|---|---|
| Rwanda | RWA (AFRO) | 60 (50 to 68) | 217 (180 to 245) | 0 (0 to 0) | 0 (0 to 0) | 152 (123 to 173) | 39 (31 to 44) |
| Sudan | SDN (EMRO) | 415 (313 to 494) | 1,493 (1,128 to 1,778) | 0 (0 to 0) | 0 (0 to 0) | 1,065 (791 to 1,274) | 274 (201 to 329) |
| Senegal | SEN (AFRO) | 0 (0 to 0) | 217 (133 to 281) | -21 (-26 to -15) | -85 (-105 to -59) | 155 (92 to 203) | 40 (23 to 53) |
| Solomon Islands | SLB (WPRO) | 0 (0 to 0) | 10 (7 to 12) | -1 (-1 to 0) | -2 (-3 to -2) | 6 (5 to 8) | 1 (1 to 2) |
| Sierra Leone | SLE (AFRO) | 407 (329 to 505) | 1,467 (1,185 to 1,822) | 0 (0 to 0) | 0 (0 to 0) | 990 (783 to 1,250) | 244 (190 to 312) |
| El Salvador | SLV (PAHO) | 0 (0 to 0) | 5 (3 to 6) | 0 (0 to 0) | -1 (-2 to -1) | 3 (2 to 4) | 1 (1 to 1) |
| Somalia | SOM (EMRO) | 1,818 (1,288 to 2,361) | 6,607 (4,694 to 8,558) | 0 (0 to 0) | 0 (0 to 0) | 4,803 (3,382 to 6,284) | 1,260 (883 to 1,662) |
| Serbia | SRB (EURO) | 0 (0 to 0) | 0 (0 to 0) | -1 (-2 to -1) | -5 (-7 to -3) | 0 (0 to 0) | 0 (0 to 0) |
| South Sudan | SSD (AFRO) | 1,087 (769 to 1,342) | 4,026 (2,827 to 5,004) | 0 (0 to 0) | 0 (0 to 0) | 2,877 (1,994 to 3,624) | 738 (510 to 933) |
| Sao Tome and Principe | STP (AFRO) | 0 (0 to 0) | 0 (0 to 0) | 0 (0 to 0) | -1 (-1 to -1) | 0 (0 to 0) | 0 (0 to 0) |
| Eswatini | SWZ (AFRO) | 4 (2 to 7) | 16 (9 to 25) | 0 (0 to 0) | 0 (0 to 0) | 10 (5 to 17) | 3 (1 to 4) |
| Syria | SYR (EMRO) | 89 (76 to 109) | 320 (274 to 392) | 0 (0 to 0) | 0 (0 to 0) | 195 (164 to 243) | 46 (38 to 58) |
| Chad | TCD (AFRO) | 1,641 (1,086 to 2,066) | 5,927 (3,931 to 7,455) | 0 (0 to 0) | 0 (0 to 0) | 4,265 (2,777 to 5,407) | 1,116 (719 to 1,421) |
| Togo | TGO (AFRO) | 350 (245 to 453) | 1,260 (882 to 1,629) | 0 (0 to 0) | 0 (0 to 0) | 895 (611 to 1,171) | 230 (154 to 303) |
| Thailand | THA (SEARO) | 0 (0 to 0) | 0 (0 to 0) | -3 (-4 to -3) | -14 (-17 to -11) | 0 (0 to 0) | 0 (0 to 0) |
| Tajikistan | TJK (EURO) | 0 (0 to 0) | 0 (0 to 0) | -7 (-8 to -5) | -27 (-34 to -21) | 0 (0 to 0) | 0 (0 to 0) |
| Turkmenistan | TKM (EURO) | 0 (0 to 0) | 0 (0 to 0) | -5 (-6 to -4) | -20 (-25 to -17) | 0 (0 to 0) | 0 (0 to 0) |
| Timor-Leste | TLS (SEARO) | 0 (0 to 0) | 12 (9 to 15) | -1 (-1 to 0) | -2 (-2 to -2) | 8 (6 to 11) | 2 (2 to 3) |
| Tonga | TON (WPRO) | 0 (0 to 0) | 0 (0 to 0) | 0 (-1 to 0) | -2 (-2 to -2) | 0 (0 to 0) | 0 (0 to 0) |
| Tunisia | TUN (EMRO) | 0 (0 to 0) | 12 (10 to 13) | -1 (-1 to -1) | -4 (-4 to -4) | 7 (6 to 8) | 2 (1 to 2) |
| Tuvalu | TUV (WPRO) | 0 (0 to 0) | 0 (0 to 0) | 0 (0 to 0) | 0 (0 to 0) | 0 (0 to 0) | 0 (0 to 0) |
| Tanzania | TZA (AFRO) | 600 (353 to 803) | 2,159 (1,272 to 2,889) | 0 (0 to 0) | 0 (0 to 0) | 1,526 (857 to 2,073) | 391 (214 to 537) |
| Uganda | UGA (AFRO) | 1,022 (723 to 1,237) | 3,678 (2,602 to 4,454) | 0 (0 to 0) | 0 (0 to 0) | 2,630 (1,815 to 3,213) | 678 (460 to 833) |
| Ukraine | UKR (EURO) | 0 (0 to 0) | 107 (69 to 142) | -4 (-4 to -3) | -14 (-18 to -10) | 69 (43 to 93) | 17 (10 to 23) |
| Uzbekistan | UZB (EURO) | 0 (0 to 0) | 0 (0 to 0) | -48 (-78 to -32) | -192 (-311 to -126) | 0 (0 to 0) | 0 (0 to 0) |
| Venezuela | VEN (PAHO) | 0 (0 to 0) | 128 (66 to 161) | -3 (-3 to -2) | -10 (-12 to -7) | 85 (41 to 108) | 21 (10 to 27) |
| Viet Nam | VNM (WPRO) | 0 (0 to 0) | 578 (352 to 738) | -58 (-71 to -42) | -232 (-282 to -169) | 394 (232 to 510) | 98 (57 to 128) |
| Vanuatu | VUT (WPRO) | 0 (0 to 0) | 8 (5 to 10) | -1 (-1 to -1) | -4 (-5 to -3) | 5 (3 to 8) | 1 (1 to 2) |
| Samoa | WSM (WPRO) | 0 (0 to 0) | 4 (3 to 4) | 0 (0 to 0) | -1 (-1 to -1) | 2 (2 to 3) | 1 (0 to 1) |
| Yemen | YEM (EMRO) | 234 (143 to 294) | 843 (515 to 1,059) | 0 (0 to 0) | 0 (0 to 0) | 591 (350 to 751) | 151 (88 to 193) |
| South Africa | ZAF (AFRO) | 652 (335 to 864) | 2,347 (1,207 to 3,111) | 0 (0 to 0) | 0 (0 to 0) | 1,633 (795 to 2,196) | 416 (196 to 563) |
| Zambia | ZMB (AFRO) | 0 (0 to 0) | 0 (0 to 0) | -13 (-17 to -9) | -51 (-66 to -35) | 0 (0 to 0) | 0 (0 to 0) |
| Zimbabwe | ZWE (AFRO) | 81 (63 to 109) | 293 (226 to 392) | 0 (0 to 0) | 0 (0 to 0) | 192 (145 to 262) | 47 (35 to 65) |

**Supplementary Table 6** Median year of elimination of HBV (year that HBsAg prevalence in five year olds falls below 0.1%) in selected scenarios for each country modelled, with 95% credibility intervals in parentheses. Two target values are presented for the HepB-BD scale-up scenario i.e. timely HepB-BD expansion to ≥25% and ≥90% by 2030. The six WHO regions AFRO, EMRO, EURO, PAHO, SEARO and WPRO are shown in Supplementary Fig. 1. HBsAg: hepatitis B surface antigen; HBV: hepatitis B virus; HepB3: infant HBV vaccine series; timely HepB-BD: timely birth dose; ISO: International Organization for Standardization; WHO: World Health Organization.

| Country | ISO country code (WHO region) | Status quo HepB3 & HepB-BD | HepB-BD scale-up (≥25%) | HepB-BD scale-up (≥90%) | HepB-BD disruptions (5%) | HepB-BD disruptions (20%) | delayed HepB-BD scale-up (2023 to 2030) | delayed HepB-BD scale-up (2025 to 2040) |
|---|---|---|---|---|---|---|---|---|
| Default settings | | | | | | | | |
| Afghanistan | AFG (EMRO) | 2056 (2044 to 2072) | 2056 (2044 to 2072) | 2034 (2033 to 2038) | 2056 (2044 to 2072) | 2056 (2044 to 2072) | 2034 (2034 to 2038) | 2041 (2038 to 2043) |
| Angola | AGO (AFRO) | after 2100 (2094 to after 2100) | after 2100 (2081 to after 2100) | 2060 (2053 to 2066) | after 2100 (2094 to after 2100) | after 2100 (2094 to after 2100) | 2061 (2054 to 2068) | 2066 (2056 to 2073) |
| Albania | ALB (EURO) | 2023 (2009 to 2026) | 2023 (2009 to 2026) | 2023 (2009 to 2026) | 2023 (2009 to 2026) | 2023 (2009 to 2026) | 2023 (2009 to 2026) | 2023 (2009 to 2026) |
| Armenia | ARM (EURO) | 2032 (2018 to 2035) | 2032 (2018 to 2035) | 2032 (2018 to 2035) | 2032 (2018 to 2035) | 2032 (2018 to 2035) | 2032 (2018 to 2035) | 2032 (2018 to 2035) |
| Azerbaijan | AZE (EURO) | 2034 (2029 to 2039) | 2034 (2029 to 2039) | 2034 (2029 to 2039) | 2034 (2029 to 2039) | 2034 (2029 to 2039) | 2034 (2029 to 2039) | 2034 (2029 to 2039) |
| Burundi | BDI (AFRO) | 2070 (2052 to 2098) | 2057 (2046 to 2069) | 2033 (2032 to 2033) | 2070 (2052 to 2098) | 2070 (2052 to 2098) | 2033 (2033 to 2034) | 2040 (2038 to 2041) |
| Benin | BEN (AFRO) | after 2100 (2088 to after 2100) | 2093 (2078 to after 2100) | 2059 (2055 to 2062) | after 2100 (2088 to after 2100) | after 2100 (2088 to after 2100) | 2060 (2055 to 2063) | 2063 (2057 to 2068) |
| Burkina Faso | BFA (AFRO) | 2055 (2047 to 2070) | 2050 (2045 to 2062) | 2035 (2034 to 2038) | 2055 (2047 to 2070) | 2055 (2047 to 2070) | 2035 (2035 to 2038) | 2042 (2040 to 2043) |
| Bangladesh | BGD (SEARO) | 2089 (2060 to after 2100) | 2069 (2053 to 2090) | 2034 (2034 to 2036) | 2089 (2060 to after 2100) | 2089 (2060 to after 2100) | 2035 (2034 to 2036) | 2043 (2041 to 2044) |
| Bosnia and Herzegovina | BIH (EURO) | 2047 (2040 to 2070) | 2042 (2038 to 2056) | 2032 (2031 to 2033) | 2047 (2040 to 2070) | 2047 (2040 to 2070) | 2033 (2032 to 2034) | 2038 (2037 to 2039) |
| Belarus | BLR (EURO) | 2017 (2010 to 2030) | 2017 (2010 to 2030) | 2017 (2010 to 2030) | 2017 (2010 to 2030) | 2017 (2010 to 2030) | 2017 (2010 to 2030) | 2017 (2010 to 2030) |
| Belize | BLZ (PAHO) | 2016 (2010 to 2020) | 2016 (2010 to 2020) | 2016 (2010 to 2020) | 2016 (2010 to 2020) | 2016 (2010 to 2020) | 2016 (2010 to 2020) | 2016 (2010 to 2020) |
| Bolivia | BOL (PAHO) | 2037 (2031 to 2057) | 2033 (2030 to 2042) | 2029 (2028 to 2030) | 2037 (2031 to 2057) | 2037 (2031 to 2057) | 2031 (2029 to 2031) | 2033 (2031 to 2036) |
| Bhutan | BTN (SEARO) | 2046 (2036 to 2050) | 2046 (2036 to 2050) | 2045 (2034 to 2048) | 2047 (2036 to 2050) | 2047 (2036 to 2050) | 2045 (2034 to 2048) | 2045 (2035 to 2048) |

| Country | ISO country code (WHO region) | Status quo HepB3 & HepB-BD | HepB-BD scale-up (≥25%) | HepB-BD scale-up (≥90%) | HepB-BD disruptions (5%) | HepB-BD disruptions (20%) | delayed HepB-BD scale-up (2023 to 2030) | delayed HepB-BD scale-up (2025 to 2040) |
|---|---|---|---|---|---|---|---|---|
| Central African Republic | CAF (AFRO) | after 2100 (after 2100 to after 2100) | after 2100 (after 2100 to after 2100) | 2072 (2064 to 2083) | after 2100 (after 2100 to after 2100) | after 2100 (after 2100 to after 2100) | 2073 (2065 to 2085) | 2078 (2069 to 2090) |
| China | CHN (WPRO) | 2026 (2021 to 2030) | 2026 (2021 to 2030) | 2026 (2021 to 2030) | 2026 (2021 to 2030) | 2027 (2021 to 2030) | 2026 (2021 to 2030) | 2026 (2021 to 2030) |
| Ivory Coast | CIV (AFRO) | after 2100 (2070 to after 2100) | after 2100 (2066 to after 2100) | 2058 (2047 to 2064) | after 2100 (2070 to after 2100) | after 2100 (2070 to after 2100) | 2059 (2047 to 2065) | 2063 (2048 to 2070) |
| Cameroon | CMR (AFRO) | after 2100 (2077 to after 2100) | 2100 (2069 to after 2100) | 2053 (2044 to 2061) | after 2100 (2077 to after 2100) | after 2100 (2077 to after 2100) | 2054 (2044 to 2062) | 2057 (2045 to 2067) |
| Democratic Republic of the Congo | COD (AFRO) | after 2100 (2081 to after 2100) | 2098 (2071 to after 2100) | 2052 (2043 to 2060) | after 2100 (2081 to after 2100) | after 2100 (2081 to after 2100) | 2054 (2044 to 2060) | 2057 (2046 to 2065) |
| Congo | COG (AFRO) | after 2100 (2080 to after 2100) | after 2100 (2073 to after 2100) | 2060 (2052 to 2069) | after 2100 (2080 to after 2100) | after 2100 (2080 to after 2100) | 2061 (2053 to 2070) | 2066 (2054 to 2075) |
| Colombia | COL (PAHO) | 2016 (2009 to 2018) | 2016 (2009 to 2018) | 2016 (2009 to 2018) | 2016 (2009 to 2018) | 2016 (2009 to 2018) | 2016 (2009 to 2018) | 2016 (2009 to 2018) |
| Comoros | COM (AFRO) | 2074 (2063 to 2097) | 2067 (2058 to 2083) | 2045 (2040 to 2053) | 2074 (2063 to 2097) | 2074 (2063 to 2097) | 2045 (2040 to 2054) | 2046 (2044 to 2056) |
| Cape Verde | CPV (AFRO) | 2044 (2040 to 2050) | 2044 (2040 to 2050) | 2044 (2040 to 2050) | 2044 (2040 to 2050) | 2045 (2040 to 2051) | 2044 (2040 to 2050) | 2044 (2040 to 2050) |
| Cuba | CUB (PAHO) | 2005 (2005 to 2005) | 2005 (2005 to 2005) | 2005 (2005 to 2005) | 2005 (2005 to 2005) | 2005 (2005 to 2005) | 2005 (2005 to 2005) | 2005 (2005 to 2005) |
| Djibouti | DJI (EMRO) | 2046 (2040 to 2050) | 2046 (2040 to 2050) | 2046 (2040 to 2050) | 2046 (2040 to 2050) | 2046 (2040 to 2050) | 2046 (2040 to 2050) | 2046 (2040 to 2050) |
| Algeria | DZA (AFRO) | 2009 (2009 to 2011) | 2009 (2009 to 2011) | 2009 (2009 to 2011) | 2009 (2009 to 2011) | 2009 (2009 to 2011) | 2009 (2009 to 2011) | 2009 (2009 to 2011) |
| Ecuador | ECU (PAHO) | 2038 (2033 to 2043) | 2038 (2033 to 2043) | 2031 (2029 to 2034) | 2038 (2033 to 2043) | 2038 (2033 to 2043) | 2032 (2030 to 2034) | 2035 (2032 to 2038) |
| Egypt | EGY (EMRO) | 2022 (2022 to 2022) | 2022 (2022 to 2022) | 2022 (2022 to 2022) | 2022 (2022 to 2022) | 2022 (2022 to 2022) | 2022 (2022 to 2022) | 2022 (2022 to 2022) |
| Eritrea | ERI (AFRO) | 2061 (2042 to 2100) | 2055 (2040 to 2078) | 2034 (2033 to 2037) | 2061 (2042 to 2100) | 2061 (2042 to 2100) | 2034 (2034 to 2037) | 2041 (2038 to 2044) |
| Ethiopia | ETH (AFRO) | 2080 (2073 to 2091) | 2072 (2066 to 2080) | 2048 (2045 to 2051) | 2080 (2073 to 2091) | 2080 (2073 to 2091) | 2048 (2045 to 2051) | 2049 (2046 to 2052) |
| Fiji | FJI (WPRO) | 2032 (2025 to 2037) | 2032 (2025 to 2037) | 2032 (2025 to 2037) | 2032 (2026 to 2037) | 2032 (2026 to 2037) | 2032 (2025 to 2037) | 2032 (2025 to 2037) |

| Country | ISO country code (WHO region) | Status quo HepB3 & HepB-BD | HepB-BD scale-up (≥25%) | HepB-BD scale-up (≥90%) | HepB-BD disruptions (5%) | HepB-BD disruptions (20%) | delayed HepB-BD scale-up (2023 to 2030) | delayed HepB-BD scale-up (2025 to 2040) |
|---|---|---|---|---|---|---|---|---|
| Micronesia | FSM (WPRO) | 2062 (2044 to 2071) | 2062 (2044 to 2071) | 2042 (2037 to 2058) | 2063 (2044 to 2071) | 2063 (2044 to 2071) | 2042 (2037 to 2059) | 2043 (2040 to 2061) |
| Georgia | GEO (EURO) | 2038 (2033 to 2042) | 2038 (2033 to 2042) | 2038 (2033 to 2042) | 2038 (2033 to 2042) | 2038 (2033 to 2042) | 2038 (2033 to 2042) | 2038 (2033 to 2042) |
| Ghana | GHA (AFRO) | after 2100 (2097 to after 2100) | after 2100 (2081 to after 2100) | 2055 (2047 to 2060) | after 2100 (2097 to after 2100) | after 2100 (2097 to after 2100) | 2056 (2047 to 2062) | 2060 (2048 to 2066) |
| Guinea | GIN (AFRO) | after 2100 (after 2100 to after 2100) | after 2100 (after 2100 to after 2100) | 2075 (2066 to 2090) | after 2100 (after 2100 to after 2100) | after 2100 (after 2100 to after 2100) | 2076 (2067 to 2091) | 2080 (2072 to 2095) |
| Gambia | GMB (AFRO) | 2067 (2045 to after 2100) | 2060 (2040 to 2094) | 2034 (2033 to 2042) | 2067 (2045 to after 2100) | 2067 (2045 to after 2100) | 2035 (2033 to 2042) | 2042 (2038 to 2045) |
| Guinea-Bissau | GNB (AFRO) | after 2100 (2078 to after 2100) | after 2100 (2071 to after 2100) | 2056 (2048 to 2063) | after 2100 (2078 to after 2100) | after 2100 (2078 to after 2100) | 2057 (2048 to 2064) | 2061 (2049 to 2070) |
| Guatemala | GTM (PAHO) | 2012 (2011 to 2012) | 2012 (2011 to 2012) | 2012 (2011 to 2012) | 2012 (2011 to 2012) | 2012 (2011 to 2012) | 2012 (2011 to 2012) | 2012 (2011 to 2012) |
| Guyana | GUY (PAHO) | after 2100 (2065 to after 2100) | 2083 (2058 to after 2100) | 2040 (2035 to 2050) | after 2100 (2065 to after 2100) | after 2100 (2065 to after 2100) | 2040 (2035 to 2052) | 2045 (2042 to 2054) |
| Honduras | HND (PAHO) | 2042 (2036 to 2047) | 2042 (2036 to 2047) | 2035 (2032 to 2039) | 2042 (2036 to 2047) | 2042 (2036 to 2047) | 2035 (2032 to 2039) | 2039 (2035 to 2041) |
| Haiti | HTI (PAHO) | after 2100 (2087 to after 2100) | 2098 (2073 to after 2100) | 2047 (2037 to 2055) | after 2100 (2087 to after 2100) | after 2100 (2087 to after 2100) | 2048 (2037 to 2057) | 2049 (2044 to 2061) |
| Indonesia | IDN (SEARO) | 2054 (2051 to 2057) | 2054 (2051 to 2057) | 2051 (2048 to 2055) | 2054 (2051 to 2057) | 2054 (2051 to 2057) | 2051 (2048 to 2055) | 2051 (2048 to 2055) |
| India | IND (SEARO) | 2046 (2042 to 2051) | 2046 (2042 to 2051) | 2036 (2033 to 2040) | 2046 (2042 to 2051) | 2046 (2042 to 2051) | 2036 (2034 to 2040) | 2041 (2038 to 2043) |
| Iran | IRN (EMRO) | 2005 (2005 to 2005) | 2005 (2005 to 2005) | 2005 (2005 to 2005) | 2005 (2005 to 2005) | 2005 (2005 to 2005) | 2005 (2005 to 2005) | 2005 (2005 to 2005) |
| Iraq | IRQ (EMRO) | 2055 (2046 to 2068) | 2055 (2046 to 2068) | 2034 (2033 to 2035) | 2055 (2046 to 2068) | 2055 (2046 to 2068) | 2034 (2033 to 2035) | 2039 (2037 to 2042) |
| Jamaica | JAM (PAHO) | 2074 (2046 to after 2100) | 2063 (2044 to 2091) | 2034 (2034 to 2035) | 2074 (2046 to after 2100) | 2074 (2046 to after 2100) | 2035 (2034 to 2036) | 2042 (2039 to 2044) |
| Jordan | JOR (EMRO) | 1997 (1988 to 2000) | 1997 (1988 to 2000) | 1997 (1988 to 2000) | 1997 (1988 to 2000) | 1997 (1988 to 2000) | 1997 (1988 to 2000) | 1997 (1988 to 2000) |
| Kenya | KEN (AFRO) | 2022 (2013 to 2023) | 2022 (2013 to 2023) | 2022 (2013 to 2023) | 2022 (2013 to 2023) | 2022 (2013 to 2023) | 2022 (2013 to 2023) | 2022 (2013 to 2023) |
| Kyrgyzstan | KGZ (EURO) | 2019 (2007 to 2029) | 2019 (2007 to 2029) | 2019 (2007 to 2029) | 2019 (2007 to 2029) | 2019 (2007 to 2029) | 2019 (2007 to 2029) | 2019 (2007 to 2029) |

| Country | ISO country code (WHO region) | Status quo HepB3 & HepB-BD | HepB-BD scale-up (≥25%) | HepB-BD scale-up (≥90%) | HepB-BD disruptions (5%) | HepB-BD disruptions (20%) | delayed HepB-BD scale-up (2023 to 2030) | delayed HepB-BD scale-up (2025 to 2040) |
|---|---|---|---|---|---|---|---|---|
| Cambodia | KHM (WPRO) | 2040 (2037 to 2042) | 2040 (2037 to 2042) | 2039 (2036 to 2042) | 2040 (2037 to 2042) | 2040 (2037 to 2042) | 2039 (2036 to 2042) | 2039 (2037 to 2042) |
| Kiribati | KIR (WPRO) | 2041 (2032 to 2051) | 2041 (2032 to 2051) | 2041 (2032 to 2051) | 2041 (2032 to 2051) | 2041 (2032 to 2052) | 2041 (2032 to 2051) | 2041 (2032 to 2051) |
| Laos | LAO (WPRO) | 2061 (2052 to 2074) | 2061 (2052 to 2074) | 2041 (2035 to 2048) | 2061 (2052 to 2074) | 2061 (2052 to 2074) | 2041 (2035 to 2048) | 2044 (2042 to 2048) |
| Liberia | LBR (AFRO) | after 2100 (after 2100 to after 2100) | after 2100 (2098 to after 2100) | 2069 (2063 to 2079) | after 2100 (after 2100 to after 2100) | after 2100 (after 2100 to after 2100) | 2070 (2064 to 2080) | 2075 (2067 to 2084) |
| Sri Lanka | LKA (SEARO) | 2057 (2043 to 2078) | 2049 (2041 to 2064) | 2034 (2033 to 2037) | 2057 (2043 to 2078) | 2057 (2043 to 2078) | 2034 (2034 to 2037) | 2040 (2038 to 2042) |
| Lesotho | LSO (AFRO) | 2094 (2060 to after 2100) | 2079 (2055 to after 2100) | 2047 (2038 to 2055) | 2094 (2060 to after 2100) | 2094 (2060 to after 2100) | 2048 (2038 to 2056) | 2049 (2043 to 2060) |
| Morocco | MAR (EMRO) | 2005 (2005 to 2006) | 2005 (2005 to 2006) | 2005 (2005 to 2006) | 2005 (2005 to 2006) | 2005 (2005 to 2006) | 2005 (2005 to 2006) | 2005 (2005 to 2006) |
| Moldova | MDA (EURO) | 2043 (2040 to 2052) | 2043 (2040 to 2052) | 2043 (2040 to 2052) | 2043 (2040 to 2053) | 2043 (2040 to 2054) | 2043 (2040 to 2052) | 2043 (2040 to 2052) |
| Madagascar | MDG (AFRO) | after 2100 (after 2100 to after 2100) | after 2100 (after 2100 to after 2100) | 2054 (2051 to 2056) | after 2100 (after 2100 to after 2100) | after 2100 (after 2100 to after 2100) | 2055 (2053 to 2057) | 2059 (2056 to 2062) |
| Marshall Islands | MHL (WPRO) | 2044 (2039 to 2059) | 2044 (2039 to 2059) | 2044 (2039 to 2059) | 2044 (2039 to 2059) | 2044 (2039 to 2060) | 2044 (2039 to 2059) | 2044 (2039 to 2059) |
| North Macedonia | MKD (EURO) | 2040 (2017 to 2044) | 2040 (2017 to 2044) | 2040 (2017 to 2044) | 2040 (2017 to 2044) | 2040 (2017 to 2044) | 2040 (2017 to 2044) | 2040 (2017 to 2044) |
| Mali | MLI (AFRO) | 2052 (2046 to 2060) | 2048 (2043 to 2055) | 2035 (2034 to 2035) | 2052 (2046 to 2060) | 2052 (2046 to 2060) | 2035 (2034 to 2036) | 2041 (2039 to 2042) |
| Myanmar | MMR (SEARO) | after 2100 (2079 to after 2100) | after 2100 (2077 to after 2100) | 2063 (2053 to 2069) | after 2100 (2079 to after 2100) | after 2100 (2079 to after 2100) | 2063 (2054 to 2069) | 2067 (2056 to 2075) |
| Mongolia | MNG (WPRO) | 2024 (2018 to 2027) | 2024 (2018 to 2027) | 2024 (2018 to 2027) | 2024 (2018 to 2027) | 2024 (2018 to 2027) | 2024 (2018 to 2027) | 2024 (2018 to 2027) |
| Mozambique | MOZ (AFRO) | after 2100 (after 2100 to after 2100) | after 2100 (2090 to after 2100) | 2054 (2049 to 2059) | after 2100 (after 2100 to after 2100) | after 2100 (after 2100 to after 2100) | 2056 (2050 to 2061) | 2060 (2051 to 2066) |
| Mauritania | MRT (AFRO) | after 2100 (2089 to after 2100) | after 2100 (2076 to after 2100) | 2056 (2047 to 2063) | after 2100 (2089 to after 2100) | after 2100 (2089 to after 2100) | 2057 (2047 to 2064) | 2061 (2048 to 2069) |
| Malawi | MWI (AFRO) | 2037 (2035 to 2041) | 2034 (2033 to 2037) | 2031 (2030 to 2031) | 2037 (2035 to 2041) | 2037 (2035 to 2041) | 2032 (2031 to 2032) | 2034 (2033 to 2036) |

| Country | ISO country code (WHO region) | Status quo HepB3 & HepB-BD | HepB-BD scale-up (≥25%) | HepB-BD scale-up (≥90%) | HepB-BD disruptions (5%) | HepB-BD disruptions (20%) | delayed HepB-BD scale-up (2023 to 2030) | delayed HepB-BD scale-up (2025 to 2040) |
|---|---|---|---|---|---|---|---|---|
| Namibia | NAM (AFRO) | 2040 (2036 to 2043) | 2040 (2036 to 2043) | 2035 (2031 to 2041) | 2040 (2036 to 2043) | 2040 (2036 to 2043) | 2035 (2032 to 2041) | 2038 (2034 to 2041) |
| Niger | NER (AFRO) | after 2100 (after 2100 to after 2100) | after 2100 (2094 to after 2100) | 2066 (2060 to 2076) | after 2100 (after 2100 to after 2100) | after 2100 (after 2100 to after 2100) | 2067 (2061 to 2077) | 2072 (2064 to 2081) |
| Nigeria | NGA (AFRO) | after 2100 (after 2100 to after 2100) | after 2100 (after 2100 to after 2100) | 2065 (2063 to 2069) | after 2100 (after 2100 to after 2100) | after 2100 (after 2100 to after 2100) | 2066 (2064 to 2070) | 2071 (2068 to 2074) |
| Nicaragua | NIC (PAHO) | 2003 (2003 to 2004) | 2003 (2003 to 2004) | 2003 (2003 to 2004) | 2003 (2003 to 2004) | 2003 (2003 to 2004) | 2003 (2003 to 2004) | 2003 (2003 to 2004) |
| Nepal | NPL (SEARO) | 2050 (2037 to 2083) | 2041 (2035 to 2059) | 2032 (2031 to 2032) | 2050 (2037 to 2083) | 2050 (2037 to 2083) | 2033 (2032 to 2033) | 2037 (2034 to 2040) |
| Pakistan | PAK (EMRO) | 2086 (2049 to after 2100) | 2066 (2044 to 2088) | 2034 (2033 to 2035) | 2086 (2049 to after 2100) | 2086 (2049 to after 2100) | 2034 (2034 to 2035) | 2042 (2039 to 2044) |
| Peru | PER (PAHO) | 2008 (2008 to 2009) | 2008 (2008 to 2009) | 2008 (2008 to 2009) | 2008 (2008 to 2009) | 2008 (2008 to 2009) | 2008 (2008 to 2009) | 2008 (2008 to 2009) |
| Philippines | PHL (WPRO) | 2066 (2059 to 2073) | 2066 (2059 to 2073) | 2048 (2045 to 2053) | 2066 (2059 to 2073) | 2066 (2059 to 2073) | 2048 (2045 to 2053) | 2049 (2046 to 2054) |
| Papua New Guinea | PNG (WPRO) | after 2100 (after 2100 to after 2100) | after 2100 (after 2100 to after 2100) | 2066 (2059 to 2076) | after 2100 (after 2100 to after 2100) | after 2100 (after 2100 to after 2100) | 2067 (2060 to 2077) | 2071 (2063 to 2081) |
| Korea, North | PRK (SEARO) | 2042 (2039 to 2043) | 2042 (2039 to 2043) | 2042 (2039 to 2043) | 2042 (2039 to 2043) | 2042 (2039 to 2043) | 2042 (2039 to 2043) | 2042 (2039 to 2043) |
| Paraguay | PRY (PAHO) | 2089 (2058 to after 2100) | 2071 (2052 to 2097) | 2035 (2034 to 2043) | 2089 (2058 to after 2100) | 2089 (2058 to after 2100) | 2035 (2034 to 2043) | 2043 (2041 to 2045) |
| Rwanda | RWA (AFRO) | 2056 (2045 to 2071) | 2046 (2039 to 2055) | 2031 (2031 to 2032) | 2056 (2045 to 2071) | 2056 (2045 to 2071) | 2032 (2032 to 2033) | 2037 (2036 to 2039) |
| Sudan | SDN (EMRO) | 2099 (2069 to after 2100) | 2079 (2060 to after 2100) | 2035 (2035 to 2044) | 2099 (2069 to after 2100) | 2099 (2069 to after 2100) | 2035 (2035 to 2044) | 2044 (2043 to 2045) |
| Senegal | SEN (AFRO) | 2050 (2042 to 2055) | 2050 (2042 to 2055) | 2044 (2038 to 2049) | 2050 (2042 to 2055) | 2050 (2042 to 2055) | 2044 (2038 to 2049) | 2044 (2040 to 2049) |
| Solomon Islands | SLB (WPRO) | 2053 (2047 to 2059) | 2053 (2047 to 2059) | 2045 (2038 to 2050) | 2053 (2047 to 2059) | 2053 (2047 to 2059) | 2045 (2038 to 2050) | 2045 (2041 to 2051) |
| Sierra Leone | SLE (AFRO) | 2095 (2082 to after 2100) | 2085 (2076 to 2096) | 2059 (2055 to 2064) | 2095 (2082 to after 2100) | 2095 (2082 to after 2100) | 2060 (2056 to 2065) | 2063 (2058 to 2069) |
| El Salvador | SLV (PAHO) | 2020 (2020 to 2020) | 2020 (2020 to 2020) | 2020 (2020 to 2020) | 2020 (2020 to 2020) | 2020 (2020 to 2020) | 2020 (2020 to 2020) | 2020 (2020 to 2020) |

| Country | ISO country code (WHO region) | Status quo HepB3 & HepB-BD | HepB-BD scale-up (≥25%) | HepB-BD scale-up (≥90%) | HepB-BD disruptions (5%) | HepB-BD disruptions (20%) | delayed HepB-BD scale-up (2023 to 2030) | delayed HepB-BD scale-up (2025 to 2040) |
|---|---|---|---|---|---|---|---|---|
| Somalia | SOM (EMRO) | after 2100 (after 2100 to after 2100) | after 2100 (after 2100 to after 2100) | 2083 (2070 to 2097) | after 2100 (after 2100 to after 2100) | after 2100 (after 2100 to after 2100) | 2084 (2070 to 2098) | 2088 (2076 to after 2100) |
| Serbia | SRB (EURO) | 2018 (2010 to 2036) | 2018 (2010 to 2036) | 2018 (2010 to 2036) | 2018 (2010 to 2036) | 2018 (2010 to 2036) | 2018 (2010 to 2036) | 2018 (2010 to 2036) |
| South Sudan | SSD (AFRO) | after 2100 (after 2100 to after 2100) | after 2100 (after 2100 to after 2100) | 2087 (2074 to after 2100) | after 2100 (after 2100 to after 2100) | after 2100 (after 2100 to after 2100) | 2088 (2075 to after 2100) | 2092 (2078 to after 2100) |
| Sao Tome and Principe | STP (AFRO) | 2040 (2030 to 2046) | 2040 (2030 to 2046) | 2040 (2030 to 2046) | 2040 (2030 to 2046) | 2040 (2030 to 2046) | 2040 (2030 to 2046) | 2040 (2030 to 2046) |
| Eswatini | SWZ (AFRO) | 2051 (2040 to 2068) | 2047 (2037 to 2059) | 2033 (2032 to 2035) | 2051 (2040 to 2068) | 2051 (2040 to 2068) | 2034 (2032 to 2035) | 2039 (2036 to 2042) |
| Syria | SYR (EMRO) | 2068 (2061 to 2082) | 2063 (2057 to 2070) | 2038 (2035 to 2052) | 2068 (2061 to 2082) | 2068 (2061 to 2082) | 2039 (2036 to 2052) | 2044 (2043 to 2053) |
| Chad | TCD (AFRO) | after 2100 (after 2100 to after 2100) | after 2100 (after 2100 to after 2100) | 2075 (2067 to 2089) | after 2100 (after 2100 to after 2100) | after 2100 (after 2100 to after 2100) | 2076 (2068 to 2091) | 2081 (2073 to 2096) |
| Togo | TGO (AFRO) | after 2100 (2092 to after 2100) | after 2100 (2081 to after 2100) | 2060 (2054 to 2068) | after 2100 (2092 to after 2100) | after 2100 (2092 to after 2100) | 2061 (2055 to 2069) | 2065 (2057 to 2074) |
| Thailand | THA (SEARO) | 2022 (2022 to 2022) | 2022 (2022 to 2022) | 2022 (2022 to 2022) | 2022 (2022 to 2022) | 2022 (2022 to 2022) | 2022 (2022 to 2022) | 2022 (2022 to 2022) |
| Tajikistan | TJK (EURO) | 2033 (2029 to 2035) | 2033 (2029 to 2035) | 2033 (2029 to 2035) | 2033 (2029 to 2035) | 2033 (2029 to 2035) | 2033 (2029 to 2035) | 2033 (2029 to 2035) |
| Turkmenistan | TKM (EURO) | 2038 (2034 to 2041) | 2038 (2034 to 2041) | 2038 (2034 to 2041) | 2038 (2034 to 2041) | 2038 (2034 to 2041) | 2038 (2034 to 2041) | 2038 (2034 to 2041) |
| Timor-Leste | TLS (SEARO) | 2051 (2045 to 2057) | 2051 (2045 to 2057) | 2042 (2035 to 2049) | 2051 (2045 to 2058) | 2051 (2045 to 2058) | 2042 (2035 to 2049) | 2044 (2040 to 2049) |
| Tonga | TON (WPRO) | 2052 (2043 to 2063) | 2052 (2043 to 2063) | 2052 (2043 to 2063) | 2052 (2043 to 2063) | 2053 (2043 to 2063) | 2052 (2043 to 2063) | 2052 (2043 to 2063) |
| Tunisia | TUN (EMRO) | 2032 (2013 to 2034) | 2032 (2013 to 2034) | 2030 (2013 to 2031) | 2032 (2013 to 2034) | 2032 (2013 to 2034) | 2030 (2013 to 2031) | 2032 (2013 to 2033) |
| Tuvalu | TUV (WPRO) | 2040 (2035 to 2047) | 2040 (2035 to 2047) | 2040 (2035 to 2047) | 2040 (2035 to 2048) | 2040 (2035 to 2048) | 2040 (2035 to 2047) | 2040 (2035 to 2047) |
| Tanzania | TZA (AFRO) | 2082 (2057 to after 2100) | 2066 (2051 to 2091) | 2034 (2034 to 2039) | 2082 (2057 to after 2100) | 2082 (2057 to after 2100) | 2035 (2034 to 2039) | 2043 (2040 to 2045) |
| Uganda | UGA (AFRO) | after 2100 (2082 to after 2100) | 2096 (2070 to after 2100) | 2048 (2039 to 2053) | after 2100 (2082 to after 2100) | after 2100 (2082 to after 2100) | 2049 (2039 to 2054) | 2050 (2044 to 2058) |

| Country | ISO country code (WHO region) | Status quo HepB3 & HepB-BD | HepB-BD scale-up (≥25%) | HepB-BD scale-up (≥90%) | HepB-BD disruptions (5%) | HepB-BD disruptions (20%) | delayed HepB-BD scale-up (2023 to 2030) | delayed HepB-BD scale-up (2025 to 2040) |
|---|---|---|---|---|---|---|---|---|
| Ukraine | UKR (EURO) | 2045 (2009 to 2061) | 2045 (2009 to 2061) | 2035 (2009 to 2038) | 2045 (2009 to 2061) | 2045 (2009 to 2062) | 2035 (2009 to 2038) | 2039 (2009 to 2042) |
| Uzbekistan | UZB (EURO) | 2037 (2034 to 2039) | 2037 (2034 to 2039) | 2037 (2034 to 2039) | 2037 (2034 to 2039) | 2037 (2034 to 2039) | 2037 (2034 to 2039) | 2037 (2034 to 2039) |
| Venezuela | VEN (PAHO) | 2042 (2035 to 2053) | 2042 (2035 to 2053) | 2032 (2031 to 2033) | 2042 (2035 to 2053) | 2042 (2035 to 2054) | 2033 (2032 to 2034) | 2036 (2034 to 2039) |
| Viet Nam | VNM (WPRO) | 2054 (2046 to 2063) | 2054 (2046 to 2063) | 2049 (2043 to 2054) | 2054 (2046 to 2063) | 2054 (2046 to 2063) | 2049 (2043 to 2054) | 2049 (2044 to 2054) |
| Vanuatu | VUT (WPRO) | 2074 (2061 to 2084) | 2074 (2061 to 2084) | 2064 (2057 to 2074) | 2074 (2061 to 2084) | 2074 (2061 to 2084) | 2064 (2057 to 2074) | 2065 (2058 to 2075) |
| Samoa | WSM (WPRO) | 2075 (2065 to 2095) | 2075 (2065 to 2095) | 2058 (2046 to 2064) | 2075 (2065 to 2095) | 2075 (2066 to 2096) | 2058 (2046 to 2065) | 2060 (2046 to 2067) |
| Yemen | YEM (EMRO) | after 2100 (2070 to after 2100) | 2080 (2060 to after 2100) | 2035 (2034 to 2047) | after 2100 (2070 to after 2100) | after 2100 (2070 to after 2100) | 2035 (2035 to 2048) | 2044 (2042 to 2049) |
| South Africa | ZAF (AFRO) | after 2100 (2072 to after 2100) | after 2100 (2064 to after 2100) | 2053 (2040 to 2059) | after 2100 (2072 to after 2100) | after 2100 (2072 to after 2100) | 2054 (2040 to 2060) | 2057 (2045 to 2065) |
| Zambia | ZMB (AFRO) | 2027 (2024 to 2032) | 2027 (2024 to 2032) | 2027 (2024 to 2032) | 2027 (2024 to 2032) | 2027 (2025 to 2032) | 2027 (2024 to 2032) | 2027 (2024 to 2032) |
| Zimbabwe | ZWE (AFRO) | 2054 (2046 to 2062) | 2050 (2043 to 2057) | 2035 (2034 to 2036) | 2054 (2046 to 2062) | 2054 (2046 to 2062) | 2035 (2034 to 2036) | 2041 (2039 to 2042) |
| HepB3 coverage scaled up to 100% coverage in 2020 | | | | | | | | |
| Afghanistan | AFG (EMRO) | 2050 (2041 to 2065) | 2050 (2041 to 2065) | 2033 (2032 to 2035) | 2050 (2041 to 2065) | 2051 (2041 to 2065) | 2034 (2033 to 2035) | 2040 (2037 to 2042) |
| Angola | AGO (AFRO) | after 2100 (2071 to after 2100) | after 2100 (2064 to after 2100) | 2057 (2047 to 2064) | after 2100 (2071 to after 2100) | after 2100 (2071 to after 2100) | 2058 (2047 to 2065) | 2061 (2047 to 2070) |
| Albania | ALB (EURO) | 2023 (2009 to 2026) | 2023 (2009 to 2026) | 2023 (2009 to 2026) | 2023 (2009 to 2026) | 2023 (2009 to 2026) | 2023 (2009 to 2026) | 2023 (2009 to 2026) |
| Armenia | ARM (EURO) | 2032 (2018 to 2035) | 2032 (2018 to 2035) | 2032 (2018 to 2035) | 2032 (2018 to 2035) | 2032 (2018 to 2035) | 2032 (2018 to 2035) | 2032 (2018 to 2035) |
| Azerbaijan | AZE (EURO) | 2034 (2029 to 2039) | 2034 (2029 to 2039) | 2034 (2029 to 2039) | 2034 (2029 to 2039) | 2034 (2029 to 2039) | 2034 (2029 to 2039) | 2034 (2029 to 2039) |
| Burundi | BDI (AFRO) | 2069 (2051 to 2096) | 2057 (2045 to 2069) | 2033 (2032 to 2033) | 2069 (2051 to 2096) | 2069 (2051 to 2096) | 2033 (2033 to 2034) | 2040 (2038 to 2041) |
| Benin | BEN (AFRO) | 2092 (2076 to after 2100) | 2081 (2069 to after 2100) | 2055 (2050 to 2060) | 2092 (2076 to after 2100) | 2092 (2076 to after 2100) | 2056 (2051 to 2061) | 2058 (2051 to 2065) |
| Burkina Faso | BFA (AFRO) | 2052 (2046 to 2068) | 2048 (2044 to 2060) | 2035 (2034 to 2037) | 2052 (2046 to 2068) | 2052 (2046 to 2068) | 2035 (2034 to 2037) | 2041 (2040 to 2043) |

| Country | ISO country code (WHO region) | Status quo HepB3 & HepB-BD | HepB-BD scale-up (≥25%) | HepB-BD scale-up (≥90%) | HepB-BD disruptions (5%) | HepB-BD disruptions (20%) | delayed HepB-BD scale-up (2023 to 2030) | delayed HepB-BD scale-up (2025 to 2040) |
|---|---|---|---|---|---|---|---|---|
| Bangladesh | BGD (SEARO) | 2088 (2060 to after 2100) | 2069 (2053 to 2090) | 2034 (2034 to 2036) | 2088 (2060 to after 2100) | 2088 (2060 to after 2100) | 2035 (2034 to 2036) | 2043 (2041 to 2044) |
| Bosnia and Herzegovina | BIH (EURO) | 2045 (2039 to 2067) | 2041 (2037 to 2054) | 2032 (2031 to 2033) | 2045 (2039 to 2067) | 2045 (2039 to 2067) | 2033 (2032 to 2033) | 2038 (2036 to 2039) |
| Belarus | BLR (EURO) | 2017 (2010 to 2030) | 2017 (2010 to 2030) | 2017 (2010 to 2030) | 2017 (2010 to 2030) | 2017 (2010 to 2030) | 2017 (2010 to 2030) | 2017 (2010 to 2030) |
| Belize | BLZ (PAHO) | 2016 (2010 to 2020) | 2016 (2010 to 2020) | 2016 (2010 to 2020) | 2016 (2010 to 2020) | 2016 (2010 to 2020) | 2016 (2010 to 2020) | 2016 (2010 to 2020) |
| Bolivia | BOL (PAHO) | 2035 (2029 to 2053) | 2032 (2028 to 2040) | 2029 (2027 to 2030) | 2035 (2029 to 2053) | 2035 (2029 to 2053) | 2030 (2028 to 2031) | 2032 (2029 to 2036) |
| Bhutan | BTN (SEARO) | 2046 (2035 to 2050) | 2046 (2035 to 2050) | 2044 (2034 to 2048) | 2046 (2035 to 2050) | 2046 (2035 to 2050) | 2044 (2034 to 2048) | 2044 (2035 to 2048) |
| Central African Republic | CAF (AFRO) | after 2100 (2080 to after 2100) | after 2100 (2074 to after 2100) | 2066 (2055 to 2074) | after 2100 (2080 to after 2100) | after 2100 (2080 to after 2100) | 2067 (2055 to 2075) | 2072 (2057 to 2080) |
| China | CHN (WPRO) | 2026 (2021 to 2030) | 2026 (2021 to 2030) | 2026 (2021 to 2030) | 2026 (2021 to 2030) | 2026 (2021 to 2030) | 2026 (2021 to 2030) | 2026 (2021 to 2030) |
| Ivory Coast | CIV (AFRO) | after 2100 (2066 to after 2100) | after 2100 (2062 to after 2100) | 2057 (2045 to 2063) | after 2100 (2066 to after 2100) | after 2100 (2066 to after 2100) | 2058 (2045 to 2064) | 2061 (2045 to 2069) |
| Cameroon | CMR (AFRO) | after 2100 (2066 to after 2100) | 2090 (2060 to after 2100) | 2050 (2040 to 2058) | after 2100 (2066 to after 2100) | after 2100 (2066 to after 2100) | 2051 (2040 to 2060) | 2053 (2044 to 2064) |
| Democratic Republic of the Congo | COD (AFRO) | 2099 (2064 to after 2100) | 2082 (2059 to after 2100) | 2049 (2039 to 2056) | 2099 (2064 to after 2100) | 2099 (2064 to after 2100) | 2050 (2039 to 2057) | 2051 (2044 to 2062) |
| Congo | COG (AFRO) | after 2100 (2072 to after 2100) | 2097 (2067 to after 2100) | 2058 (2050 to 2066) | after 2100 (2072 to after 2100) | after 2100 (2072 to after 2100) | 2059 (2050 to 2067) | 2063 (2050 to 2073) |
| Colombia | COL (PAHO) | 2016 (2009 to 2018) | 2016 (2009 to 2018) | 2016 (2009 to 2018) | 2016 (2009 to 2018) | 2016 (2009 to 2018) | 2016 (2009 to 2018) | 2016 (2009 to 2018) |
| Comoros | COM (AFRO) | 2071 (2061 to 2093) | 2065 (2056 to 2080) | 2044 (2039 to 2051) | 2071 (2061 to 2093) | 2071 (2061 to 2093) | 2044 (2039 to 2052) | 2045 (2043 to 2054) |
| Cape Verde | CPV (AFRO) | 2044 (2039 to 2050) | 2044 (2039 to 2050) | 2044 (2039 to 2050) | 2044 (2039 to 2050) | 2044 (2039 to 2050) | 2044 (2039 to 2050) | 2044 (2039 to 2050) |
| Cuba | CUB (PAHO) | 2005 (2005 to 2005) | 2005 (2005 to 2005) | 2005 (2005 to 2005) | 2005 (2005 to 2005) | 2005 (2005 to 2005) | 2005 (2005 to 2005) | 2005 (2005 to 2005) |
| Djibouti | DJI (EMRO) | 2045 (2038 to 2049) | 2045 (2038 to 2049) | 2045 (2038 to 2049) | 2045 (2038 to 2049) | 2045 (2038 to 2049) | 2045 (2038 to 2049) | 2045 (2038 to 2049) |
| Algeria | DZA (AFRO) | 2009 (2009 to 2011) | 2009 (2009 to 2011) | 2009 (2009 to 2011) | 2009 (2009 to 2011) | 2009 (2009 to 2011) | 2009 (2009 to 2011) | 2009 (2009 to 2011) |

| Country | ISO country code (WHO region) | Status quo HepB3 & HepB-BD | HepB-BD scale-up (≥25%) | HepB-BD scale-up (≥90%) | HepB-BD disruptions (5%) | HepB-BD disruptions (20%) | delayed HepB-BD scale-up (2023 to 2030) | delayed HepB-BD scale-up (2025 to 2040) |
|---|---|---|---|---|---|---|---|---|
| Ecuador | ECU (PAHO) | 2037 (2032 to 2042) | 2037 (2032 to 2042) | 2031 (2028 to 2033) | 2037 (2032 to 2042) | 2037 (2032 to 2042) | 2032 (2030 to 2034) | 2034 (2031 to 2038) |
| Egypt | EGY (EMRO) | 2022 (2022 to 2022) | 2022 (2022 to 2022) | 2022 (2022 to 2022) | 2022 (2022 to 2022) | 2022 (2022 to 2022) | 2022 (2022 to 2022) | 2022 (2022 to 2022) |
| Eritrea | ERI (AFRO) | 2060 (2041 to 2098) | 2054 (2039 to 2076) | 2034 (2033 to 2036) | 2060 (2041 to 2098) | 2060 (2041 to 2098) | 2034 (2033 to 2037) | 2041 (2038 to 2044) |
| Ethiopia | ETH (AFRO) | 2067 (2060 to 2077) | 2061 (2056 to 2069) | 2043 (2040 to 2046) | 2067 (2060 to 2077) | 2067 (2060 to 2077) | 2043 (2040 to 2046) | 2045 (2044 to 2046) |
| Fiji | FJI (WPRO) | 2032 (2025 to 2037) | 2032 (2025 to 2037) | 2032 (2025 to 2037) | 2032 (2026 to 2037) | 2032 (2026 to 2037) | 2032 (2025 to 2037) | 2032 (2025 to 2037) |
| Micronesia | FSM (WPRO) | 2060 (2043 to 2070) | 2060 (2043 to 2070) | 2042 (2036 to 2057) | 2060 (2043 to 2070) | 2061 (2043 to 2070) | 2042 (2036 to 2058) | 2043 (2039 to 2059) |
| Georgia | GEO (EURO) | 2037 (2032 to 2041) | 2037 (2032 to 2041) | 2037 (2032 to 2041) | 2037 (2032 to 2041) | 2037 (2032 to 2041) | 2037 (2032 to 2041) | 2037 (2032 to 2041) |
| Ghana | GHA (AFRO) | after 2100 (2096 to after 2100) | after 2100 (2081 to after 2100) | 2055 (2046 to 2060) | after 2100 (2096 to after 2100) | after 2100 (2096 to after 2100) | 2056 (2047 to 2061) | 2060 (2047 to 2066) |
| Guinea | GIN (AFRO) | after 2100 (2086 to after 2100) | after 2100 (2078 to after 2100) | 2067 (2058 to 2077) | after 2100 (2086 to after 2100) | after 2100 (2086 to after 2100) | 2068 (2058 to 2078) | 2073 (2060 to 2083) |
| Gambia | GMB (AFRO) | 2064 (2043 to after 2100) | 2058 (2039 to 2091) | 2034 (2032 to 2040) | 2064 (2043 to after 2100) | 2064 (2043 to after 2100) | 2034 (2033 to 2040) | 2042 (2037 to 2045) |
| Guinea-Bissau | GNB (AFRO) | after 2100 (2073 to after 2100) | 2095 (2066 to after 2100) | 2055 (2046 to 2062) | after 2100 (2073 to after 2100) | after 2100 (2073 to after 2100) | 2056 (2046 to 2064) | 2059 (2047 to 2069) |
| Guatemala | GTM (PAHO) | 2012 (2011 to 2012) | 2012 (2011 to 2012) | 2012 (2011 to 2012) | 2012 (2011 to 2012) | 2012 (2011 to 2012) | 2012 (2011 to 2012) | 2012 (2011 to 2012) |
| Guyana | GUY (PAHO) | after 2100 (2065 to after 2100) | 2083 (2058 to after 2100) | 2040 (2035 to 2050) | after 2100 (2065 to after 2100) | after 2100 (2065 to after 2100) | 2040 (2035 to 2051) | 2045 (2042 to 2054) |
| Honduras | HND (PAHO) | 2041 (2035 to 2046) | 2041 (2035 to 2046) | 2034 (2031 to 2038) | 2041 (2035 to 2046) | 2041 (2035 to 2046) | 2035 (2032 to 2038) | 2038 (2034 to 2041) |
| Haiti | HTI (PAHO) | after 2100 (2068 to after 2100) | 2086 (2059 to after 2100) | 2044 (2035 to 2053) | after 2100 (2068 to after 2100) | after 2100 (2068 to after 2100) | 2044 (2035 to 2054) | 2045 (2043 to 2058) |
| Indonesia | IDN (SEARO) | 2052 (2050 to 2055) | 2052 (2050 to 2055) | 2050 (2047 to 2053) | 2052 (2050 to 2056) | 2052 (2050 to 2056) | 2050 (2047 to 2053) | 2050 (2047 to 2053) |
| India | IND (SEARO) | 2045 (2041 to 2050) | 2045 (2041 to 2050) | 2035 (2033 to 2040) | 2045 (2041 to 2050) | 2045 (2041 to 2050) | 2035 (2034 to 2040) | 2040 (2038 to 2042) |
| Iran | IRN (EMRO) | 2005 (2005 to 2005) | 2005 (2005 to 2005) | 2005 (2005 to 2005) | 2005 (2005 to 2005) | 2005 (2005 to 2005) | 2005 (2005 to 2005) | 2005 (2005 to 2005) |

| Country | ISO country code (WHO region) | Status quo HepB3 & HepB-BD | HepB-BD scale-up (≥25%) | HepB-BD scale-up (≥90%) | HepB-BD disruptions (5%) | HepB-BD disruptions (20%) | delayed HepB-BD scale-up (2023 to 2030) | delayed HepB-BD scale-up (2025 to 2040) |
|---|---|---|---|---|---|---|---|---|
| Iraq | IRQ (EMRO) | 2053 (2044 to 2066) | 2053 (2044 to 2066) | 2033 (2032 to 2034) | 2053 (2044 to 2066) | 2053 (2044 to 2066) | 2034 (2033 to 2035) | 2039 (2037 to 2042) |
| Jamaica | JAM (PAHO) | 2073 (2046 to after 2100) | 2063 (2043 to 2090) | 2034 (2033 to 2035) | 2073 (2046 to after 2100) | 2073 (2046 to after 2100) | 2035 (2034 to 2035) | 2042 (2039 to 2044) |
| Jordan | JOR (EMRO) | 1997 (1988 to 2000) | 1997 (1988 to 2000) | 1997 (1988 to 2000) | 1997 (1988 to 2000) | 1997 (1988 to 2000) | 1997 (1988 to 2000) | 1997 (1988 to 2000) |
| Kenya | KEN (AFRO) | 2022 (2013 to 2023) | 2022 (2013 to 2023) | 2022 (2013 to 2023) | 2022 (2013 to 2023) | 2022 (2013 to 2023) | 2022 (2013 to 2023) | 2022 (2013 to 2023) |
| Kyrgyzstan | KGZ (EURO) | 2019 (2007 to 2029) | 2019 (2007 to 2029) | 2019 (2007 to 2029) | 2019 (2007 to 2029) | 2019 (2007 to 2029) | 2019 (2007 to 2029) | 2019 (2007 to 2029) |
| Cambodia | KHM (WPRO) | 2040 (2037 to 2042) | 2040 (2037 to 2042) | 2039 (2036 to 2041) | 2040 (2037 to 2042) | 2040 (2037 to 2042) | 2039 (2036 to 2041) | 2039 (2036 to 2042) |
| Kiribati | KIR (WPRO) | 2040 (2032 to 2051) | 2040 (2032 to 2051) | 2040 (2032 to 2051) | 2040 (2032 to 2051) | 2040 (2032 to 2051) | 2040 (2032 to 2051) | 2040 (2032 to 2051) |
| Laos | LAO (WPRO) | 2057 (2048 to 2071) | 2057 (2048 to 2071) | 2039 (2034 to 2046) | 2057 (2048 to 2071) | 2057 (2048 to 2071) | 2039 (2034 to 2047) | 2043 (2041 to 2047) |
| Liberia | LBR (AFRO) | after 2100 (2095 to after 2100) | after 2100 (2085 to after 2100) | 2066 (2058 to 2075) | after 2100 (2095 to after 2100) | after 2100 (2095 to after 2100) | 2067 (2059 to 2075) | 2071 (2063 to 2080) |
| Sri Lanka | LKA (SEARO) | 2057 (2043 to 2078) | 2049 (2041 to 2064) | 2034 (2033 to 2036) | 2057 (2043 to 2078) | 2057 (2043 to 2078) | 2034 (2034 to 2036) | 2040 (2038 to 2042) |
| Lesotho | LSO (AFRO) | 2088 (2056 to after 2100) | 2075 (2052 to after 2100) | 2046 (2036 to 2054) | 2088 (2056 to after 2100) | 2088 (2056 to after 2100) | 2046 (2036 to 2055) | 2047 (2042 to 2059) |
| Morocco | MAR (EMRO) | 2005 (2005 to 2006) | 2005 (2005 to 2006) | 2005 (2005 to 2006) | 2005 (2005 to 2006) | 2005 (2005 to 2006) | 2005 (2005 to 2006) | 2005 (2005 to 2006) |
| Moldova | MDA (EURO) | 2043 (2040 to 2051) | 2043 (2040 to 2051) | 2043 (2040 to 2051) | 2043 (2040 to 2052) | 2043 (2040 to 2053) | 2043 (2040 to 2051) | 2043 (2040 to 2051) |
| Madagascar | MDG (AFRO) | after 2100 (after 2100 to after 2100) | after 2100 (2097 to after 2100) | 2053 (2050 to 2055) | after 2100 (after 2100 to after 2100) | after 2100 (after 2100 to after 2100) | 2054 (2051 to 2056) | 2057 (2053 to 2060) |
| Marshall Islands | MHL (WPRO) | 2043 (2039 to 2056) | 2043 (2039 to 2056) | 2043 (2039 to 2056) | 2043 (2039 to 2056) | 2043 (2039 to 2057) | 2043 (2039 to 2056) | 2043 (2039 to 2056) |
| North Macedonia | MKD (EURO) | 2040 (2017 to 2043) | 2040 (2017 to 2043) | 2040 (2017 to 2043) | 2040 (2017 to 2043) | 2040 (2017 to 2043) | 2040 (2017 to 2043) | 2040 (2017 to 2043) |
| Mali | MLI (AFRO) | 2047 (2042 to 2055) | 2044 (2040 to 2051) | 2034 (2033 to 2034) | 2047 (2042 to 2055) | 2047 (2042 to 2055) | 2034 (2033 to 2035) | 2039 (2038 to 2041) |
| Myanmar | MMR (SEARO) | after 2100 (2076 to after 2100) | after 2100 (2074 to after 2100) | 2061 (2052 to 2067) | after 2100 (2076 to after 2100) | after 2100 (2076 to after 2100) | 2062 (2053 to 2068) | 2066 (2053 to 2073) |

| Country | ISO country code (WHO region) | Status quo HepB3 & HepB-BD | HepB-BD scale-up (≥25%) | HepB-BD scale-up (≥90%) | HepB-BD disruptions (5%) | HepB-BD disruptions (20%) | delayed HepB-BD scale-up (2023 to 2030) | delayed HepB-BD scale-up (2025 to 2040) |
|---|---|---|---|---|---|---|---|---|
| Mongolia | MNG (WPRO) | 2024 (2018 to 2027) | 2024 (2018 to 2027) | 2024 (2018 to 2027) | 2024 (2018 to 2027) | 2024 (2018 to 2027) | 2024 (2018 to 2027) | 2024 (2018 to 2027) |
| Mozambique | MOZ (AFRO) | after 2100 (after 2100 to after 2100) | after 2100 (2087 to after 2100) | 2054 (2048 to 2059) | after 2100 (after 2100 to after 2100) | after 2100 (after 2100 to after 2100) | 2055 (2049 to 2060) | 2059 (2050 to 2065) |
| Mauritania | MRT (AFRO) | after 2100 (2080 to after 2100) | 2100 (2071 to after 2100) | 2055 (2045 to 2062) | after 2100 (2080 to after 2100) | after 2100 (2080 to after 2100) | 2056 (2045 to 2063) | 2059 (2046 to 2068) |
| Malawi | MWI (AFRO) | 2036 (2034 to 2040) | 2034 (2033 to 2036) | 2031 (2030 to 2031) | 2036 (2034 to 2040) | 2036 (2034 to 2040) | 2031 (2031 to 2032) | 2034 (2033 to 2035) |
| Namibia | NAM (AFRO) | 2039 (2034 to 2042) | 2039 (2034 to 2042) | 2035 (2030 to 2040) | 2039 (2034 to 2042) | 2039 (2034 to 2042) | 2035 (2031 to 2040) | 2037 (2033 to 2041) |
| Niger | NER (AFRO) | after 2100 (2095 to after 2100) | after 2100 (2085 to after 2100) | 2065 (2057 to 2073) | after 2100 (2095 to after 2100) | after 2100 (2095 to after 2100) | 2066 (2058 to 2074) | 2070 (2061 to 2079) |
| Nigeria | NGA (AFRO) | after 2100 (2092 to after 2100) | 2090 (2082 to after 2100) | 2058 (2055 to 2062) | after 2100 (2092 to after 2100) | after 2100 (2092 to after 2100) | 2059 (2056 to 2063) | 2061 (2057 to 2067) |
| Nicaragua | NIC (PAHO) | 2003 (2003 to 2004) | 2003 (2003 to 2004) | 2003 (2003 to 2004) | 2003 (2003 to 2004) | 2003 (2003 to 2004) | 2003 (2003 to 2004) | 2003 (2003 to 2004) |
| Nepal | NPL (SEARO) | 2048 (2037 to 2081) | 2041 (2034 to 2058) | 2032 (2031 to 2032) | 2048 (2037 to 2081) | 2048 (2037 to 2081) | 2032 (2032 to 2033) | 2037 (2034 to 2040) |
| Pakistan | PAK (EMRO) | 2078 (2045 to after 2100) | 2062 (2041 to 2084) | 2034 (2032 to 2034) | 2078 (2045 to after 2100) | 2078 (2045 to after 2100) | 2034 (2033 to 2035) | 2042 (2038 to 2043) |
| Peru | PER (PAHO) | 2008 (2008 to 2009) | 2008 (2008 to 2009) | 2008 (2008 to 2009) | 2008 (2008 to 2009) | 2008 (2008 to 2009) | 2008 (2008 to 2009) | 2008 (2008 to 2009) |
| Philippines | PHL (WPRO) | 2056 (2052 to 2061) | 2056 (2052 to 2061) | 2044 (2041 to 2047) | 2056 (2052 to 2061) | 2056 (2052 to 2061) | 2044 (2041 to 2047) | 2045 (2043 to 2047) |
| Papua New Guinea | PNG (WPRO) | 2093 (2068 to after 2100) | 2093 (2068 to after 2100) | 2056 (2048 to 2064) | 2093 (2068 to after 2100) | 2093 (2068 to after 2100) | 2057 (2049 to 2065) | 2059 (2049 to 2070) |
| Korea, North | PRK (SEARO) | 2042 (2039 to 2043) | 2042 (2039 to 2043) | 2042 (2039 to 2043) | 2042 (2039 to 2043) | 2042 (2039 to 2043) | 2042 (2039 to 2043) | 2042 (2039 to 2043) |
| Paraguay | PRY (PAHO) | 2084 (2054 to after 2100) | 2068 (2049 to 2094) | 2035 (2034 to 2042) | 2084 (2054 to after 2100) | 2084 (2054 to after 2100) | 2035 (2034 to 2042) | 2043 (2040 to 2045) |
| Rwanda | RWA (AFRO) | 2055 (2045 to 2071) | 2046 (2039 to 2055) | 2031 (2031 to 2032) | 2055 (2045 to 2071) | 2055 (2045 to 2071) | 2032 (2032 to 2033) | 2037 (2036 to 2039) |
| Sudan | SDN (EMRO) | 2098 (2068 to after 2100) | 2077 (2059 to after 2100) | 2035 (2034 to 2044) | 2098 (2068 to after 2100) | 2098 (2068 to after 2100) | 2035 (2035 to 2044) | 2044 (2042 to 2045) |
| Senegal | SEN (AFRO) | 2050 (2042 to 2055) | 2050 (2042 to 2055) | 2044 (2037 to 2049) | 2050 (2042 to 2055) | 2050 (2042 to 2055) | 2044 (2037 to 2049) | 2044 (2039 to 2049) |

| Country | ISO country code (WHO region) | Status quo HepB3 & HepB-BD | HepB-BD scale-up (≥25%) | HepB-BD scale-up (≥90%) | HepB-BD disruptions (5%) | HepB-BD disruptions (20%) | delayed HepB-BD scale-up (2023 to 2030) | delayed HepB-BD scale-up (2025 to 2040) |
|---|---|---|---|---|---|---|---|---|
| Solomon Islands | SLB (WPRO) | 2052 (2046 to 2057) | 2052 (2046 to 2057) | 2044 (2038 to 2049) | 2052 (2046 to 2058) | 2052 (2046 to 2058) | 2044 (2038 to 2049) | 2044 (2041 to 2050) |
| Sierra Leone | SLE (AFRO) | 2091 (2080 to after 2100) | 2082 (2074 to 2093) | 2058 (2054 to 2063) | 2091 (2080 to after 2100) | 2091 (2080 to after 2100) | 2059 (2055 to 2064) | 2062 (2057 to 2067) |
| El Salvador | SLV (PAHO) | 2020 (2020 to 2020) | 2020 (2020 to 2020) | 2020 (2020 to 2020) | 2020 (2020 to 2020) | 2020 (2020 to 2020) | 2020 (2020 to 2020) | 2020 (2020 to 2020) |
| Somalia | SOM (EMRO) | after 2100 (2095 to after 2100) | after 2100 (2085 to after 2100) | 2068 (2059 to 2080) | after 2100 (2095 to after 2100) | after 2100 (2095 to after 2100) | 2068 (2059 to 2080) | 2073 (2063 to 2083) |
| Serbia | SRB (EURO) | 2018 (2010 to 2036) | 2018 (2010 to 2036) | 2018 (2010 to 2036) | 2018 (2010 to 2036) | 2018 (2010 to 2036) | 2018 (2010 to 2036) | 2018 (2010 to 2036) |
| South Sudan | SSD (AFRO) | after 2100 (2088 to after 2100) | 2096 (2082 to after 2100) | 2063 (2059 to 2071) | after 2100 (2088 to after 2100) | after 2100 (2088 to after 2100) | 2064 (2059 to 2071) | 2069 (2062 to 2075) |
| Sao Tome and Principe | STP (AFRO) | 2040 (2030 to 2046) | 2040 (2030 to 2046) | 2040 (2030 to 2046) | 2040 (2030 to 2046) | 2040 (2030 to 2046) | 2040 (2030 to 2046) | 2040 (2030 to 2046) |
| Eswatini | SWZ (AFRO) | 2050 (2039 to 2065) | 2046 (2036 to 2057) | 2033 (2032 to 2034) | 2050 (2039 to 2065) | 2050 (2039 to 2065) | 2034 (2032 to 2035) | 2039 (2035 to 2042) |
| Syria | SYR (EMRO) | 2056 (2051 to 2061) | 2053 (2048 to 2058) | 2034 (2034 to 2037) | 2056 (2051 to 2061) | 2056 (2051 to 2061) | 2035 (2034 to 2037) | 2041 (2039 to 2044) |
| Chad | TCD (AFRO) | after 2100 (2099 to after 2100) | after 2100 (2088 to after 2100) | 2069 (2060 to 2078) | after 2100 (2099 to after 2100) | after 2100 (2099 to after 2100) | 2070 (2060 to 2079) | 2074 (2063 to 2084) |
| Togo | TGO (AFRO) | after 2100 (2084 to after 2100) | after 2100 (2075 to after 2100) | 2059 (2052 to 2066) | after 2100 (2084 to after 2100) | after 2100 (2084 to after 2100) | 2060 (2052 to 2067) | 2064 (2053 to 2072) |
| Thailand | THA (SEARO) | 2022 (2022 to 2022) | 2022 (2022 to 2022) | 2022 (2022 to 2022) | 2022 (2022 to 2022) | 2022 (2022 to 2022) | 2022 (2022 to 2022) | 2022 (2022 to 2022) |
| Tajikistan | TJK (EURO) | 2033 (2029 to 2035) | 2033 (2029 to 2035) | 2033 (2029 to 2035) | 2033 (2029 to 2035) | 2033 (2029 to 2035) | 2033 (2029 to 2035) | 2033 (2029 to 2035) |
| Turkmenistan | TKM (EURO) | 2038 (2034 to 2040) | 2038 (2034 to 2040) | 2038 (2034 to 2040) | 2038 (2034 to 2040) | 2038 (2034 to 2040) | 2038 (2034 to 2040) | 2038 (2034 to 2040) |
| Timor-Leste | TLS (SEARO) | 2050 (2044 to 2056) | 2050 (2044 to 2056) | 2041 (2034 to 2048) | 2050 (2044 to 2056) | 2050 (2044 to 2057) | 2041 (2034 to 2048) | 2043 (2040 to 2048) |
| Tonga | TON (WPRO) | 2051 (2043 to 2063) | 2051 (2043 to 2063) | 2051 (2043 to 2063) | 2052 (2043 to 2063) | 2053 (2043 to 2063) | 2051 (2043 to 2063) | 2051 (2043 to 2063) |
| Tunisia | TUN (EMRO) | 2032 (2013 to 2033) | 2032 (2013 to 2033) | 2029 (2013 to 2030) | 2032 (2013 to 2033) | 2032 (2013 to 2033) | 2030 (2013 to 2031) | 2031 (2013 to 2032) |
| Tuvalu | TUV (WPRO) | 2040 (2035 to 2046) | 2040 (2035 to 2046) | 2040 (2035 to 2046) | 2040 (2035 to 2047) | 2040 (2035 to 2047) | 2040 (2035 to 2046) | 2040 (2035 to 2046) |

| Country | ISO country code (WHO region) | Status quo HepB3 & HepB-BD | HepB-BD scale-up (≥25%) | HepB-BD scale-up (≥90%) | HepB-BD disruptions (5%) | HepB-BD disruptions (20%) | delayed HepB-BD scale-up (2023 to 2030) | delayed HepB-BD scale-up (2025 to 2040) |
|---|---|---|---|---|---|---|---|---|
| Tanzania | TZA (AFRO) | 2080 (2055 to after 2100) | 2065 (2049 to 2089) | 2034 (2033 to 2038) | 2080 (2055 to after 2100) | 2080 (2055 to after 2100) | 2035 (2034 to 2038) | 2043 (2040 to 2044) |
| Uganda | UGA (AFRO) | after 2100 (2080 to after 2100) | 2094 (2069 to after 2100) | 2048 (2038 to 2053) | after 2100 (2080 to after 2100) | after 2100 (2080 to after 2100) | 2048 (2038 to 2054) | 2049 (2044 to 2058) |
| Ukraine | UKR (EURO) | 2042 (2009 to 2058) | 2042 (2009 to 2058) | 2034 (2009 to 2036) | 2042 (2009 to 2058) | 2042 (2009 to 2058) | 2034 (2009 to 2036) | 2037 (2009 to 2041) |
| Uzbekistan | UZB (EURO) | 2037 (2034 to 2039) | 2037 (2034 to 2039) | 2037 (2034 to 2039) | 2037 (2034 to 2039) | 2037 (2034 to 2039) | 2037 (2034 to 2039) | 2037 (2034 to 2039) |
| Venezuela | VEN (PAHO) | 2039 (2032 to 2047) | 2039 (2032 to 2047) | 2031 (2029 to 2033) | 2039 (2032 to 2047) | 2039 (2032 to 2048) | 2032 (2030 to 2033) | 2035 (2032 to 2037) |
| Viet Nam | VNM (WPRO) | 2054 (2046 to 2061) | 2054 (2046 to 2061) | 2048 (2043 to 2053) | 2054 (2046 to 2061) | 2054 (2046 to 2062) | 2048 (2043 to 2054) | 2048 (2043 to 2054) |
| Vanuatu | VUT (WPRO) | 2072 (2060 to 2082) | 2072 (2060 to 2082) | 2063 (2056 to 2073) | 2072 (2060 to 2082) | 2072 (2060 to 2082) | 2063 (2056 to 2073) | 2064 (2057 to 2074) |
| Samoa | WSM (WPRO) | 2068 (2059 to 2085) | 2068 (2059 to 2085) | 2053 (2042 to 2061) | 2068 (2059 to 2085) | 2068 (2059 to 2085) | 2054 (2042 to 2061) | 2054 (2044 to 2063) |
| Yemen | YEM (EMRO) | 2095 (2062 to after 2100) | 2076 (2055 to after 2100) | 2035 (2034 to 2045) | 2095 (2062 to after 2100) | 2095 (2062 to after 2100) | 2035 (2034 to 2046) | 2044 (2041 to 2046) |
| South Africa | ZAF (AFRO) | after 2100 (2064 to after 2100) | 2096 (2059 to after 2100) | 2051 (2037 to 2057) | after 2100 (2064 to after 2100) | after 2100 (2064 to after 2100) | 2052 (2037 to 2059) | 2055 (2044 to 2063) |
| Zambia | ZMB (AFRO) | 2026 (2024 to 2031) | 2026 (2024 to 2031) | 2026 (2024 to 2031) | 2026 (2024 to 2031) | 2026 (2024 to 2031) | 2026 (2024 to 2031) | 2026 (2024 to 2031) |
| Zimbabwe | ZWE (AFRO) | 2052 (2045 to 2060) | 2048 (2042 to 2055) | 2034 (2033 to 2036) | 2052 (2045 to 2060) | 2052 (2045 to 2060) | 2035 (2034 to 2036) | 2040 (2038 to 2042) |
| Treatment level such that 40% of treatment-eligible individuals are in treatment in 2030 | | | | | | | | |
| Afghanistan | AFG (EMRO) | 2051 (2042 to 2059) | 2051 (2042 to 2059) | 2034 (2033 to 2035) | 2051 (2042 to 2059) | 2051 (2042 to 2059) | 2034 (2033 to 2036) | 2040 (2038 to 2041) |
| Angola | AGO (AFRO) | after 2100 (2080 to after 2100) | after 2100 (2072 to after 2100) | 2057 (2050 to 2060) | after 2100 (2080 to after 2100) | after 2100 (2080 to after 2100) | 2058 (2051 to 2061) | 2062 (2052 to 2065) |
| Albania | ALB (EURO) | 2023 (2009 to 2026) | 2023 (2009 to 2026) | 2023 (2009 to 2026) | 2023 (2009 to 2026) | 2023 (2009 to 2026) | 2023 (2009 to 2026) | 2023 (2009 to 2026) |
| Armenia | ARM (EURO) | 2031 (2018 to 2034) | 2031 (2018 to 2034) | 2031 (2018 to 2034) | 2031 (2018 to 2034) | 2031 (2018 to 2034) | 2031 (2018 to 2034) | 2031 (2018 to 2034) |
| Azerbaijan | AZE (EURO) | 2033 (2029 to 2037) | 2033 (2029 to 2037) | 2033 (2029 to 2037) | 2033 (2029 to 2037) | 2033 (2029 to 2037) | 2033 (2029 to 2037) | 2033 (2029 to 2037) |
| Burundi | BDI (AFRO) | 2062 (2051 to 2069) | 2052 (2045 to 2057) | 2032 (2032 to 2033) | 2062 (2051 to 2069) | 2062 (2051 to 2069) | 2033 (2033 to 2033) | 2039 (2038 to 2040) |

| Country | ISO country code (WHO region) | Status quo HepB3 & HepB-BD | HepB-BD scale-up (≥25%) | HepB-BD scale-up (≥90%) | HepB-BD disruptions (5%) | HepB-BD disruptions (20%) | delayed HepB-BD scale-up (2023 to 2030) | delayed HepB-BD scale-up (2025 to 2040) |
|---|---|---|---|---|---|---|---|---|
| Benin | BEN (AFRO) | 2098 (2082 to after 2100) | 2086 (2074 to after 2100) | 2057 (2053 to 2060) | 2098 (2082 to after 2100) | 2098 (2082 to after 2100) | 2058 (2054 to 2061) | 2061 (2056 to 2065) |
| Burkina Faso | BFA (AFRO) | 2051 (2045 to 2064) | 2047 (2043 to 2057) | 2035 (2034 to 2036) | 2051 (2045 to 2064) | 2051 (2045 to 2064) | 2035 (2034 to 2036) | 2041 (2040 to 2042) |
| Bangladesh | BGD (SEARO) | 2077 (2059 to 2097) | 2062 (2052 to 2075) | 2034 (2034 to 2035) | 2077 (2059 to 2097) | 2077 (2059 to 2097) | 2034 (2034 to 2035) | 2042 (2040 to 2043) |
| Bosnia and Herzegovina | BIH (EURO) | 2044 (2040 to 2056) | 2040 (2037 to 2047) | 2032 (2031 to 2032) | 2044 (2040 to 2056) | 2044 (2040 to 2056) | 2033 (2032 to 2033) | 2037 (2036 to 2038) |
| Belarus | BLR (EURO) | 2017 (2010 to 2028) | 2017 (2010 to 2028) | 2017 (2010 to 2028) | 2017 (2010 to 2028) | 2017 (2010 to 2028) | 2017 (2010 to 2028) | 2017 (2010 to 2028) |
| Belize | BLZ (PAHO) | 2016 (2010 to 2020) | 2016 (2010 to 2020) | 2016 (2010 to 2020) | 2016 (2010 to 2020) | 2016 (2010 to 2020) | 2016 (2010 to 2020) | 2016 (2010 to 2020) |
| Bolivia | BOL (PAHO) | 2035 (2030 to 2044) | 2032 (2029 to 2036) | 2029 (2028 to 2030) | 2035 (2030 to 2044) | 2035 (2030 to 2044) | 2030 (2029 to 2031) | 2033 (2030 to 2035) |
| Bhutan | BTN (SEARO) | 2045 (2035 to 2048) | 2045 (2035 to 2048) | 2043 (2034 to 2046) | 2045 (2035 to 2048) | 2045 (2035 to 2048) | 2043 (2034 to 2046) | 2043 (2034 to 2046) |
| Central African Republic | CAF (AFRO) | after 2100 (after 2100 to after 2100) | after 2100 (2096 to after 2100) | 2068 (2062 to 2072) | after 2100 (after 2100 to after 2100) | after 2100 (after 2100 to after 2100) | 2069 (2063 to 2073) | 2074 (2067 to 2078) |
| China | CHN (WPRO) | 2026 (2021 to 2029) | 2026 (2021 to 2029) | 2026 (2021 to 2029) | 2026 (2021 to 2029) | 2026 (2021 to 2029) | 2026 (2021 to 2029) | 2026 (2021 to 2029) |
| Ivory Coast | CIV (AFRO) | after 2100 (2066 to after 2100) | 2097 (2062 to after 2100) | 2056 (2045 to 2060) | after 2100 (2066 to after 2100) | after 2100 (2066 to after 2100) | 2057 (2045 to 2061) | 2060 (2046 to 2065) |
| Cameroon | CMR (AFRO) | after 2100 (2071 to after 2100) | 2088 (2064 to after 2100) | 2050 (2041 to 2053) | after 2100 (2071 to after 2100) | after 2100 (2071 to after 2100) | 2051 (2041 to 2054) | 2053 (2045 to 2057) |
| Democratic Republic of the Congo | COD (AFRO) | after 2100 (2074 to after 2100) | 2083 (2066 to 2097) | 2049 (2041 to 2053) | after 2100 (2074 to after 2100) | after 2100 (2074 to after 2100) | 2050 (2041 to 2054) | 2051 (2045 to 2057) |
| Congo | COG (AFRO) | after 2100 (2074 to after 2100) | 2095 (2069 to after 2100) | 2058 (2051 to 2063) | after 2100 (2074 to after 2100) | after 2100 (2074 to after 2100) | 2059 (2051 to 2064) | 2062 (2053 to 2068) |
| Colombia | COL (PAHO) | 2016 (2009 to 2018) | 2016 (2009 to 2018) | 2016 (2009 to 2018) | 2016 (2009 to 2018) | 2016 (2009 to 2018) | 2016 (2009 to 2018) | 2016 (2009 to 2018) |
| Comoros | COM (AFRO) | 2067 (2059 to 2083) | 2062 (2055 to 2073) | 2042 (2038 to 2048) | 2067 (2059 to 2083) | 2067 (2059 to 2083) | 2042 (2038 to 2048) | 2044 (2043 to 2048) |
| Cape Verde | CPV (AFRO) | 2043 (2039 to 2048) | 2043 (2039 to 2048) | 2043 (2039 to 2048) | 2043 (2039 to 2048) | 2043 (2039 to 2048) | 2043 (2039 to 2048) | 2043 (2039 to 2048) |

| Country | ISO country code (WHO region) | Status quo HepB3 & HepB-BD | HepB-BD scale-up (≥25%) | HepB-BD scale-up (≥90%) | HepB-BD disruptions (5%) | HepB-BD disruptions (20%) | delayed HepB-BD scale-up (2023 to 2030) | delayed HepB-BD scale-up (2025 to 2040) |
|---|---|---|---|---|---|---|---|---|
| Cuba | CUB (PAHO) | 2005 (2005 to 2005) | 2005 (2005 to 2005) | 2005 (2005 to 2005) | 2005 (2005 to 2005) | 2005 (2005 to 2005) | 2005 (2005 to 2005) | 2005 (2005 to 2005) |
| Djibouti | DJI (EMRO) | 2044 (2038 to 2046) | 2044 (2038 to 2046) | 2044 (2038 to 2046) | 2044 (2038 to 2046) | 2044 (2038 to 2047) | 2044 (2038 to 2046) | 2044 (2038 to 2046) |
| Algeria | DZA (AFRO) | 2009 (2009 to 2011) | 2009 (2009 to 2011) | 2009 (2009 to 2011) | 2009 (2009 to 2011) | 2009 (2009 to 2011) | 2009 (2009 to 2011) | 2009 (2009 to 2011) |
| Ecuador | ECU (PAHO) | 2036 (2031 to 2040) | 2036 (2031 to 2040) | 2030 (2028 to 2032) | 2036 (2031 to 2040) | 2036 (2031 to 2040) | 2031 (2029 to 2033) | 2034 (2031 to 2036) |
| Egypt | EGY (EMRO) | 2022 (2022 to 2022) | 2022 (2022 to 2022) | 2022 (2022 to 2022) | 2022 (2022 to 2022) | 2022 (2022 to 2022) | 2022 (2022 to 2022) | 2022 (2022 to 2022) |
| Eritrea | ERI (AFRO) | 2057 (2040 to 2082) | 2051 (2039 to 2066) | 2034 (2033 to 2035) | 2057 (2040 to 2082) | 2057 (2040 to 2082) | 2034 (2033 to 2035) | 2040 (2037 to 2043) |
| Ethiopia | ETH (AFRO) | 2076 (2072 to 2084) | 2069 (2065 to 2075) | 2046 (2045 to 2049) | 2076 (2072 to 2084) | 2076 (2072 to 2084) | 2047 (2045 to 2049) | 2047 (2046 to 2050) |
| Fiji | FJI (WPRO) | 2031 (2025 to 2035) | 2031 (2025 to 2035) | 2031 (2025 to 2035) | 2031 (2026 to 2035) | 2031 (2026 to 2035) | 2031 (2025 to 2035) | 2031 (2025 to 2035) |
| Micronesia | FSM (WPRO) | 2058 (2042 to 2065) | 2058 (2042 to 2065) | 2041 (2036 to 2045) | 2058 (2042 to 2065) | 2059 (2042 to 2065) | 2041 (2036 to 2045) | 2042 (2039 to 2045) |
| Georgia | GEO (EURO) | 2036 (2032 to 2040) | 2036 (2032 to 2040) | 2036 (2032 to 2040) | 2036 (2032 to 2040) | 2036 (2032 to 2040) | 2036 (2032 to 2040) | 2036 (2032 to 2040) |
| Ghana | GHA (AFRO) | after 2100 (2088 to after 2100) | 2098 (2075 to after 2100) | 2053 (2044 to 2056) | after 2100 (2088 to after 2100) | after 2100 (2088 to after 2100) | 2054 (2044 to 2057) | 2057 (2045 to 2061) |
| Guinea | GIN (AFRO) | after 2100 (after 2100 to after 2100) | after 2100 (after 2100 to after 2100) | 2070 (2065 to 2075) | after 2100 (after 2100 to after 2100) | after 2100 (after 2100 to after 2100) | 2071 (2066 to 2076) | 2076 (2070 to 2080) |
| Gambia | GMB (AFRO) | 2064 (2042 to after 2100) | 2058 (2039 to 2085) | 2034 (2032 to 2037) | 2064 (2042 to after 2100) | 2064 (2042 to after 2100) | 2034 (2033 to 2037) | 2042 (2037 to 2045) |
| Guinea-Bissau | GNB (AFRO) | after 2100 (2072 to after 2100) | 2090 (2066 to after 2100) | 2053 (2046 to 2058) | after 2100 (2072 to after 2100) | after 2100 (2072 to after 2100) | 2054 (2046 to 2059) | 2057 (2047 to 2063) |
| Guatemala | GTM (PAHO) | 2012 (2011 to 2012) | 2012 (2011 to 2012) | 2012 (2011 to 2012) | 2012 (2011 to 2012) | 2012 (2011 to 2012) | 2012 (2011 to 2012) | 2012 (2011 to 2012) |
| Guyana | GUY (PAHO) | 2089 (2059 to after 2100) | 2073 (2053 to 2083) | 2036 (2034 to 2045) | 2089 (2059 to after 2100) | 2089 (2059 to after 2100) | 2036 (2034 to 2045) | 2044 (2042 to 2045) |
| Honduras | HND (PAHO) | 2041 (2035 to 2044) | 2041 (2035 to 2044) | 2034 (2032 to 2036) | 2041 (2035 to 2044) | 2041 (2035 to 2044) | 2034 (2032 to 2036) | 2037 (2034 to 2039) |
| Haiti | HTI (PAHO) | after 2100 (2077 to after 2100) | 2084 (2065 to 2094) | 2043 (2035 to 2049) | after 2100 (2077 to after 2100) | after 2100 (2077 to after 2100) | 2043 (2035 to 2049) | 2045 (2044 to 2051) |

| Country | ISO country code (WHO region) | Status quo HepB3 & HepB-BD | HepB-BD scale-up (≥25%) | HepB-BD scale-up (≥90%) | HepB-BD disruptions (5%) | HepB-BD disruptions (20%) | delayed HepB-BD scale-up (2023 to 2030) | delayed HepB-BD scale-up (2025 to 2040) |
|---|---|---|---|---|---|---|---|---|
| Indonesia | IDN (SEARO) | 2052 (2050 to 2054) | 2052 (2050 to 2054) | 2049 (2046 to 2051) | 2052 (2050 to 2054) | 2052 (2050 to 2054) | 2049 (2046 to 2051) | 2049 (2046 to 2051) |
| India | IND (SEARO) | 2045 (2041 to 2049) | 2045 (2041 to 2049) | 2035 (2033 to 2038) | 2045 (2041 to 2049) | 2045 (2041 to 2049) | 2035 (2033 to 2038) | 2040 (2037 to 2042) |
| Iran | IRN (EMRO) | 2005 (2005 to 2005) | 2005 (2005 to 2005) | 2005 (2005 to 2005) | 2005 (2005 to 2005) | 2005 (2005 to 2005) | 2005 (2005 to 2005) | 2005 (2005 to 2005) |
| Iraq | IRQ (EMRO) | 2051 (2043 to 2057) | 2051 (2043 to 2057) | 2033 (2032 to 2034) | 2051 (2043 to 2057) | 2051 (2043 to 2057) | 2034 (2033 to 2034) | 2038 (2037 to 2040) |
| Jamaica | JAM (PAHO) | 2067 (2044 to 2087) | 2058 (2042 to 2070) | 2034 (2033 to 2035) | 2067 (2044 to 2087) | 2067 (2044 to 2087) | 2034 (2034 to 2035) | 2042 (2038 to 2043) |
| Jordan | JOR (EMRO) | 1997 (1988 to 2000) | 1997 (1988 to 2000) | 1997 (1988 to 2000) | 1997 (1988 to 2000) | 1997 (1988 to 2000) | 1997 (1988 to 2000) | 1997 (1988 to 2000) |
| Kenya | KEN (AFRO) | 2022 (2013 to 2023) | 2022 (2013 to 2023) | 2022 (2013 to 2023) | 2022 (2013 to 2023) | 2022 (2013 to 2023) | 2022 (2013 to 2023) | 2022 (2013 to 2023) |
| Kyrgyzstan | KGZ (EURO) | 2019 (2007 to 2028) | 2019 (2007 to 2028) | 2019 (2007 to 2028) | 2019 (2007 to 2028) | 2019 (2007 to 2028) | 2019 (2007 to 2028) | 2019 (2007 to 2028) |
| Cambodia | KHM (WPRO) | 2039 (2036 to 2041) | 2039 (2036 to 2041) | 2038 (2035 to 2041) | 2039 (2036 to 2041) | 2039 (2036 to 2041) | 2038 (2035 to 2041) | 2039 (2036 to 2041) |
| Kiribati | KIR (WPRO) | 2038 (2031 to 2046) | 2038 (2031 to 2046) | 2038 (2031 to 2046) | 2038 (2031 to 2046) | 2038 (2031 to 2046) | 2038 (2031 to 2046) | 2038 (2031 to 2046) |
| Laos | LAO (WPRO) | 2056 (2050 to 2060) | 2056 (2050 to 2060) | 2038 (2035 to 2043) | 2056 (2050 to 2060) | 2056 (2050 to 2060) | 2038 (2035 to 2043) | 2043 (2041 to 2044) |
| Liberia | LBR (AFRO) | after 2100 (after 2100 to after 2100) | after 2100 (2091 to after 2100) | 2066 (2061 to 2070) | after 2100 (after 2100 to after 2100) | after 2100 (after 2100 to after 2100) | 2067 (2062 to 2070) | 2071 (2065 to 2076) |
| Sri Lanka | LKA (SEARO) | 2049 (2042 to 2065) | 2044 (2040 to 2057) | 2034 (2033 to 2035) | 2049 (2042 to 2065) | 2049 (2042 to 2065) | 2034 (2034 to 2035) | 2039 (2038 to 2041) |
| Lesotho | LSO (AFRO) | 2083 (2056 to after 2100) | 2071 (2051 to 2096) | 2043 (2036 to 2050) | 2083 (2056 to after 2100) | 2083 (2056 to after 2100) | 2044 (2036 to 2051) | 2045 (2042 to 2054) |
| Morocco | MAR (EMRO) | 2005 (2005 to 2006) | 2005 (2005 to 2006) | 2005 (2005 to 2006) | 2005 (2005 to 2006) | 2005 (2005 to 2006) | 2005 (2005 to 2006) | 2005 (2005 to 2006) |
| Moldova | MDA (EURO) | 2042 (2039 to 2046) | 2042 (2039 to 2046) | 2042 (2039 to 2046) | 2042 (2039 to 2046) | 2042 (2039 to 2047) | 2042 (2039 to 2046) | 2042 (2039 to 2046) |
| Madagascar | MDG (AFRO) | after 2100 (after 2100 to after 2100) | after 2100 (2099 to after 2100) | 2052 (2051 to 2053) | after 2100 (after 2100 to after 2100) | after 2100 (after 2100 to after 2100) | 2053 (2052 to 2054) | 2056 (2055 to 2058) |

| Country | ISO country code (WHO region) | Status quo HepB3 & HepB-BD | HepB-BD scale-up (≥25%) | HepB-BD scale-up (≥90%) | HepB-BD disruptions (5%) | HepB-BD disruptions (20%) | delayed HepB-BD scale-up (2023 to 2030) | delayed HepB-BD scale-up (2025 to 2040) |
|---|---|---|---|---|---|---|---|---|
| Marshall Islands | MHL (WPRO) | 2043 (2038 to 2052) | 2043 (2038 to 2052) | 2043 (2038 to 2052) | 2043 (2038 to 2052) | 2043 (2038 to 2053) | 2043 (2038 to 2052) | 2043 (2038 to 2052) |
| North Macedonia | MKD (EURO) | 2038 (2017 to 2042) | 2038 (2017 to 2042) | 2038 (2017 to 2042) | 2038 (2017 to 2042) | 2038 (2017 to 2042) | 2038 (2017 to 2042) | 2038 (2017 to 2042) |
| Mali | MLI (AFRO) | 2051 (2045 to 2058) | 2047 (2042 to 2053) | 2034 (2034 to 2035) | 2051 (2045 to 2058) | 2051 (2045 to 2058) | 2035 (2034 to 2035) | 2040 (2039 to 2042) |
| Myanmar | MMR (SEARO) | 2099 (2073 to after 2100) | 2095 (2071 to after 2100) | 2060 (2051 to 2065) | 2099 (2073 to after 2100) | 2099 (2073 to after 2100) | 2061 (2052 to 2066) | 2064 (2052 to 2071) |
| Mongolia | MNG (WPRO) | 2024 (2018 to 2026) | 2024 (2018 to 2026) | 2024 (2018 to 2026) | 2024 (2018 to 2026) | 2024 (2018 to 2027) | 2024 (2018 to 2026) | 2024 (2018 to 2026) |
| Mozambique | MOZ (AFRO) | after 2100 (2100 to after 2100) | 2096 (2083 to after 2100) | 2051 (2046 to 2054) | after 2100 (2100 to after 2100) | after 2100 (2100 to after 2100) | 2052 (2047 to 2055) | 2055 (2048 to 2059) |
| Mauritania | MRT (AFRO) | after 2100 (2080 to after 2100) | 2092 (2071 to after 2100) | 2053 (2044 to 2056) | after 2100 (2080 to after 2100) | after 2100 (2080 to after 2100) | 2053 (2044 to 2057) | 2056 (2045 to 2061) |
| Malawi | MWI (AFRO) | 2035 (2034 to 2038) | 2033 (2032 to 2035) | 2030 (2030 to 2031) | 2035 (2034 to 2038) | 2035 (2034 to 2038) | 2031 (2031 to 2032) | 2033 (2033 to 2035) |
| Namibia | NAM (AFRO) | 2038 (2034 to 2040) | 2038 (2034 to 2040) | 2034 (2030 to 2038) | 2038 (2034 to 2040) | 2038 (2034 to 2040) | 2034 (2031 to 2038) | 2036 (2033 to 2039) |
| Niger | NER (AFRO) | after 2100 (2092 to after 2100) | after 2100 (2082 to after 2100) | 2063 (2057 to 2067) | after 2100 (2092 to after 2100) | after 2100 (2092 to after 2100) | 2064 (2058 to 2068) | 2069 (2061 to 2073) |
| Nigeria | NGA (AFRO) | after 2100 (after 2100 to after 2100) | after 2100 (after 2100 to after 2100) | 2063 (2061 to 2067) | after 2100 (after 2100 to after 2100) | after 2100 (after 2100 to after 2100) | 2064 (2062 to 2068) | 2069 (2066 to 2072) |
| Nicaragua | NIC (PAHO) | 2003 (2003 to 2004) | 2003 (2003 to 2004) | 2003 (2003 to 2004) | 2003 (2003 to 2004) | 2003 (2003 to 2004) | 2003 (2003 to 2004) | 2003 (2003 to 2004) |
| Nepal | NPL (SEARO) | 2045 (2035 to 2062) | 2039 (2034 to 2051) | 2031 (2031 to 2032) | 2045 (2035 to 2062) | 2045 (2035 to 2062) | 2032 (2031 to 2033) | 2036 (2034 to 2038) |
| Pakistan | PAK (EMRO) | 2076 (2044 to 2092) | 2062 (2041 to 2070) | 2034 (2033 to 2034) | 2076 (2044 to 2092) | 2076 (2044 to 2092) | 2034 (2033 to 2035) | 2042 (2038 to 2043) |
| Peru | PER (PAHO) | 2008 (2008 to 2009) | 2008 (2008 to 2009) | 2008 (2008 to 2009) | 2008 (2008 to 2009) | 2008 (2008 to 2009) | 2008 (2008 to 2009) | 2008 (2008 to 2009) |
| Philippines | PHL (WPRO) | 2062 (2057 to 2066) | 2062 (2057 to 2066) | 2046 (2043 to 2049) | 2062 (2057 to 2066) | 2062 (2057 to 2067) | 2046 (2043 to 2050) | 2047 (2045 to 2050) |
| Papua New Guinea | PNG (WPRO) | after 2100 (2099 to after 2100) | after 2100 (2099 to after 2100) | 2062 (2057 to 2067) | after 2100 (2099 to after 2100) | after 2100 (2099 to after 2100) | 2063 (2058 to 2067) | 2066 (2061 to 2071) |
| Korea, North | PRK (SEARO) | 2041 (2039 to 2042) | 2041 (2039 to 2042) | 2041 (2039 to 2042) | 2041 (2039 to 2042) | 2041 (2039 to 2042) | 2041 (2039 to 2042) | 2041 (2039 to 2042) |

| Country | ISO country code (WHO region) | Status quo HepB3 & HepB-BD | HepB-BD scale-up (≥25%) | HepB-BD scale-up (≥90%) | HepB-BD disruptions (5%) | HepB-BD disruptions (20%) | delayed HepB-BD scale-up (2023 to 2030) | delayed HepB-BD scale-up (2025 to 2040) |
|---|---|---|---|---|---|---|---|---|
| Paraguay | PRY (PAHO) | 2077 (2052 to 2095) | 2064 (2047 to 2074) | 2035 (2034 to 2035) | 2077 (2052 to 2095) | 2077 (2052 to 2095) | 2035 (2034 to 2036) | 2043 (2040 to 2044) |
| Rwanda | RWA (AFRO) | 2049 (2042 to 2053) | 2041 (2037 to 2044) | 2031 (2031 to 2031) | 2049 (2042 to 2053) | 2049 (2042 to 2053) | 2032 (2032 to 2032) | 2037 (2035 to 2037) |
| Sudan | SDN (EMRO) | 2084 (2063 to 2096) | 2069 (2055 to 2077) | 2035 (2034 to 2037) | 2084 (2063 to 2096) | 2084 (2063 to 2096) | 2035 (2035 to 2037) | 2043 (2042 to 2044) |
| Senegal | SEN (AFRO) | 2049 (2042 to 2053) | 2049 (2042 to 2053) | 2043 (2037 to 2047) | 2049 (2042 to 2053) | 2049 (2042 to 2053) | 2043 (2037 to 2047) | 2044 (2039 to 2047) |
| Solomon Islands | SLB (WPRO) | 2050 (2045 to 2054) | 2050 (2045 to 2054) | 2042 (2037 to 2047) | 2050 (2045 to 2054) | 2050 (2045 to 2054) | 2042 (2037 to 2047) | 2043 (2041 to 2047) |
| Sierra Leone | SLE (AFRO) | 2086 (2078 to 2096) | 2078 (2072 to 2086) | 2057 (2053 to 2060) | 2086 (2078 to 2096) | 2086 (2078 to 2096) | 2058 (2054 to 2060) | 2060 (2055 to 2063) |
| El Salvador | SLV (PAHO) | 2020 (2020 to 2020) | 2020 (2020 to 2020) | 2020 (2020 to 2020) | 2020 (2020 to 2020) | 2020 (2020 to 2020) | 2020 (2020 to 2020) | 2020 (2020 to 2020) |
| Somalia | SOM (EMRO) | after 2100 (after 2100 to after 2100) | after 2100 (after 2100 to after 2100) | 2075 (2066 to 2083) | after 2100 (after 2100 to after 2100) | after 2100 (after 2100 to after 2100) | 2075 (2067 to 2084) | 2079 (2072 to 2088) |
| Serbia | SRB (EURO) | 2018 (2010 to 2033) | 2018 (2010 to 2033) | 2018 (2010 to 2033) | 2018 (2010 to 2033) | 2018 (2010 to 2033) | 2018 (2010 to 2033) | 2018 (2010 to 2033) |
| South Sudan | SSD (AFRO) | after 2100 (after 2100 to after 2100) | after 2100 (after 2100 to after 2100) | 2080 (2071 to 2088) | after 2100 (after 2100 to after 2100) | after 2100 (after 2100 to after 2100) | 2080 (2072 to 2089) | 2083 (2077 to 2092) |
| Sao Tome and Principe | STP (AFRO) | 2038 (2030 to 2043) | 2038 (2030 to 2043) | 2038 (2030 to 2043) | 2038 (2030 to 2043) | 2038 (2030 to 2043) | 2038 (2030 to 2043) | 2038 (2030 to 2043) |
| Eswatini | SWZ (AFRO) | 2048 (2038 to 2060) | 2044 (2035 to 2054) | 2033 (2031 to 2034) | 2048 (2038 to 2060) | 2048 (2038 to 2060) | 2033 (2032 to 2034) | 2038 (2035 to 2041) |
| Syria | SYR (EMRO) | 2062 (2060 to 2067) | 2058 (2056 to 2062) | 2037 (2035 to 2042) | 2062 (2060 to 2067) | 2062 (2060 to 2067) | 2037 (2035 to 2042) | 2043 (2042 to 2045) |
| Chad | TCD (AFRO) | after 2100 (after 2100 to after 2100) | after 2100 (after 2100 to after 2100) | 2070 (2064 to 2074) | after 2100 (after 2100 to after 2100) | after 2100 (after 2100 to after 2100) | 2071 (2065 to 2075) | 2076 (2070 to 2080) |
| Togo | TGO (AFRO) | after 2100 (2082 to after 2100) | 2099 (2074 to after 2100) | 2057 (2051 to 2060) | after 2100 (2082 to after 2100) | after 2100 (2082 to after 2100) | 2058 (2051 to 2061) | 2062 (2053 to 2066) |
| Thailand | THA (SEARO) | 2022 (2022 to 2022) | 2022 (2022 to 2022) | 2022 (2022 to 2022) | 2022 (2022 to 2022) | 2022 (2022 to 2022) | 2022 (2022 to 2022) | 2022 (2022 to 2022) |
| Tajikistan | TJK (EURO) | 2032 (2029 to 2034) | 2032 (2029 to 2034) | 2032 (2029 to 2034) | 2032 (2029 to 2034) | 2032 (2029 to 2034) | 2032 (2029 to 2034) | 2032 (2029 to 2034) |

| Country | ISO country code (WHO region) | Status quo HepB3 & HepB-BD | HepB-BD scale-up (≥25%) | HepB-BD scale-up (≥90%) | HepB-BD disruptions (5%) | HepB-BD disruptions (20%) | delayed HepB-BD scale-up (2023 to 2030) | delayed HepB-BD scale-up (2025 to 2040) |
|---|---|---|---|---|---|---|---|---|
| Turkmenistan | TKM (EURO) | 2037 (2033 to 2039) | 2037 (2033 to 2039) | 2037 (2033 to 2039) | 2037 (2033 to 2039) | 2037 (2033 to 2039) | 2037 (2033 to 2039) | 2037 (2033 to 2039) |
| Timor-Leste | TLS (SEARO) | 2049 (2044 to 2052) | 2049 (2044 to 2052) | 2039 (2034 to 2044) | 2049 (2044 to 2052) | 2049 (2044 to 2052) | 2039 (2034 to 2044) | 2042 (2040 to 2045) |
| Tonga | TON (WPRO) | 2048 (2042 to 2058) | 2048 (2042 to 2058) | 2048 (2042 to 2058) | 2048 (2042 to 2058) | 2048 (2042 to 2059) | 2048 (2042 to 2058) | 2048 (2042 to 2058) |
| Tunisia | TUN (EMRO) | 2032 (2013 to 2033) | 2032 (2013 to 2033) | 2029 (2013 to 2030) | 2032 (2013 to 2033) | 2032 (2013 to 2033) | 2030 (2013 to 2031) | 2031 (2013 to 2032) |
| Tuvalu | TUV (WPRO) | 2039 (2035 to 2043) | 2039 (2035 to 2043) | 2039 (2035 to 2043) | 2039 (2035 to 2043) | 2039 (2035 to 2043) | 2039 (2035 to 2043) | 2039 (2035 to 2043) |
| Tanzania | TZA (AFRO) | 2071 (2052 to 2087) | 2060 (2047 to 2070) | 2034 (2033 to 2035) | 2071 (2052 to 2087) | 2071 (2052 to 2087) | 2034 (2034 to 2035) | 2042 (2039 to 2043) |
| Uganda | UGA (AFRO) | after 2100 (2074 to after 2100) | 2088 (2065 to 2100) | 2045 (2037 to 2049) | after 2100 (2074 to after 2100) | after 2100 (2074 to after 2100) | 2046 (2037 to 2050) | 2046 (2044 to 2052) |
| Ukraine | UKR (EURO) | 2043 (2009 to 2051) | 2043 (2009 to 2051) | 2034 (2009 to 2036) | 2043 (2009 to 2051) | 2043 (2009 to 2051) | 2034 (2009 to 2036) | 2038 (2009 to 2040) |
| Uzbekistan | UZB (EURO) | 2036 (2034 to 2039) | 2036 (2034 to 2039) | 2036 (2034 to 2039) | 2036 (2034 to 2039) | 2036 (2034 to 2039) | 2036 (2034 to 2039) | 2036 (2034 to 2039) |
| Venezuela | VEN (PAHO) | 2040 (2034 to 2044) | 2040 (2034 to 2044) | 2032 (2031 to 2033) | 2040 (2034 to 2044) | 2040 (2034 to 2044) | 2032 (2031 to 2033) | 2036 (2033 to 2037) |
| Viet Nam | VNM (WPRO) | 2052 (2045 to 2056) | 2052 (2045 to 2056) | 2047 (2042 to 2050) | 2052 (2045 to 2056) | 2052 (2045 to 2056) | 2047 (2042 to 2051) | 2047 (2043 to 2051) |
| Vanuatu | VUT (WPRO) | 2067 (2059 to 2076) | 2067 (2059 to 2076) | 2061 (2056 to 2066) | 2067 (2059 to 2076) | 2067 (2059 to 2076) | 2061 (2056 to 2066) | 2062 (2056 to 2067) |
| Samoa | WSM (WPRO) | 2069 (2062 to 2074) | 2069 (2062 to 2074) | 2054 (2043 to 2059) | 2069 (2062 to 2074) | 2070 (2063 to 2074) | 2054 (2043 to 2059) | 2055 (2045 to 2061) |
| Yemen | YEM (EMRO) | 2086 (2060 to after 2100) | 2070 (2053 to 2080) | 2035 (2034 to 2037) | 2086 (2060 to after 2100) | 2086 (2060 to after 2100) | 2035 (2034 to 2037) | 2043 (2041 to 2044) |
| South Africa | ZAF (AFRO) | after 2100 (2067 to after 2100) | 2090 (2061 to after 2100) | 2050 (2038 to 2054) | after 2100 (2067 to after 2100) | after 2100 (2067 to after 2100) | 2050 (2038 to 2055) | 2052 (2044 to 2059) |
| Zambia | ZMB (AFRO) | 2026 (2024 to 2031) | 2026 (2024 to 2031) | 2026 (2024 to 2031) | 2027 (2024 to 2031) | 2027 (2024 to 2031) | 2026 (2024 to 2031) | 2026 (2024 to 2031) |
| Zimbabwe | ZWE (AFRO) | 2050 (2044 to 2057) | 2046 (2041 to 2053) | 2034 (2033 to 2035) | 2050 (2044 to 2057) | 2050 (2044 to 2057) | 2034 (2034 to 2035) | 2040 (2038 to 2041) |
| Treatment level such that 80% of treatment-eligible individuals are in treatment in 2030 | | | | | | | | |
| Afghanistan | AFG (EMRO) | 2041 (2036 to 2047) | 2041 (2036 to 2047) | 2032 (2031 to 2033) | 2041 (2036 to 2047) | 2041 (2036 to 2047) | 2032 (2031 to 2033) | 2036 (2034 to 2038) |

| Country | ISO country code (WHO region) | Status quo HepB3 & HepB-BD | HepB-BD scale-up (≥25%) | HepB-BD scale-up (≥90%) | HepB-BD disruptions (5%) | HepB-BD disruptions (20%) | delayed HepB-BD scale-up (2023 to 2030) | delayed HepB-BD scale-up (2025 to 2040) |
|---|---|---|---|---|---|---|---|---|
| Angola | AGO (AFRO) | 2086 (2060 to 2095) | 2075 (2056 to 2082) | 2049 (2041 to 2051) | 2086 (2060 to 2095) | 2086 (2060 to 2095) | 2049 (2041 to 2052) | 2050 (2044 to 2054) |
| Albania | ALB (EURO) | 2022 (2009 to 2024) | 2022 (2009 to 2024) | 2022 (2009 to 2024) | 2022 (2009 to 2024) | 2022 (2009 to 2024) | 2022 (2009 to 2024) | 2022 (2009 to 2024) |
| Armenia | ARM (EURO) | 2028 (2018 to 2031) | 2028 (2018 to 2031) | 2028 (2018 to 2031) | 2028 (2018 to 2031) | 2028 (2018 to 2031) | 2028 (2018 to 2031) | 2028 (2018 to 2031) |
| Azerbaijan | AZE (EURO) | 2031 (2026 to 2034) | 2031 (2026 to 2034) | 2031 (2026 to 2034) | 2031 (2026 to 2034) | 2031 (2027 to 2034) | 2031 (2026 to 2034) | 2031 (2026 to 2034) |
| Burundi | BDI (AFRO) | 2046 (2041 to 2050) | 2041 (2037 to 2044) | 2031 (2031 to 2032) | 2046 (2041 to 2050) | 2046 (2041 to 2050) | 2032 (2032 to 2033) | 2037 (2035 to 2038) |
| Benin | BEN (AFRO) | 2079 (2065 to 2099) | 2071 (2061 to 2087) | 2051 (2045 to 2056) | 2079 (2065 to 2099) | 2079 (2065 to 2099) | 2052 (2045 to 2057) | 2052 (2046 to 2060) |
| Burkina Faso | BFA (AFRO) | 2045 (2041 to 2054) | 2043 (2039 to 2050) | 2034 (2033 to 2034) | 2045 (2041 to 2054) | 2045 (2041 to 2054) | 2034 (2033 to 2034) | 2039 (2037 to 2041) |
| Bangladesh | BGD (SEARO) | 2056 (2047 to 2071) | 2050 (2043 to 2060) | 2033 (2032 to 2034) | 2056 (2047 to 2071) | 2056 (2047 to 2071) | 2034 (2033 to 2034) | 2040 (2038 to 2042) |
| Bosnia and Herzegovina | BIH (EURO) | 2038 (2034 to 2042) | 2034 (2032 to 2036) | 2030 (2029 to 2030) | 2038 (2034 to 2042) | 2038 (2034 to 2042) | 2031 (2030 to 2031) | 2034 (2032 to 2035) |
| Belarus | BLR (EURO) | 2017 (2010 to 2024) | 2017 (2010 to 2024) | 2017 (2010 to 2024) | 2017 (2010 to 2024) | 2017 (2010 to 2025) | 2017 (2010 to 2024) | 2017 (2010 to 2024) |
| Belize | BLZ (PAHO) | 2016 (2010 to 2020) | 2016 (2010 to 2020) | 2016 (2010 to 2020) | 2016 (2010 to 2020) | 2016 (2010 to 2020) | 2016 (2010 to 2020) | 2016 (2010 to 2020) |
| Bolivia | BOL (PAHO) | 2030 (2026 to 2034) | 2029 (2026 to 2031) | 2027 (2026 to 2028) | 2030 (2026 to 2034) | 2030 (2026 to 2034) | 2029 (2026 to 2030) | 2030 (2026 to 2032) |
| Bhutan | BTN (SEARO) | 2041 (2031 to 2044) | 2041 (2031 to 2044) | 2038 (2030 to 2042) | 2041 (2031 to 2044) | 2041 (2031 to 2044) | 2038 (2031 to 2042) | 2040 (2031 to 2042) |
| Central African Republic | CAF (AFRO) | after 2100 (2076 to after 2100) | 2094 (2069 to after 2100) | 2059 (2053 to 2062) | after 2100 (2076 to after 2100) | after 2100 (2076 to after 2100) | 2060 (2054 to 2062) | 2063 (2055 to 2066) |
| China | CHN (WPRO) | 2025 (2021 to 2027) | 2025 (2021 to 2027) | 2025 (2021 to 2027) | 2026 (2021 to 2027) | 2026 (2021 to 2027) | 2025 (2021 to 2027) | 2025 (2021 to 2027) |
| Ivory Coast | CIV (AFRO) | 2080 (2054 to 2097) | 2074 (2052 to 2087) | 2049 (2038 to 2053) | 2080 (2054 to 2097) | 2080 (2054 to 2097) | 2049 (2038 to 2054) | 2050 (2043 to 2055) |
| Cameroon | CMR (AFRO) | 2072 (2054 to 2085) | 2064 (2050 to 2073) | 2039 (2035 to 2043) | 2072 (2054 to 2085) | 2072 (2054 to 2085) | 2039 (2035 to 2043) | 2044 (2042 to 2045) |
| Democratic Republic of the Congo | COD (AFRO) | 2066 (2055 to 2078) | 2060 (2051 to 2067) | 2037 (2035 to 2041) | 2066 (2055 to 2078) | 2066 (2055 to 2078) | 2037 (2035 to 2041) | 2044 (2042 to 2045) |

| Country | ISO country code (WHO region) | Status quo HepB3 & HepB-BD | HepB-BD scale-up (≥25%) | HepB-BD scale-up (≥90%) | HepB-BD disruptions (5%) | HepB-BD disruptions (20%) | delayed HepB-BD scale-up (2023 to 2030) | delayed HepB-BD scale-up (2025 to 2040) |
|---|---|---|---|---|---|---|---|---|
| Congo | COG (AFRO) | 2082 (2059 to after 2100) | 2073 (2056 to 2087) | 2050 (2043 to 2055) | 2082 (2059 to after 2100) | 2082 (2059 to after 2100) | 2051 (2043 to 2056) | 2052 (2045 to 2058) |
| Colombia | COL (PAHO) | 2016 (2009 to 2018) | 2016 (2009 to 2018) | 2016 (2009 to 2018) | 2016 (2009 to 2018) | 2016 (2009 to 2018) | 2016 (2009 to 2018) | 2016 (2009 to 2018) |
| Comoros | COM (AFRO) | 2055 (2049 to 2066) | 2051 (2046 to 2060) | 2036 (2035 to 2039) | 2055 (2049 to 2066) | 2055 (2049 to 2066) | 2036 (2035 to 2039) | 2042 (2040 to 2044) |
| Cape Verde | CPV (AFRO) | 2039 (2035 to 2042) | 2039 (2035 to 2042) | 2039 (2035 to 2042) | 2039 (2035 to 2042) | 2039 (2035 to 2042) | 2039 (2035 to 2042) | 2039 (2035 to 2042) |
| Cuba | CUB (PAHO) | 2005 (2005 to 2005) | 2005 (2005 to 2005) | 2005 (2005 to 2005) | 2005 (2005 to 2005) | 2005 (2005 to 2005) | 2005 (2005 to 2005) | 2005 (2005 to 2005) |
| Djibouti | DJI (EMRO) | 2037 (2029 to 2040) | 2037 (2029 to 2040) | 2037 (2029 to 2040) | 2037 (2029 to 2040) | 2037 (2029 to 2040) | 2037 (2029 to 2040) | 2037 (2029 to 2040) |
| Algeria | DZA (AFRO) | 2009 (2009 to 2011) | 2009 (2009 to 2011) | 2009 (2009 to 2011) | 2009 (2009 to 2011) | 2009 (2009 to 2011) | 2009 (2009 to 2011) | 2009 (2009 to 2011) |
| Ecuador | ECU (PAHO) | 2030 (2027 to 2034) | 2030 (2027 to 2034) | 2028 (2026 to 2029) | 2030 (2027 to 2034) | 2030 (2027 to 2034) | 2029 (2027 to 2030) | 2030 (2027 to 2032) |
| Egypt | EGY (EMRO) | 2022 (2022 to 2022) | 2022 (2022 to 2022) | 2022 (2022 to 2022) | 2022 (2022 to 2022) | 2022 (2022 to 2022) | 2022 (2022 to 2022) | 2022 (2022 to 2022) |
| Eritrea | ERI (AFRO) | 2045 (2036 to 2059) | 2041 (2034 to 2053) | 2033 (2031 to 2034) | 2045 (2036 to 2059) | 2045 (2036 to 2059) | 2033 (2032 to 2034) | 2037 (2034 to 2041) |
| Ethiopia | ETH (AFRO) | 2066 (2063 to 2073) | 2060 (2058 to 2066) | 2043 (2042 to 2045) | 2066 (2063 to 2073) | 2066 (2063 to 2073) | 2043 (2042 to 2045) | 2045 (2045 to 2046) |
| Fiji | FJI (WPRO) | 2028 (2023 to 2031) | 2028 (2023 to 2031) | 2028 (2023 to 2031) | 2028 (2024 to 2031) | 2029 (2024 to 2031) | 2028 (2023 to 2031) | 2028 (2023 to 2031) |
| Micronesia | FSM (WPRO) | 2043 (2037 to 2048) | 2043 (2037 to 2048) | 2037 (2033 to 2040) | 2043 (2037 to 2048) | 2043 (2037 to 2048) | 2037 (2034 to 2040) | 2040 (2036 to 2042) |
| Georgia | GEO (EURO) | 2033 (2029 to 2036) | 2033 (2029 to 2036) | 2033 (2029 to 2036) | 2033 (2029 to 2036) | 2033 (2029 to 2036) | 2033 (2029 to 2036) | 2033 (2029 to 2036) |
| Ghana | GHA (AFRO) | 2086 (2070 to 2097) | 2074 (2063 to 2083) | 2044 (2039 to 2049) | 2086 (2070 to 2097) | 2086 (2070 to 2097) | 2045 (2039 to 2049) | 2045 (2044 to 2050) |
| Guinea | GIN (AFRO) | after 2100 (2083 to after 2100) | 2092 (2076 to after 2100) | 2060 (2056 to 2063) | after 2100 (2083 to after 2100) | after 2100 (2083 to after 2100) | 2061 (2056 to 2064) | 2064 (2058 to 2068) |
| Gambia | GMB (AFRO) | 2056 (2037 to 2078) | 2051 (2035 to 2066) | 2034 (2031 to 2035) | 2056 (2037 to 2078) | 2056 (2037 to 2078) | 2034 (2032 to 2035) | 2040 (2035 to 2044) |
| Guinea-Bissau | GNB (AFRO) | 2073 (2055 to 2091) | 2066 (2052 to 2079) | 2044 (2038 to 2048) | 2073 (2055 to 2091) | 2073 (2055 to 2091) | 2044 (2038 to 2049) | 2045 (2043 to 2050) |

| Country | ISO country code (WHO region) | Status quo HepB3 & HepB-BD | HepB-BD scale-up (≥25%) | HepB-BD scale-up (≥90%) | HepB-BD disruptions (5%) | HepB-BD disruptions (20%) | delayed HepB-BD scale-up (2023 to 2030) | delayed HepB-BD scale-up (2025 to 2040) |
|---|---|---|---|---|---|---|---|---|
| Guatemala | GTM (PAHO) | 2012 (2011 to 2012) | 2012 (2011 to 2012) | 2012 (2011 to 2012) | 2012 (2011 to 2012) | 2012 (2011 to 2012) | 2012 (2011 to 2012) | 2012 (2011 to 2012) |
| Guyana | GUY (PAHO) | 2060 (2046 to 2070) | 2054 (2043 to 2061) | 2034 (2033 to 2035) | 2060 (2046 to 2070) | 2060 (2046 to 2070) | 2034 (2033 to 2035) | 2042 (2038 to 2043) |
| Honduras | HND (PAHO) | 2036 (2031 to 2039) | 2036 (2031 to 2039) | 2032 (2029 to 2033) | 2036 (2031 to 2039) | 2036 (2031 to 2039) | 2032 (2030 to 2033) | 2034 (2031 to 2036) |
| Haiti | HTI (PAHO) | 2066 (2054 to 2076) | 2058 (2050 to 2065) | 2035 (2034 to 2035) | 2066 (2054 to 2076) | 2066 (2054 to 2076) | 2035 (2034 to 2036) | 2043 (2041 to 2044) |
| Indonesia | IDN (SEARO) | 2047 (2045 to 2049) | 2047 (2045 to 2049) | 2044 (2041 to 2046) | 2047 (2045 to 2049) | 2047 (2045 to 2049) | 2044 (2041 to 2046) | 2044 (2042 to 2046) |
| India | IND (SEARO) | 2041 (2035 to 2045) | 2041 (2035 to 2045) | 2033 (2030 to 2034) | 2041 (2035 to 2045) | 2041 (2035 to 2045) | 2033 (2031 to 2034) | 2037 (2034 to 2040) |
| Iran | IRN (EMRO) | 2005 (2005 to 2005) | 2005 (2005 to 2005) | 2005 (2005 to 2005) | 2005 (2005 to 2005) | 2005 (2005 to 2005) | 2005 (2005 to 2005) | 2005 (2005 to 2005) |
| Iraq | IRQ (EMRO) | 2040 (2036 to 2046) | 2040 (2036 to 2046) | 2032 (2031 to 2033) | 2040 (2036 to 2046) | 2040 (2036 to 2046) | 2032 (2031 to 2033) | 2036 (2034 to 2037) |
| Jamaica | JAM (PAHO) | 2052 (2038 to 2059) | 2047 (2036 to 2053) | 2033 (2032 to 2034) | 2052 (2038 to 2059) | 2052 (2038 to 2059) | 2034 (2032 to 2034) | 2039 (2035 to 2041) |
| Jordan | JOR (EMRO) | 1997 (1988 to 2000) | 1997 (1988 to 2000) | 1997 (1988 to 2000) | 1997 (1988 to 2000) | 1997 (1988 to 2000) | 1997 (1988 to 2000) | 1997 (1988 to 2000) |
| Kenya | KEN (AFRO) | 2021 (2013 to 2022) | 2021 (2013 to 2022) | 2021 (2013 to 2022) | 2021 (2013 to 2022) | 2021 (2013 to 2022) | 2021 (2013 to 2022) | 2021 (2013 to 2022) |
| Kyrgyzstan | KGZ (EURO) | 2019 (2007 to 2026) | 2019 (2007 to 2026) | 2019 (2007 to 2026) | 2019 (2007 to 2026) | 2019 (2007 to 2026) | 2019 (2007 to 2026) | 2019 (2007 to 2026) |
| Cambodia | KHM (WPRO) | 2036 (2032 to 2038) | 2036 (2032 to 2038) | 2035 (2032 to 2037) | 2036 (2032 to 2038) | 2036 (2032 to 2038) | 2035 (2032 to 2037) | 2035 (2032 to 2037) |
| Kiribati | KIR (WPRO) | 2034 (2028 to 2037) | 2034 (2028 to 2037) | 2034 (2028 to 2037) | 2034 (2028 to 2037) | 2034 (2028 to 2037) | 2034 (2028 to 2037) | 2034 (2028 to 2037) |
| Laos | LAO (WPRO) | 2046 (2041 to 2049) | 2046 (2041 to 2049) | 2034 (2033 to 2035) | 2046 (2041 to 2049) | 2046 (2041 to 2049) | 2034 (2033 to 2035) | 2039 (2037 to 2041) |
| Liberia | LBR (AFRO) | 2096 (2074 to after 2100) | 2086 (2069 to 2100) | 2058 (2052 to 2060) | 2096 (2074 to after 2100) | 2096 (2074 to after 2100) | 2058 (2053 to 2061) | 2061 (2054 to 2065) |
| Sri Lanka | LKA (SEARO) | 2040 (2037 to 2050) | 2038 (2036 to 2045) | 2032 (2032 to 2033) | 2040 (2037 to 2050) | 2040 (2037 to 2050) | 2033 (2032 to 2034) | 2036 (2035 to 2039) |
| Lesotho | LSO (AFRO) | 2064 (2046 to 2082) | 2058 (2043 to 2070) | 2036 (2034 to 2041) | 2064 (2046 to 2082) | 2064 (2046 to 2082) | 2036 (2034 to 2041) | 2043 (2039 to 2045) |

| Country | ISO country code (WHO region) | Status quo HepB3 & HepB-BD | HepB-BD scale-up (≥25%) | HepB-BD scale-up (≥90%) | HepB-BD disruptions (5%) | HepB-BD disruptions (20%) | delayed HepB-BD scale-up (2023 to 2030) | delayed HepB-BD scale-up (2025 to 2040) |
|---|---|---|---|---|---|---|---|---|
| Morocco | MAR (EMRO) | 2005 (2005 to 2006) | 2005 (2005 to 2006) | 2005 (2005 to 2006) | 2005 (2005 to 2006) | 2005 (2005 to 2006) | 2005 (2005 to 2006) | 2005 (2005 to 2006) |
| Moldova | MDA (EURO) | 2039 (2037 to 2041) | 2039 (2037 to 2041) | 2039 (2037 to 2041) | 2039 (2037 to 2041) | 2039 (2037 to 2041) | 2039 (2037 to 2041) | 2039 (2037 to 2041) |
| Madagascar | MDG (AFRO) | 2098 (2091 to after 2100) | 2081 (2077 to 2083) | 2046 (2045 to 2047) | 2098 (2091 to after 2100) | 2098 (2091 to after 2100) | 2047 (2045 to 2048) | 2047 (2045 to 2048) |
| Marshall Islands | MHL (WPRO) | 2039 (2036 to 2042) | 2039 (2036 to 2042) | 2039 (2036 to 2042) | 2039 (2036 to 2042) | 2039 (2036 to 2042) | 2039 (2036 to 2042) | 2039 (2036 to 2042) |
| North Macedonia | MKD (EURO) | 2031 (2017 to 2035) | 2031 (2017 to 2035) | 2031 (2017 to 2035) | 2031 (2017 to 2035) | 2031 (2017 to 2035) | 2031 (2017 to 2035) | 2031 (2017 to 2035) |
| Mali | MLI (AFRO) | 2046 (2041 to 2052) | 2043 (2039 to 2048) | 2034 (2033 to 2034) | 2046 (2041 to 2052) | 2046 (2041 to 2052) | 2034 (2033 to 2035) | 2039 (2037 to 2041) |
| Myanmar | MMR (SEARO) | 2074 (2058 to 2096) | 2072 (2057 to 2091) | 2051 (2043 to 2057) | 2074 (2058 to 2096) | 2074 (2058 to 2096) | 2052 (2043 to 2058) | 2052 (2045 to 2061) |
| Mongolia | MNG (WPRO) | 2023 (2018 to 2025) | 2023 (2018 to 2025) | 2023 (2018 to 2025) | 2023 (2018 to 2026) | 2023 (2018 to 2026) | 2023 (2018 to 2025) | 2023 (2018 to 2025) |
| Mozambique | MOZ (AFRO) | 2078 (2071 to 2084) | 2068 (2063 to 2072) | 2042 (2037 to 2044) | 2078 (2071 to 2084) | 2078 (2071 to 2084) | 2042 (2037 to 2045) | 2045 (2044 to 2045) |
| Mauritania | MRT (AFRO) | 2074 (2059 to 2088) | 2066 (2055 to 2077) | 2042 (2036 to 2047) | 2074 (2059 to 2088) | 2074 (2059 to 2088) | 2042 (2036 to 2047) | 2045 (2043 to 2047) |
| Malawi | MWI (AFRO) | 2031 (2030 to 2033) | 2030 (2029 to 2031) | 2029 (2028 to 2029) | 2031 (2030 to 2033) | 2031 (2030 to 2033) | 2030 (2029 to 2030) | 2031 (2030 to 2032) |
| Namibia | NAM (AFRO) | 2031 (2027 to 2034) | 2031 (2027 to 2034) | 2029 (2026 to 2032) | 2031 (2027 to 2034) | 2031 (2027 to 2034) | 2030 (2027 to 2032) | 2031 (2027 to 2033) |
| Niger | NER (AFRO) | 2090 (2069 to after 2100) | 2080 (2064 to 2095) | 2055 (2049 to 2058) | 2090 (2069 to after 2100) | 2090 (2069 to after 2100) | 2055 (2050 to 2059) | 2058 (2050 to 2063) |
| Nigeria | NGA (AFRO) | 2100 (2092 to after 2100) | 2088 (2083 to 2098) | 2058 (2056 to 2061) | 2100 (2092 to after 2100) | 2100 (2092 to after 2100) | 2059 (2056 to 2062) | 2061 (2058 to 2066) |
| Nicaragua | NIC (PAHO) | 2003 (2003 to 2004) | 2003 (2003 to 2004) | 2003 (2003 to 2004) | 2003 (2003 to 2004) | 2003 (2003 to 2004) | 2003 (2003 to 2004) | 2003 (2003 to 2004) |
| Nepal | NPL (SEARO) | 2037 (2032 to 2044) | 2034 (2031 to 2038) | 2030 (2029 to 2031) | 2037 (2032 to 2044) | 2037 (2032 to 2044) | 2031 (2030 to 2032) | 2034 (2031 to 2036) |
| Pakistan | PAK (EMRO) | 2055 (2038 to 2061) | 2049 (2035 to 2053) | 2033 (2031 to 2033) | 2055 (2038 to 2061) | 2055 (2038 to 2061) | 2033 (2032 to 2034) | 2040 (2035 to 2041) |
| Peru | PER (PAHO) | 2008 (2008 to 2009) | 2008 (2008 to 2009) | 2008 (2008 to 2009) | 2008 (2008 to 2009) | 2008 (2008 to 2009) | 2008 (2008 to 2009) | 2008 (2008 to 2009) |

| Country | ISO country code (WHO region) | Status quo HepB3 & HepB-BD | HepB-BD scale-up (≥25%) | HepB-BD scale-up (≥90%) | HepB-BD disruptions (5%) | HepB-BD disruptions (20%) | delayed HepB-BD scale-up (2023 to 2030) | delayed HepB-BD scale-up (2025 to 2040) |
|---|---|---|---|---|---|---|---|---|
| Philippines | PHL (WPRO) | 2054 (2049 to 2057) | 2054 (2049 to 2057) | 2041 (2038 to 2044) | 2054 (2049 to 2057) | 2054 (2049 to 2058) | 2041 (2038 to 2044) | 2044 (2042 to 2045) |
| Papua New Guinea | PNG (WPRO) | 2075 (2066 to 2081) | 2075 (2066 to 2081) | 2051 (2045 to 2054) | 2075 (2066 to 2081) | 2075 (2066 to 2081) | 2051 (2045 to 2055) | 2052 (2047 to 2056) |
| Korea, North | PRK (SEARO) | 2039 (2036 to 2040) | 2039 (2036 to 2040) | 2039 (2036 to 2040) | 2039 (2036 to 2040) | 2039 (2036 to 2040) | 2039 (2036 to 2040) | 2039 (2036 to 2040) |
| Paraguay | PRY (PAHO) | 2053 (2040 to 2061) | 2048 (2037 to 2054) | 2033 (2032 to 2034) | 2053 (2040 to 2061) | 2053 (2040 to 2061) | 2034 (2033 to 2034) | 2040 (2036 to 2041) |
| Rwanda | RWA (AFRO) | 2038 (2034 to 2040) | 2034 (2032 to 2035) | 2029 (2029 to 2030) | 2038 (2034 to 2040) | 2038 (2034 to 2040) | 2031 (2030 to 2031) | 2034 (2032 to 2034) |
| Sudan | SDN (EMRO) | 2058 (2048 to 2065) | 2053 (2045 to 2058) | 2034 (2033 to 2034) | 2058 (2048 to 2065) | 2058 (2048 to 2065) | 2034 (2034 to 2034) | 2041 (2039 to 2042) |
| Senegal | SEN (AFRO) | 2046 (2039 to 2049) | 2046 (2039 to 2049) | 2039 (2034 to 2043) | 2046 (2039 to 2049) | 2046 (2039 to 2049) | 2039 (2035 to 2043) | 2041 (2037 to 2044) |
| Solomon Islands | SLB (WPRO) | 2043 (2040 to 2046) | 2043 (2040 to 2046) | 2036 (2034 to 2040) | 2043 (2040 to 2046) | 2043 (2040 to 2046) | 2036 (2034 to 2040) | 2040 (2037 to 2041) |
| Sierra Leone | SLE (AFRO) | 2070 (2064 to 2078) | 2065 (2060 to 2071) | 2050 (2046 to 2053) | 2070 (2064 to 2078) | 2070 (2064 to 2078) | 2050 (2047 to 2053) | 2051 (2047 to 2055) |
| El Salvador | SLV (PAHO) | 2020 (2020 to 2020) | 2020 (2020 to 2020) | 2020 (2020 to 2020) | 2020 (2020 to 2020) | 2020 (2020 to 2020) | 2020 (2020 to 2020) | 2020 (2020 to 2020) |
| Somalia | SOM (EMRO) | after 2100 (2089 to after 2100) | 2094 (2081 to after 2100) | 2062 (2057 to 2064) | after 2100 (2089 to after 2100) | after 2100 (2089 to after 2100) | 2062 (2058 to 2065) | 2067 (2060 to 2069) |
| Serbia | SRB (EURO) | 2018 (2010 to 2027) | 2018 (2010 to 2027) | 2018 (2010 to 2027) | 2018 (2010 to 2027) | 2018 (2010 to 2027) | 2018 (2010 to 2027) | 2018 (2010 to 2027) |
| South Sudan | SSD (AFRO) | after 2100 (2094 to after 2100) | 2095 (2086 to after 2100) | 2064 (2061 to 2068) | after 2100 (2094 to after 2100) | after 2100 (2094 to after 2100) | 2065 (2062 to 2069) | 2069 (2065 to 2073) |
| Sao Tome and Principe | STP (AFRO) | 2030 (2026 to 2034) | 2030 (2026 to 2034) | 2030 (2026 to 2034) | 2030 (2026 to 2034) | 2030 (2026 to 2034) | 2030 (2026 to 2034) | 2030 (2026 to 2034) |
| Eswatini | SWZ (AFRO) | 2040 (2033 to 2051) | 2037 (2032 to 2047) | 2031 (2030 to 2033) | 2040 (2033 to 2051) | 2040 (2033 to 2051) | 2032 (2031 to 2033) | 2035 (2032 to 2039) |
| Syria | SYR (EMRO) | 2051 (2047 to 2055) | 2047 (2043 to 2051) | 2034 (2033 to 2035) | 2051 (2047 to 2055) | 2051 (2047 to 2055) | 2034 (2034 to 2035) | 2039 (2038 to 2041) |
| Chad | TCD (AFRO) | after 2100 (2082 to after 2100) | 2092 (2075 to after 2100) | 2060 (2055 to 2063) | after 2100 (2082 to after 2100) | after 2100 (2082 to after 2100) | 2061 (2055 to 2064) | 2063 (2057 to 2068) |
| Togo | TGO (AFRO) | 2080 (2062 to 2097) | 2071 (2058 to 2084) | 2049 (2042 to 2053) | 2080 (2062 to 2097) | 2080 (2062 to 2097) | 2050 (2042 to 2053) | 2050 (2044 to 2055) |

| Country | ISO country code (WHO region) | Status quo HepB3 & HepB-BD | HepB-BD scale-up (≥25%) | HepB-BD scale-up (≥90%) | HepB-BD disruptions (5%) | HepB-BD disruptions (20%) | delayed HepB-BD scale-up (2023 to 2030) | delayed HepB-BD scale-up (2025 to 2040) |
|---|---|---|---|---|---|---|---|---|
| Thailand | THA (SEARO) | 2022 (2022 to 2022) | 2022 (2022 to 2022) | 2022 (2022 to 2022) | 2022 (2022 to 2022) | 2022 (2022 to 2022) | 2022 (2022 to 2022) | 2022 (2022 to 2022) |
| Tajikistan | TJK (EURO) | 2030 (2027 to 2031) | 2030 (2027 to 2031) | 2030 (2027 to 2031) | 2030 (2027 to 2031) | 2030 (2027 to 2031) | 2030 (2027 to 2031) | 2030 (2027 to 2031) |
| Turkmenistan | TKM (EURO) | 2034 (2030 to 2036) | 2034 (2030 to 2036) | 2034 (2030 to 2036) | 2034 (2030 to 2036) | 2034 (2030 to 2036) | 2034 (2030 to 2036) | 2034 (2030 to 2036) |
| Timor-Leste | TLS (SEARO) | 2042 (2037 to 2046) | 2042 (2037 to 2046) | 2033 (2031 to 2036) | 2042 (2037 to 2046) | 2042 (2037 to 2046) | 2034 (2032 to 2036) | 2038 (2035 to 2040) |
| Tonga | TON (WPRO) | 2043 (2040 to 2047) | 2043 (2040 to 2047) | 2043 (2040 to 2047) | 2043 (2040 to 2047) | 2043 (2040 to 2047) | 2043 (2040 to 2047) | 2043 (2040 to 2047) |
| Tunisia | TUN (EMRO) | 2030 (2013 to 2031) | 2030 (2013 to 2031) | 2028 (2013 to 2029) | 2030 (2013 to 2031) | 2030 (2013 to 2031) | 2029 (2013 to 2030) | 2030 (2013 to 2031) |
| Tuvalu | TUV (WPRO) | 2037 (2032 to 2039) | 2037 (2032 to 2039) | 2037 (2032 to 2039) | 2037 (2032 to 2039) | 2037 (2032 to 2039) | 2037 (2032 to 2039) | 2037 (2032 to 2039) |
| Tanzania | TZA (AFRO) | 2051 (2040 to 2059) | 2046 (2037 to 2053) | 2033 (2031 to 2034) | 2051 (2040 to 2059) | 2051 (2040 to 2059) | 2033 (2032 to 2034) | 2039 (2036 to 2041) |
| Uganda | UGA (AFRO) | 2079 (2061 to 2090) | 2068 (2056 to 2076) | 2037 (2035 to 2042) | 2079 (2061 to 2090) | 2079 (2061 to 2090) | 2037 (2035 to 2042) | 2044 (2042 to 2045) |
| Ukraine | UKR (EURO) | 2036 (2009 to 2040) | 2036 (2009 to 2040) | 2032 (2009 to 2033) | 2036 (2009 to 2040) | 2036 (2009 to 2040) | 2032 (2009 to 2033) | 2034 (2009 to 2037) |
| Uzbekistan | UZB (EURO) | 2034 (2032 to 2037) | 2034 (2032 to 2037) | 2034 (2032 to 2037) | 2034 (2032 to 2037) | 2034 (2032 to 2037) | 2034 (2032 to 2037) | 2034 (2032 to 2037) |
| Venezuela | VEN (PAHO) | 2035 (2030 to 2037) | 2035 (2030 to 2037) | 2030 (2028 to 2031) | 2035 (2030 to 2037) | 2035 (2030 to 2037) | 2031 (2029 to 2032) | 2033 (2030 to 2034) |
| Viet Nam | VNM (WPRO) | 2046 (2041 to 2049) | 2046 (2041 to 2049) | 2042 (2038 to 2044) | 2046 (2041 to 2049) | 2046 (2041 to 2049) | 2042 (2038 to 2044) | 2043 (2039 to 2045) |
| Vanuatu | VUT (WPRO) | 2058 (2054 to 2062) | 2058 (2054 to 2062) | 2055 (2051 to 2058) | 2058 (2054 to 2062) | 2058 (2054 to 2062) | 2055 (2051 to 2058) | 2055 (2051 to 2058) |
| Samoa | WSM (WPRO) | 2055 (2048 to 2060) | 2055 (2048 to 2060) | 2042 (2037 to 2045) | 2055 (2048 to 2060) | 2056 (2048 to 2060) | 2042 (2037 to 2045) | 2044 (2041 to 2045) |
| Yemen | YEM (EMRO) | 2058 (2046 to 2068) | 2053 (2042 to 2059) | 2034 (2032 to 2034) | 2058 (2046 to 2068) | 2058 (2046 to 2068) | 2034 (2033 to 2034) | 2041 (2037 to 2042) |
| South Africa | ZAF (AFRO) | 2072 (2052 to 2089) | 2063 (2049 to 2075) | 2038 (2034 to 2044) | 2072 (2052 to 2089) | 2072 (2052 to 2089) | 2038 (2034 to 2044) | 2044 (2041 to 2045) |
| Zambia | ZMB (AFRO) | 2025 (2024 to 2027) | 2025 (2024 to 2027) | 2025 (2024 to 2027) | 2026 (2024 to 2027) | 2026 (2024 to 2027) | 2025 (2024 to 2027) | 2025 (2024 to 2027) |

| Country | ISO country code (WHO region) | Status quo HepB3 & HepB-BD | HepB-BD scale-up (≥25%) | HepB-BD scale-up (≥90%) | HepB-BD disruptions (5%) | HepB-BD disruptions (20%) | delayed HepB-BD scale-up (2023 to 2030) | delayed HepB-BD scale-up (2025 to 2040) |
|---|---|---|---|---|---|---|---|---|
| Zimbabwe | ZWE (AFRO) | 2042 (2038 to 2048) | 2039 (2036 to 2044) | 2033 (2032 to 2033) | 2042 (2038 to 2048) | 2042 (2038 to 2048) | 2033 (2033 to 2034) | 2037 (2035 to 2039) |

# Supplementary References


1. Nayagam, S., *et al.* Requirements for global elimination of hepatitis B: a modelling study. *The Lancet Infectious Diseases* **16**, 1399–1408 (2016).
2. de Villiers, M.J., *et al.* Modelling hepatitis B virus infection and impact of timely birth dose vaccine: A comparison of two simulation models. *PLOS ONE* **15**, e0237525 (2020).
3. Toni, T., Welch, D., Strelkowa, N., Ipsen, A. & Stumpf, M.P. Approximate Bayesian computation scheme for parameter inference and model selection in dynamical systems. *Journal of the Royal Society Interface* **6**, 187–202 (2009).
4. Razavi-Shearer, D., *et al.* Global prevalence, treatment, and prevention of hepatitis B virus infection in 2016: a modelling study. *The Lancet Gastroenterology & Hepatology* **3**, 383–403 (2018).
5. *Global hepatitis report 2017*, (World Health Organization, Geneva, 2017).
6. Combating hepatitis B and C to reach elimination by 2030. in *Advocacy Brief* (World Health Organization, Geneva, Switzerland, 2016).
7. World Health Statistics 2011. (World Health Organization, Geneva, Switzerland, 2011).
8. Ott, J.J., Stevens, G.A. & Wiersma, S.T. The risk of perinatal hepatitis B virus transmission: hepatitis B e-antigen (HBeAg) prevalence estimates for all world regions. *BMC Infectious Diseases* **12**(2012).
9. Ott, J.J., Stevens, G.A., Groeger, J. & Wiersma, S.T. Global epidemiology of hepatitis B virus infection: new estimates of age-specific HBsAg seroprevalence and endemicity. *Vaccine* **30**, 2212–2219 (2012).
10. Cui, F., *et al.* Prevention of Chronic Hepatitis B after 3 Decades of Escalating Vaccination Policy, China. *Emerging infectious diseases* **23**, 765-772 (2017).
