# Supplementary material for: The impact of the timely birth dose vaccine on the global elimination of hepatitis B
Source: Nat Commun. 2021 Oct 28;12:6223. doi: 10.1038/s41467-021-26475-6 (PMC8553835; doi:10.1038/s41467-021-26475-6)
Supplement: Supplementary file 2 — Reporting Summary [file 41467_2021_26475_MOESM2_ESM.pdf]

nature portfolio

Corresponding author(s): Margaret de Villiers

Last updated by author(s): Sep 24, 2021

Reporting Summary

Nature Portfolio wishes to improve the reproducibility of the work that we publish. This form provides structure for consistency and transparency in reporting. For further information on Nature Portfolio policies, see our Editorial Policies and the Editorial Policy Checklist.

Statistics

For all statistical analyses, confirm that the following items are present in the figure legend, table legend, main text, or Methods section.

n/a	Confirmed	
☐	☒	The exact sample size (*n*) for each experimental group/condition, given as a discrete number and unit of measurement
☐	☒	A statement on whether measurements were taken from distinct samples or whether the same sample was measured repeatedly
☒	☐	The statistical test(s) used AND whether they are one- or two-sided<br>*Only common tests should be described solely by name; describe more complex techniques in the Methods section.*
☒	☐	A description of all covariates tested
☒	☐	A description of any assumptions or corrections, such as tests of normality and adjustment for multiple comparisons
☐	☒	A full description of the statistical parameters including central tendency (e.g. means) or other basic estimates (e.g. regression coefficient) AND variation (e.g. standard deviation) or associated estimates of uncertainty (e.g. confidence intervals)
☒	☐	For null hypothesis testing, the test statistic (e.g. *F*, *t*, *r*) with confidence intervals, effect sizes, degrees of freedom and *P* value noted<br>*Give P values as exact values whenever suitable.*
☐	☒	For Bayesian analysis, information on the choice of priors and Markov chain Monte Carlo settings
☒	☐	For hierarchical and complex designs, identification of the appropriate level for tests and full reporting of outcomes
☒	☐	Estimates of effect sizes (e.g. Cohen's *d*, Pearson's *r*), indicating how they were calculated

Our web collection on statistics for biologists contains articles on many of the points above.

Software and code

Policy information about availability of computer code

Data collection	No software was used to collect data from the internet.
Data analysis	All of the analyses were performed in MATLAB version R2020b (including model fitting; see De Villiers et al. 2020 for the fitting algorithm), except the drawing of the maps, which was done in with the function ggplot2 version 3.3.5 in R version 4.1.1 (2021-08-10) using a shape file from the R package rnaturalearth version 0.1.0.<br>MATLAB scripts for running the analyses and generating the results are available at Zenodo (10.5281/zenodo.5177261).

For manuscripts utilizing custom algorithms or software that are central to the research but not yet described in published literature, software must be made available to editors and reviewers. We strongly encourage code deposition in a community repository (e.g. GitHub). See the Nature Portfolio guidelines for submitting code & software for further information.

Data

Policy information about availability of data

All manuscripts must include a data availability statement. This statement should provide the following information, where applicable:

- Accession codes, unique identifiers, or web links for publicly available datasets
- A description of any restrictions on data availability
- For clinical datasets or third party data, please ensure that the statement adheres to our policy

The data sets for running the scripts to generate the results are available at Zenodo (10.5281/zenodo.5177261). This includes demographic data from the United Nations' 2019 World Population Prospects (Age-specific Fertility Rates, Sex Ratio at Birth, Annual Population by Age – Female, Annual Population by Age – Male, Net Migration Rate, Percentage of Female Deaths by Broad Age Groups, and Percentage of Male Deaths by Broad Age Groups at https://population.un.org/wpp/Download/Standard/, accessed in October 2019), historical vaccination coverage from WHO/UNICEF Estimates of National Immunization Coverage (WUENIC)

released in July 2020 (WHO-UNICEF estimates of HepB3 coverage at https://apps.who.int/immunization_monitoring/globalsummary/timeseries/tswucoveragehepb3.html and WHO-UNICEF estimates of HepB_BD coverage at https://apps.who.int/immunization_monitoring/globalsummary/timeseries/tswucoveragehepb_bd.html, accessed in July 2020), and HBsAg prevalence data from the WHO HBsAg dashboard (Hepatitis b surface antigen estimates and number of carriers in 2015 in the general population and Hepatitis b surface antigen estimates and number of carriers in 2015 in the under 5 years of age, currently available at http://situatedlaboratories.net/who-hepB-dashboard/src/#global-strategies as of September 2021). Also included are HepB3 and timely HepB-BD vaccination coverage datasets and HBsAg prevalence datasets from Cui et al. (2017) for China, country-specific HBsAg prevalence data from Razavi-Shearer et al. (2018) and disability weights from Vos et al. (2017).

Field-specific reporting

Please select the one below that is the best fit for your research. If you are not sure, read the appropriate sections before making your selection.

[x] Life sciences   [ ] Behavioural & social sciences   [ ] Ecological, evolutionary & environmental sciences

For a reference copy of the document with all sections, see nature.com/documents/nr-reporting-summary-flat.pdf

Life sciences study design

All studies must disclose on these points even when the disclosure is negative.

Sample size	A sample size calculation was not required as this was an analysis of secondary data.
Data exclusions	The data exclusion criteria were pre-established. Only lower income and lower middle income countries were included in the analyses (i.e. high-income and upper-middle income countries were excluded from the analyses), since the lower income countries are the countries that have the least resources to reduce hepatitis B infections in their populations and therefore tend to have higher hepatitis B prevalences. It is therefore in these countries that funders are most interested in understanding the dynamics of hepatitis B.
Replication	The model is deterministic, so given a set of input data (particles, demography, population HBsAg prevalence, etc.), the model will generate a fixed output.
Randomization	A set of 200 particles (independent draws) from the posterior distribution was generated for each country using the ABC SMC algorithm, and these 200 particles were then used to generate a set of 200 model outcomes for that country.
Blinding	Blinding is not relevant to this study, since all subjects (the countries in these analyses) were analysed in exactly the same way (the same MATLAB code was applied to all countries), irrespective of the treatment administered (the vaccination scenario applied to the country in these analyses).

Reporting for specific materials, systems and methods

We require information from authors about some types of materials, experimental systems and methods used in many studies. Here, indicate whether each material, system or method listed is relevant to your study. If you are not sure if a list item applies to your research, read the appropriate section before selecting a response.

Materials & experimental systems

n/a	Involved in the study	
[x]	[ ]	Antibodies
[x]	[ ]	Eukaryotic cell lines
[x]	[ ]	Palaeontology and archaeology
[x]	[ ]	Animals and other organisms
[x]	[ ]	Human research participants
[x]	[ ]	Clinical data
[x]	[ ]	Dual use research of concern

Methods

n/a	Involved in the study	
[x]	[ ]	ChIP-seq
[x]	[ ]	Flow cytometry
[x]	[ ]	MRI-based neuroimaging
